# Interrogating the Crucial Interactions at Play in the Chiral Cation-Directed Enantioselective Borylation of Arenes

**DOI:** 10.1021/acscatal.3c03384

**Published:** 2023-09-22

**Authors:** Kristaps Ermanis, David C. Gibson, Georgi R. Genov, Robert J. Phipps

**Affiliations:** † School of Chemistry, University of Nottingham, University Park, Nottingham NG7 2RD, United Kingdom; ‡ Yusuf Hamied Department of Chemistry, University of Cambridge, Lensfield Road, Cambridge CB2 1EW, United Kingdom

**Keywords:** C–H borylation, iridium, chiral cation, noncovalent interaction, computational chemistry

## Abstract

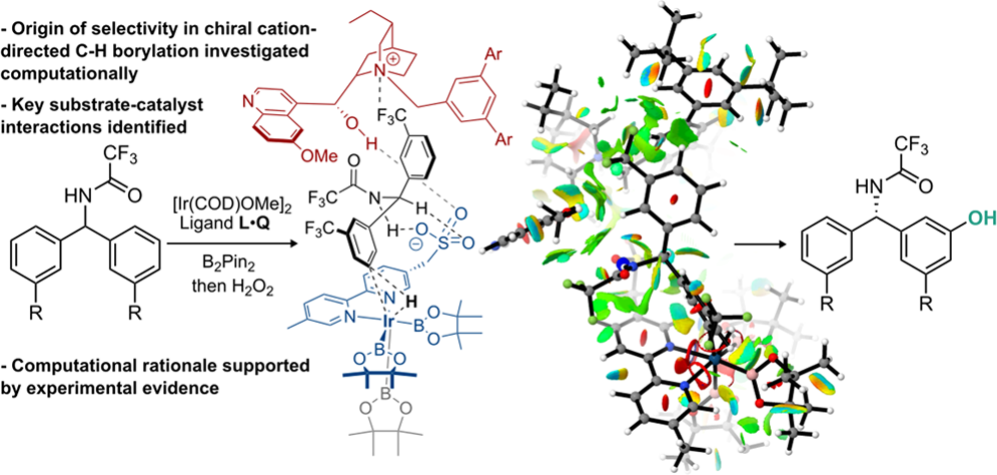

Rendering a common ligand scaffold anionic and then pairing it with a chiral cation represents an alternative strategy for developing enantioselective versions of challenging transformations, as has been recently demonstrated in the enantioselective borylation of arenes using a quinine-derived chiral cation. A significant barrier to the further generalization of this approach is the lack of understanding of the specific interactions involved between the cation, ligand, and substrate, given the complexity of the system. We have embarked on a detailed computational study probing the mechanism, the key noncovalent interactions involved, and potential origin of selectivity for the desymmetrizing borylation of two distinct classes of substrate. We describe a deconstructive, stepwise approach to tackling this complex challenge, which involves building up a detailed understanding of the pairwise components of the nominally three component system before combining together into the full 263-atom reactive complex. This approach has revealed substantial differences in the noncovalent interactions occurring at the stereodetermining transition state for C–H oxidative addition to iridium for the two substrate classes. Each substrate engages in a unique mixture of diverse interactions, a testament to the rich and privileged structure of the cinchona alkaloid-derived chiral cations. Throughout the study, experimental support is provided, and this culminates in the discovery that prochiral phosphine oxide substrates, lacking hydrogen bond donor functionality, can also give very encouraging levels of enantioselectivity, potentially through direct interactions with the chiral cation. We envisage that the findings in this study will spur further developments in using chiral cations as controllers in asymmetric transition-metal catalysis.

## Introduction

Chiral cations have been used extensively to induce asymmetry in organocatalytic reactions.^1^ Those derived from members of the cinchona family of alkaloids have constituted a particularly rich resource and these chiral cations are among the most widely used.^2^ Obtained from quaternization of the quinuclidine nitrogen, rapid development occurred in the 1980s based on asymmetric alkylation using phase-transfer catalysis.^3^ Despite subsequent extensive application in organocatalysis, it is notable that chiral cations have only rarely been used in combination with transition-metal catalysis, despite the extensive and diverse reaction mechanisms that transition-metal catalysts have the capability to undergo.^4^ Leading examples where this has been explored include Ooi’s covalent incorporation of a BINOL-derived chiral cation into the structure of a phosphine ligand for palladium-catalyzed allylic alkylation,^5^ as well as asymmetric oxidation reactions in which bisguanidinium chiral cations are paired with anionic diphosphatobisperoxotungstate and peroxomolybdate complexes from Tan and co-workers.^6,7^

**Figure 1:**
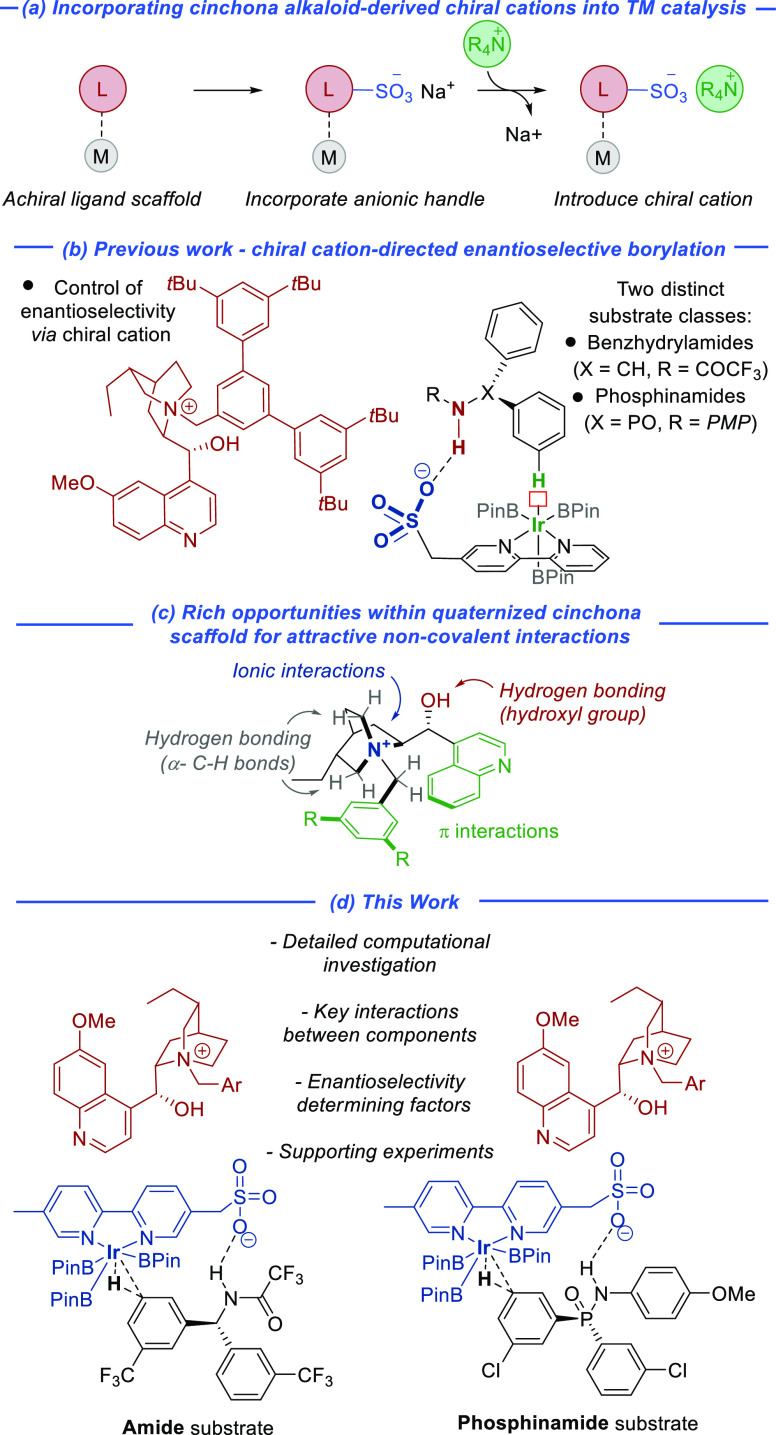
Background to this work and outline of study.

We recently sought to allow chiral cations derived from the privileged cinchona alkaloid framework to be combined with mainstream transition-metal-catalyzed processes, with a particular focus on those reactions that are challenging to render enantioselective using conventional approaches. For context, the neutral cinchona scaffold has proved outstanding when acting as a ligand for certain transition metals, most notably in the Sharpless asymmetric dihydroxylation.^8^ But for many transition metals, strongly basic amine ligands are incompatible, precluding use of the neutral cinchona scaffold as a chiral controller in all but a few situations. Approaches have also been developed whereby the neutral cinchona scaffold is covalently integrated into a conventional ligand such as a phosphine.^9^ A drawback of this strategy is that the ideal ligand structure for enabling reactivity may need to be compromised to make the covalent incorporation of the cinchona scaffold feasible. Recently, one of our groups disclosed a conceptually distinct strategy in which the ideal ligand for the desired reaction is rendered anionic by appendage of a sulfonate group, remote enough not to interfere with chemistry at the metal center but close enough to provide a chiral environment when paired with a cinchona alkaloid-derived cation.^10^ Crucially, in this strategy, the ligand structure (enabling reactivity) and the chiral controller (inducing enantioselectivity) are decoupled from one another, allowing each to be modulated and varied separately before being united through an ion-pairing interaction (Figure 1a).^11^ We first demonstrated that this approach was applicable to controlling asymmetry in iridium-catalyzed C–H borylation of two distinct prochiral substrate classes, in which a chiral center is introduced at either carbon or phosphorus atom (Figure 1b).^10a^ This was achieved by the use of a sulfonated bipyridine ligand for iridium. Subsequently, we have also applied the same strategy to Rh-catalyzed C–H amination and aziridination using a sulfonated version of the esp ligand scaffold.^12^ In both cases, these reactions utilize ligands for which conventional covalent modification of the ligand structure in order to incorporate chiral information has proved to be extremely challenging.

The successful application of the chiral cation strategy to two quite different and challenging metal-catalyzed transformations using unrelated ligand scaffolds gives us encouragement that our approach has the potential to be general and broadly applicable. Despite this success, the precise way that the chiral cation induces enantioselectivity in these reactions remains enigmatic. Our pragmatic approach thus far has been to design the system such that the rich structure of the cinchona-derived chiral cation should be in close proximity to the formation of the new bond at the transition state for the enantiodetermining step of the mechanism. The cinchona-derived cation provides numerous opportunities for engagement in attractive noncovalent interactions through ionic interactions, hydrogen bonding, and π interactions (Figure 1c).^ ^2a,2c,13^^ In this way, it is hoped that a combination of attractive noncovalent interactions and steric effects will provide the necessary discrimination. While this approach has served us well so far, we are mindful that in order to generalize this methodology and apply it more widely it is very important to develop an understanding of the precise nature of cation–substrate and cation–ligand interactions that occur in the successful examples so that these may be used as design principles in the future. Previous hypotheses have been advanced regarding the origins of selectivity in reactions involving cinchona alkaloid-derived cations, but these have typically been in the context of asymmetric phase-transfer catalysis where the interaction of the chiral cation with the anionic nucleophile is key to determining the outcome.^ ^3c,13^,14^ Undeniably useful for alkylation reactions, these models have reduced relevance to our transformations, whereby the hypothesized primary ionic interaction is between the ligand and the cation, with the enantiodetermining bond-forming process occurring at the transition-metal center. Herein, we describe a detailed computational study on the mechanism, essential noncovalent interactions, and the possible origin of selectivity for the desymmetrizing borylation of amide and phosphinamide substrates, together with supporting experimental studies (Figure 1d). Undertaking such a computational study presents unique challenges, due to the combination of system size (>260 atoms), conformational flexibility, presence of transition-metal center, and diversity of noncovalent interactions involved. Indeed, even detailed DFT studies of large, purely organic catalysts are rare.^15^ While the size and complexity of this system make this a particularly challenging goal, insights relating to the diversity of catalyst–substrate interactions will be crucial in further extending the scope of both this enantioselective borylation reaction and also the general concept of using chiral cations to control enantioselectivity in transition-metal catalysis.

## Computational Methods

Conformational searches were carried out using Macromodel v12.3 and OPLS3 force field.^16^ The conformational search algorithm used was a 50/50 mixture of Monte Carlo/Low-Mode Following Algorithm.^17^ All DFT calculations were done using Gaussian 16 rev A.03.^18^ All geometry optimizations were done with B3LYP functional^19^ and 6-31G* basis set,^20^ with SDD basis set on iridium.^21^ The optimizations were initially done in gas phase, after which key structures were reoptimized with the SMD(diethyl ether) solvent model.^22^ For computational convenience, we employed diethyl ether solvent parameters in place of CPME, since both Gaussian and ORCA do not support CPME parameters. Notably, diethyl ether possesses a dielectric constant closely resembling that of CPME. Previous experimental findings have demonstrated comparable enantioselectivity between the two solvents, with diethyl ether exhibiting diminished reactivity.^10a^ All of the gas phase and solvent-optimized structures were confirmed with frequency calculations to check that no imaginary frequency or just one imaginary frequency is present for ground states and TSs, respectively. Single-point energy calculations were run with M06 functional,^23^ def2-TZVP basis set,^24^ using the SMD(diethyl ether) solvent model.

## Results and Discussion

### Model Studies

**Figure 2:**
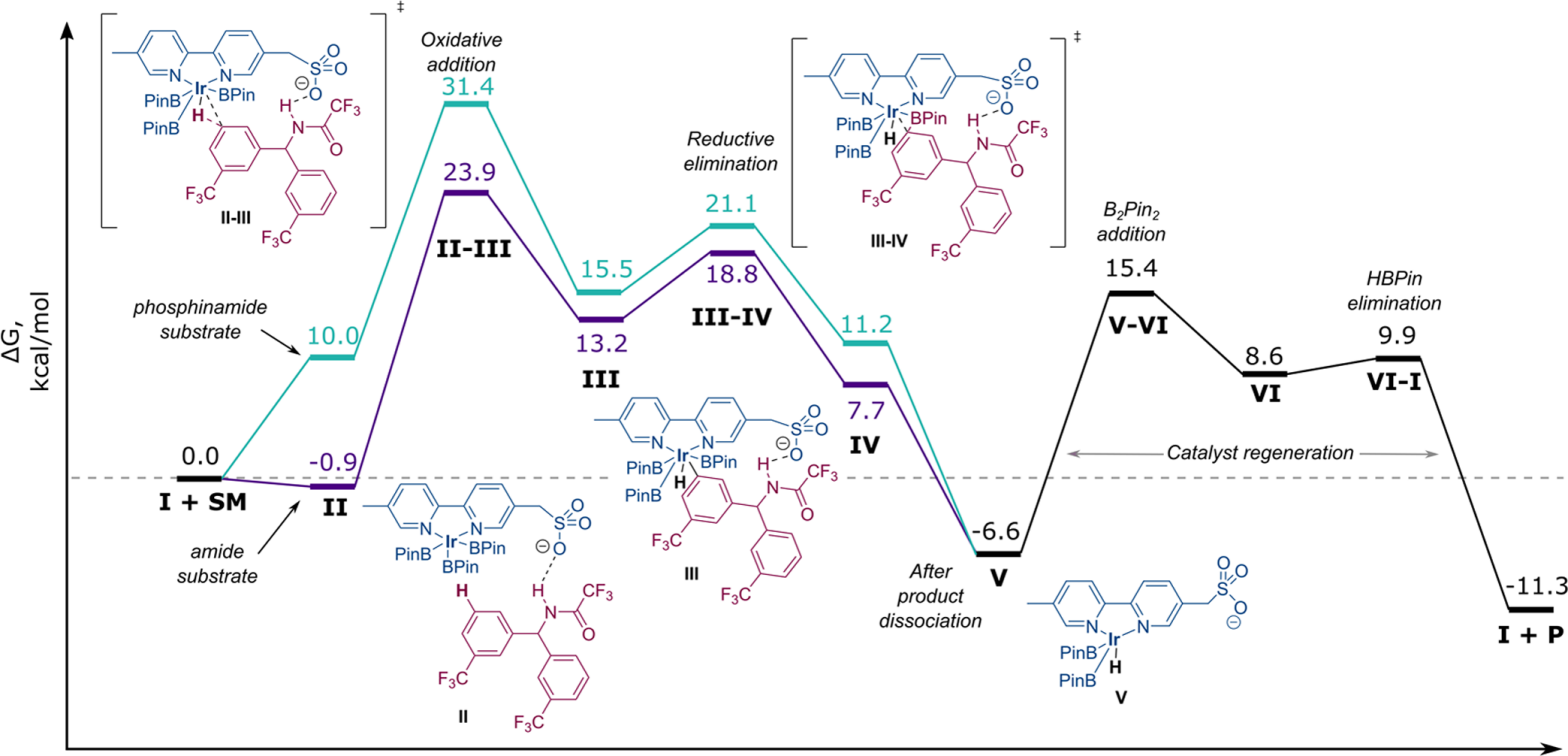
Model study of the Ir-catalyzed borylation pathway with an amide substrate (magenta) and a phosphinamide substrate (cyan).

The remarkable impact that iridium-catalyzed arene borylation has had since its first development two decades ago^25^ has meant that mechanistic studies soon followed and that for the most common bipyridine ligand system, mechanism is well established.^26^ In addition to experimental mechanistic studies, there have also been several primarily computational studies that explore the postulated mechanism and selectivity using DFT methods.^27^ Increasingly, reports of new borylation catalysts or methods include computational elements.^28^ The catalytic cycle typically commences with a borylated Ir complex, produced from the precatalyst, and when bipyridine ligands are used, it is generally accepted that an Ir(III)/Ir(V) catalytic cycle is in operation. The first step involves the oxidative addition of a C–H bond of the substrate to the Ir(III) complex, which is followed by reductive elimination of the borylated product. In the majority of examples of arene borylation, the oxidative addition has been proposed or demonstrated to be the rate- and selectivity-determining step. Our first goal was to revisit this basic mechanism computationally using the sulfonated bipyridine and the two substrates under investigation to ensure that this general mechanistic picture remains consistent in the presence of the outersphere interactions that we envisage are in operation. As the full catalyst system containing the quinine-derived chiral cation is so large, we opted to evaluate the catalytic cycle on a significantly reduced model system using the tetramethylammonium cation to define the likely rate- and selectivity-determining steps. We started with system **I**, consisting of the tris-boryl complex and the standard amide substrate (Figure 2, magenta, also Figure S1 in ESI). In agreement with the accepted mechanism, we located the oxidative addition transition state (TS) **II–III**, the subsequent heptacoordinate Ir(V) complex **III**, the reductive elimination TS **III–IV**, and the resulting iridium hydride–product complex **IV**. Of these steps, the oxidative addition is clearly the rate-determining step with a barrier of 24.8 kcal/mol. This TS incorporates the understanding of energetically favorable features in the oxidative addition TS developed below. The remaining steps regenerate the catalyst through addition of B_2_Pin_2_ (**V–VI**) and elimination of HBPin (**VI–I**). Both have a lower barrier than the oxidative addition and, as they do not contain the substrate, should not impact the reaction selectivity. An analogous study with the phosphinamide substrate was also conducted (Figure 2, cyan). As in the amide system, the oxidative addition step (**II–III**) has a larger barrier than the reductive elimination step (**III–IV**) and therefore is also deemed to be the selectivity-determining step.

### Full System Component Interaction Studies

Having confirmed the oxidative addition of the arene C–H bond to the Ir complex as the enantioselectivity-determining step, we turned our focus to the full system bearing the chiral cation for each substrate in turn. The systems contain the iridium tris-boryl complex, the dihydroquinine-derived cation with the extended benzyl group, and the amide substrate in the first case and phosphinamide substrate in the second (Figure 1d). These systems are exceptionally large for a full-DFT study, containing 263 and 266 atoms, respectively. They are also complicated conformationally, with the cation having several rotatable bonds as well as very high freedom of orientation with respect to the Ir complex. In combination, these factors mean that the full study was likely to require the calculation of a large number of conformers. More importantly, we anticipated the significant challenge of interpreting the energetic differences between the diastereomers and conformers, with the various components of the full system interacting in complex ways. Therefore, we resolved to first take a deconstructive approach, whereby each of the three main components (Ir complex, substrate, and chiral cation) is first examined separately. Following this, we would incrementally build up the complexity by looking at the pairwise interactions of each component.

**Figure 3:**
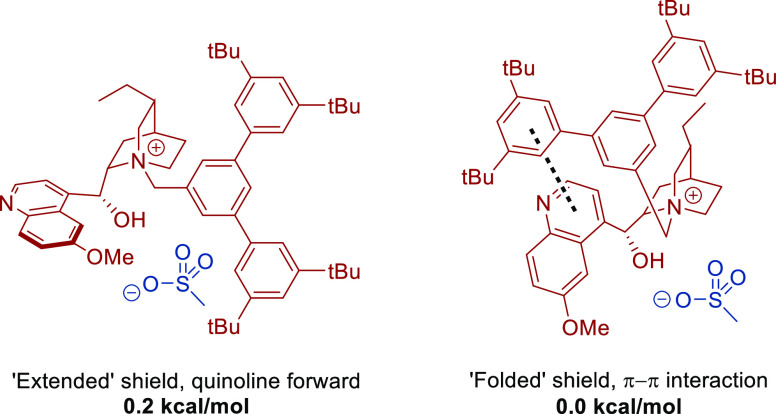
Two most important conformations of the chiral cation.

The conformational space for substrate and the Ir tris-boryl complex is quite simple, but the cation is more complex and, therefore, was explored in greater detail. It was found that the cation has two main energetically similar conformations which we have dubbed “extended” and “folded” (Figure 3). In the extended conformation, the quinoline substituent and the large benzylic “shield” substituent are oriented as far away from each other as possible. Conversely, in the folded conformation, these two substituents are close together and engage in a π–π interaction, stabilizing this sterically more congested conformation. However, the energies of both conformations are within 0.2 kcal/mol of each other, meaning that both are plausible in the active catalytic species. Further conformations were also identified, but all were significantly higher in energy.

**Figure 4:**
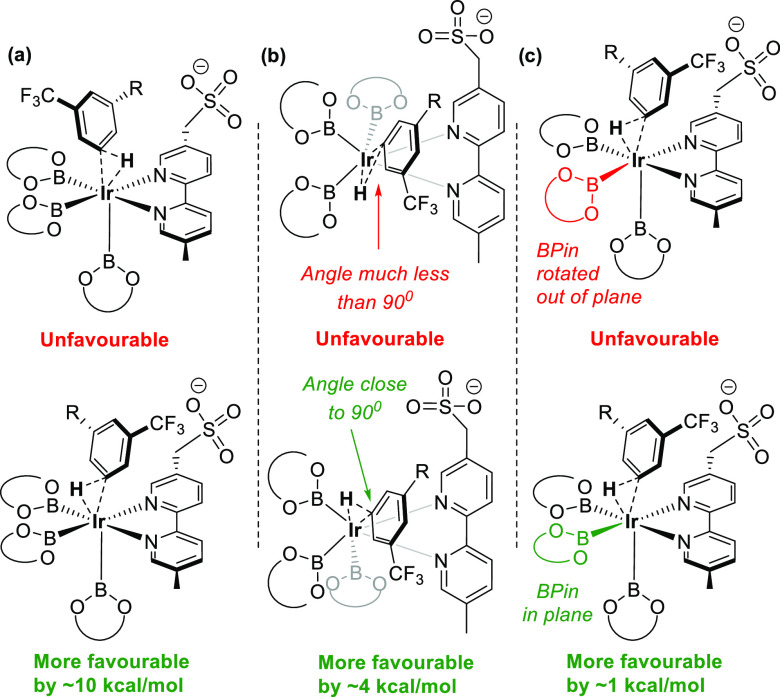
Exploration of the geometric preferences of the oxidative addition transition states in the absence of the chiral cation. Me_4_N^+^ cation was included in computations but omitted here for clarity. (a) Direction of H migration (b) angle of H migration trajectory (c) angle of BP in substituent.

We then proceeded to investigate the pairwise interactions between the three key components. We first examined oxidative addition of the substrate to the Ir complex using tetramethylammonium as a surrogate for the chiral cation (Figure 4). Three important features related to the geometry around the Ir center were identified. First, hydrogen atom migration occurs much more favorably toward a boryl ligand than toward a bipy ligand by about 10 kcal/mol, a preference that has been noted in previous computational studies (Figure 4a).^27a^

Second, hydrogen atom migration preferentially occurs toward the boryl substituent that is closest to 90° out of the substrate aryl ring plane (Figure 4b). Finally, it is generally not possible for both equatorial BPin ligands to be fully aligned with the main plane of the Ir–bipyridine complex—one must be twisted out of the plane because of steric congestion. Oxidative addition is found to be preferred toward an in-plane boryl ligand compared with an out-of-plane ligand by about 1 kcal/mol (Figure 4c). Ultimately, all of these observations can be rationalized by the existence of favorable orbital interactions between the migrating hydrogen atom and the boryl ligands in the TS. While these constraints are undoubtedly significant in isolation, it is important to consider that the substrates will have their own conformational preferences. Similarly, hydrogen bonding between the amide/phosphinamide and the sulfonate group is also an energetically important interaction and will affect the substrate positioning in the active site.

**Figure 5:**
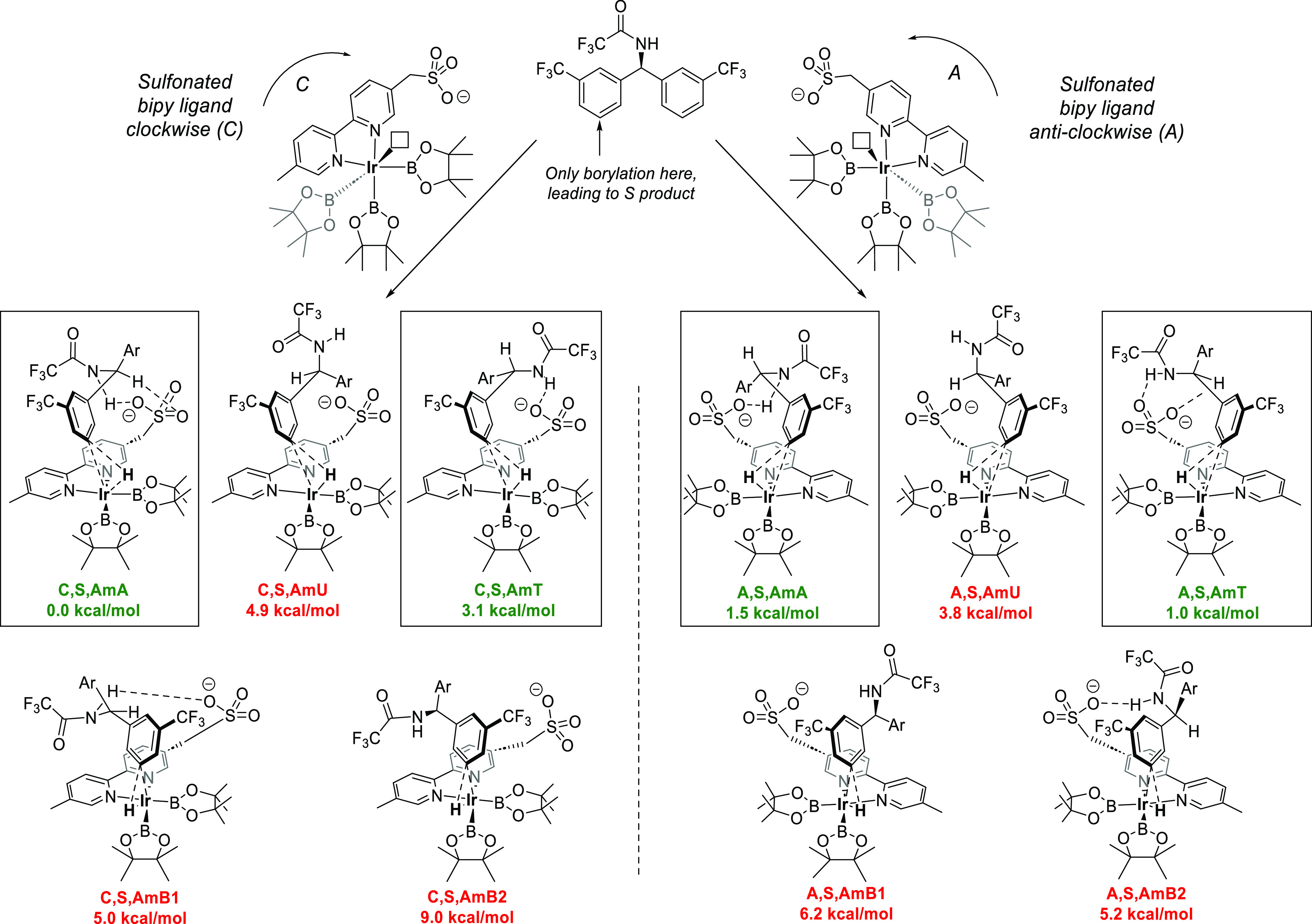
Exploration of amide oxidative addition TS conformers with a simplified Me_4_N^+^ cation (not shown for clarity). Axial boryl ligands not shown for clarity.

To fully understand the interplay of these various constraints, an exhaustive model study of possible oxidative addition TSs was undertaken with the achiral Me_4_N^+^ cation in place of the dihydroquinine-derived chiral cation (Figure 5). The Ir complex can be viewed as having two possible configurations: with the anionic bipyridine ligand **L** oriented either clockwise (C) or anticlockwise (A) with respect to the plane of the Ir–bipyridine complex when the vacant coordination site is pointing up. The use of an achiral cation obviated the need to explore both enantiomeric pathways, and only the study of *S*-product forming pathways was arbitrarily chosen. With a defined product absolute configuration, this gives rise to two possible diastereomeric families of TSs (left side of figure clockwise, right side of figure anticlockwise). Within these families, the hydrogen atom can migrate toward either the boryl ligand closer to the sulfonate (e.g., **C,S,AmA**, **C,S,AmU**, **C,S,AmT**) or the more distant one (**C,S,AmB1** and **C,S,AmB2**). Overall, the former transition states, with hydrogen migration toward the proximal boryl ligand, were more favorable because in most cases the key amide–sulfonate hydrogen bond can be retained. Further conformational variation is possible through rotation of the substrate benzylic single bond, with the amide oriented away from the sulfonate (**AmA**), up (**AmU**), or toward the sulfonate (**AmT**). **AmA** and **AmT** conformations both allow hydrogen bonding to occur between the amide and the sulfonate and are, hence, most favored. Taken together, this meant that four competing TSs, **C,S,AmA**, **C,S,AmT**, **A,S,AmA**, and **A,S,AmT**, all had relative free energies within ∼3 kcal/mol of each other and so were selected for a subsequent full system study. Intriguingly, in the two lowest-energy TSs, **C,S,AmA** and **A,S,AmT**, an additional weaker hydrogen bond between the sulfonate and the doubly benzylic hydrogen in the substrate was also observed.

**Figure 6:**
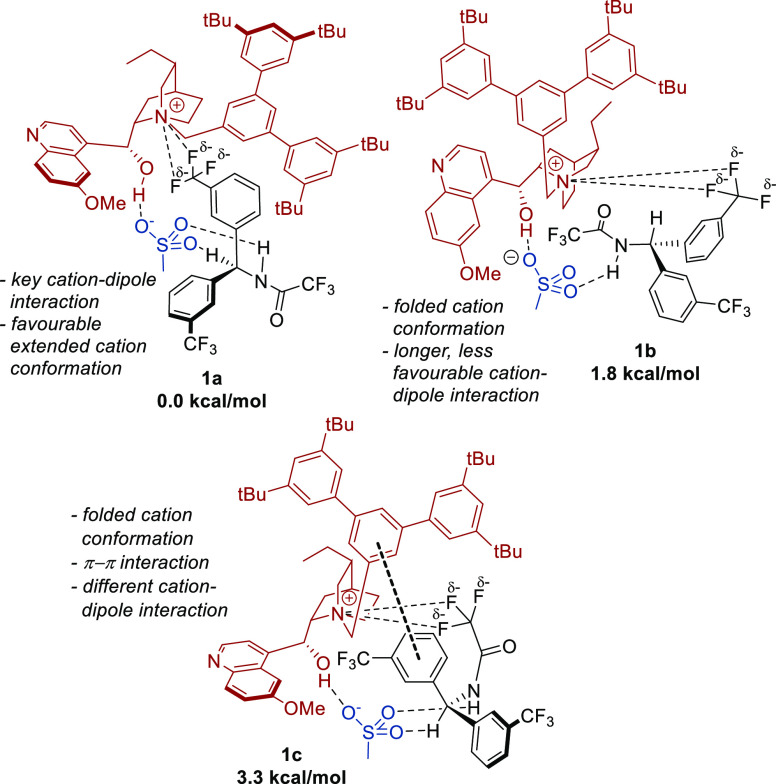
Three lowest-energy cation–substrate model system conformers, showcasing the diversity of noncovalent interactions available to the system.

Having thoroughly explored the complex–substrate interaction, we turned our attention to exploring possible substrate–cation noncovalent interactions (NCIs), now in the absence of the Ir complex (Figure 6). It was almost immediately apparent that the sulfonate group played a key organizational role in assembling the three components. For this reason, a mesylate anion was included in these model systems to retain this central functionality around which we envisaged the three components would be arranged. In the lowest-energy conformer **1a** of the model system, both the amide NH and the benzylic proton form hydrogen bonds with the sulfonate group of the mesylate. In addition, a hydrogen bond between the hydroxyl group on the cation and sulfonate was also present. An important and unanticipated additional NCI was found to be a cation–dipole interaction between the quaternary ammonium functionality of the cation and a trifluoromethyl substituent on one of the aromatic rings of the substrate; **1b**, with a folded cation conformation and a longer cation–dipole interaction, was 1.8 kcal/mol higher in energy. Conformers containing other additional NCIs were identified, including **1c**, displaying a π–π interaction between the substrate and the central aromatic ring of the benzylic “shield”, but this was significantly higher in energy (3.3 kcal/mol). Finally, various plausible interactions between the cation and the full Ir complex were also investigated but did not reveal any important NCIs besides the anticipated hydrogen bond between the ligand sulfonate and the hydroxyl group of the cation.

### Amide Borylation Enantioselectivity Studies

**Figure 7:**
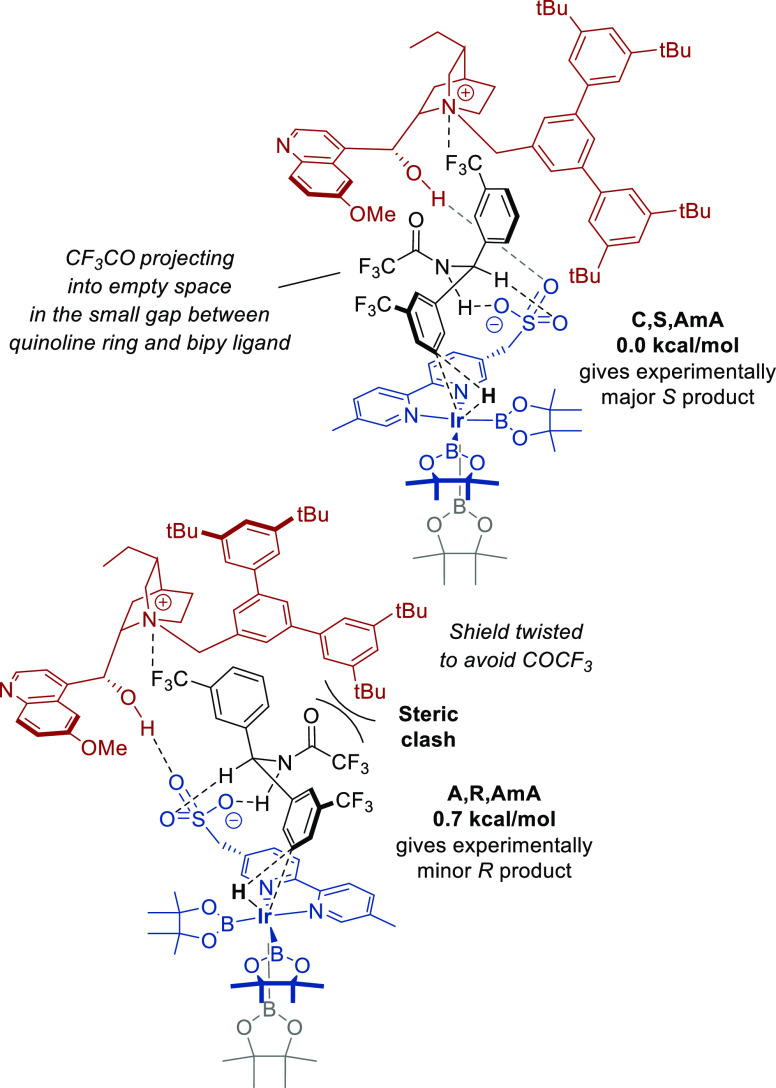
Lowest-energy transition states leading to the experimentally major and minor amide product enantiomers.

**Scheme 1:**
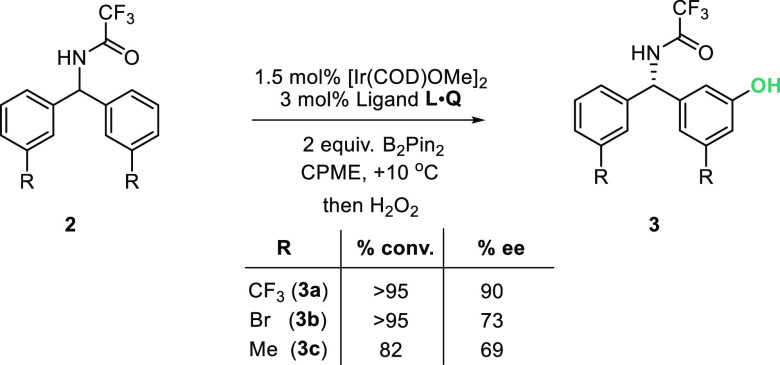
Direct Comparison of Enantioselectivity with Different Substituents at the *Meta*-Position of Substrate

Confident that we had developed a sufficient understanding of the important pairwise interactions between the three components, we could now use it to inform a thorough conformational exploration of the full amide substrate system. This was initially done using molecular mechanics with an OPLS3 force field, running a separate conformational search for each of the **AmA** and **AmT** prototypes in Figure 5, but this time including the full chiral cation. Transition states leading to the minor *R* product enantiomer were also investigated in the same manner. Carefully selected TS conformations were then optimized with full DFT on all 263 atoms, leading to 81 gas-phase transition states. The 12 TSs with the lowest gas-phase free energies were then further reoptimized in solvent (diethyl ether) using the SMD solvent model. We were pleased to find that the lowest-energy solvent-optimized TSs leading to each product enantiomer had all of the interactions previously identified as being most favorable during the model studies (Figure 7). The two lowest-energy TSs leading to the two enantiomeric products **C,S,AmA** (*S*, major enantiomer) and **A,R,AmA** (*R*, minor enantiomer) both featured two hydrogen bonds between the substrate and the sulfonate group on the ligand. In both, the hydrogen migration takes place in the direction of the boryl ligand proximal to the sulfonate, maximizing the favorable orbital interactions and retaining hydrogen bonding between the ligand and substrate during this process. A hydrogen bond between the cation OH and the sulfonate was also present, contributing to the high level of organization at the TS.^29^ Finally, both TSs featured the attractive cation–dipole interaction between the quaternary ammonium and the trifluoromethyl substituent on the aromatic ring of the substrate—this was also apparent in the NCI plots (see ESI, Figure S3). This insight is consistent with the experimental observation that the substrates giving the highest enantioselectivity generally feature electronegative substituents at the meta-position of the aryl rings. To probe this, enantioselectivity in the borylation of three substrates with electronically varied substituents at the arene meta-position (CF_3_, Br, and Me) was compared at +10 °C (Scheme 1). This is a higher temperature than the −10 °C found optimal in our original report but was necessary to obtain reactivity for methyl-substituted substrate **3** and enable direct comparison of the three. This comparison showed a clear decrease in enantioselectivity as R became less electronegative, supporting the importance of the cation–dipole interaction with the substrate.

The calculated energy difference (0.7 kcal/mol) between the two diastereomeric transition states is somewhat lower than what the observed enantioselectivity (90% ee) would suggest. A limited quantitative agreement is not unexpected for a system this challenging, and similar enantioselectivity results have been observed in other large systems.^30^ To see if the agreement could be improved further by changing the computational method, an additional benchmark study was conducted (see ESI, Table S1). We tested inclusion of dispersion corrections in the geometry optimization (r2SCAN-3c), as well as wB97M-V functional for the single-point energy calculations. The dominant interactions and the overall TS energy ranking were robust to these changes in the computational methods. However, both modifications reduced agreement with the experimentally observed enantioselectivity, predicting either effectively no or opposite selectivity. Therefore, we adhered to the existing computational workflow for the remainder of this study. Despite only providing a qualitative match with the experiment, the two calculated transition states allow us to suggest an enantioselectivity model for this complex reaction. All of the attractive interactions present in the lowest *S*-forming TS are also present in the lowest *R*-forming TS. However, in the former, the trifluoroacetamide group is projecting into the open space in a small gap between the sulfonate group of the ligand and the quinoline ring of the cation, experiencing little steric impediment (Figure 7, upper). In the *R*-forming transition state, the trifluoroacetamide group is clashing with the large quaternizing “shield” group of the cation, thereby causing it to rotate into a less favorable and higher-energy conformation, providing a rationalization for the observed selectivity (Figure 7, lower).

**Figure 8:**
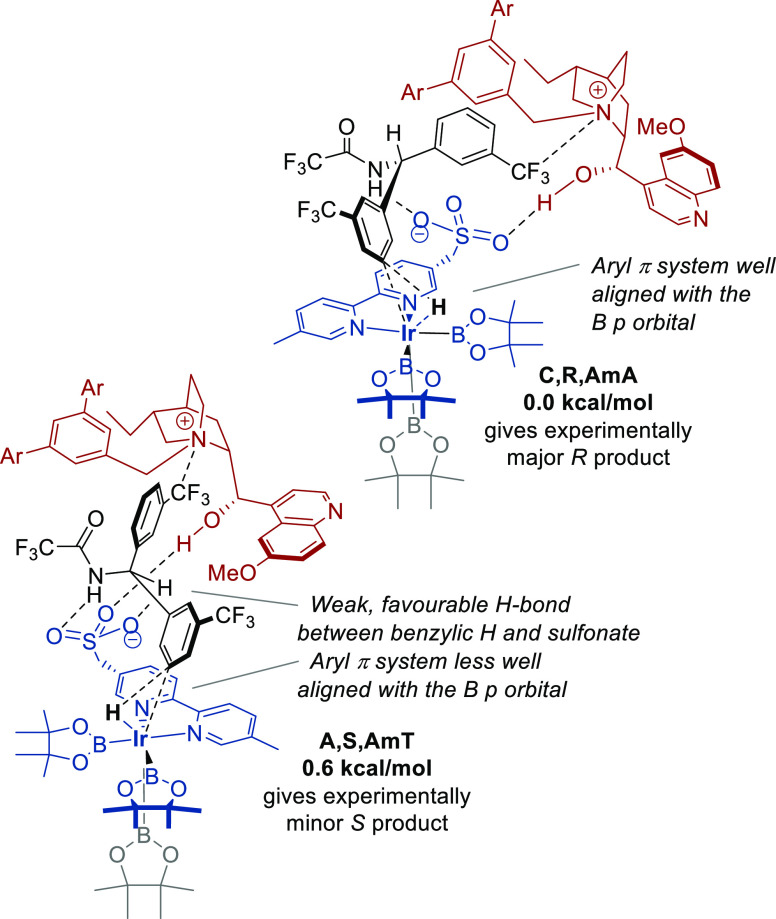
Lowest-energy transition states leading to the experimentally major and minor amide product enantiomers, when using the pseudoenantiomeric **QD** cation.

**Scheme 2:**
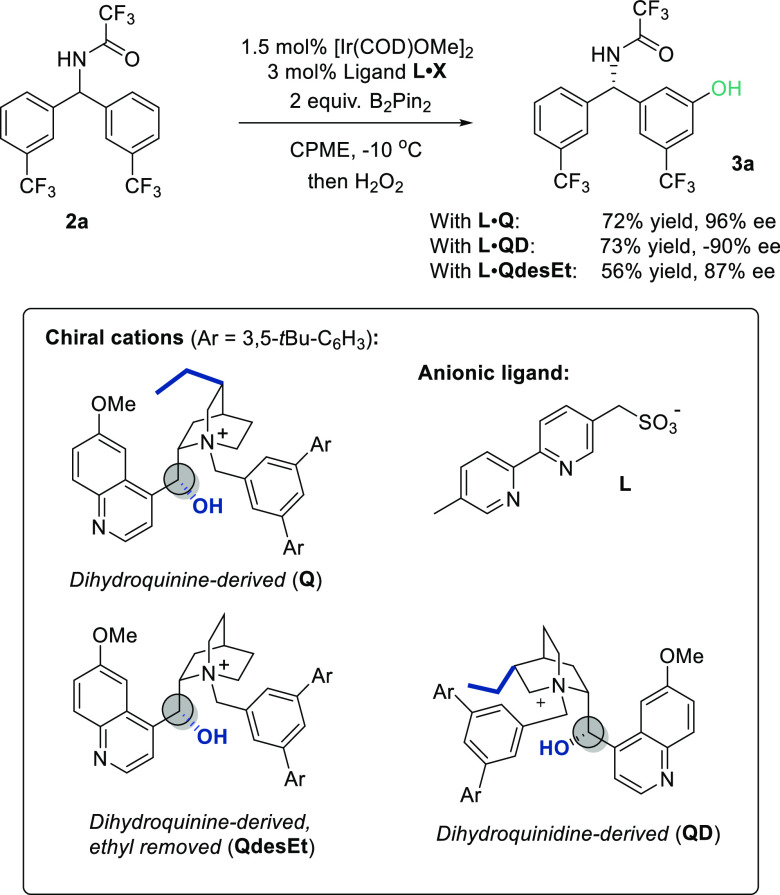
Investigations of Pseudoenantiomeric and De-ethylated Chiral Cations for the Amides

In our original study, we had observed that use of the pseudoenantiomeric dihydroquinidine-derived cation **QD** gave a product with very similar enantioselectivity to that obtained when using the dihydroquinine-derived cation **Q**, but with opposite absolute stereochemistry (Scheme 2, **L·Q** vs **L·QD**). Following a similar workflow, we then investigated the **QD-**based catalyst system from scratch, reasoning that computationally this would serve as a good test of robustness of the approach. Similar conformational searches and DFT gas-phase TS optimization yielded 79 gas-phase TSs. Of these, 16 were optimized in solvent. Interestingly, the previously lowest **C,S,AmA** and **A,R,AmA** were not the lowest with this cation and had very similar energies—0.6 and 0.9 kcal/mol relative to the lowest TS, respectively. Instead, diastereomers of these, **C,R,AmA** and **A,S,AmT**, had lower energies and still featured the important cation–dipole interaction (Figure 8). The energy difference between these TSs (0.6 kcal/mol) is also qualitatively consistent with the observed enantioselectivites. In this case, the most important factor in the energy difference between the enantiomeric TSs appears to be the alignment of the aryl ring relative to the boryl ligand. The B–Ir–C–C dihedral angle in **C,R,AmA** was 71° but only 51° in **A,S,AmT**. Our model studies showed that aryl ring orientation as close to 90° as possible is the most favorable; therefore, this indicates an inferior interaction between the aryl ring π system and the boron *p* orbital in the **A,S,AmT** TS. As was deduced from the model studies (Figure 4), variation of this dihedral angle can result in energy differences of up to 4 kcal/mol. The underlying cause for the different lowest-energy TS in this system is not immediately clear. However, the very close spacing of all four TSs (all within 1 kcal/mol) clearly illustrates the complex conformational landscape of these complexes and the challenges they present in computational investigations.

The key difference between the **Q-** and **QD**-derived cations that makes them diastereomers rather than enantiomers is the positioning of the ethyl group on the quinuclidine portion. The fact that both gave very similar enantioselectivity (in opposite directions) suggests that the ethyl group is playing no role and indeed examination of the transition states shows that this ethyl is pointing into free space. This led us to hypothesize that a cation in which the ethyl group has been removed should give a very similar outcome. We performed this experiment using a cation derived from de-ethylation of dihydroquinine and indeed found the enantiomeric excess to be extremely similar, as predicted (Scheme 2, **L·Q** vs **L·QdesEt**).

**Scheme 3:**
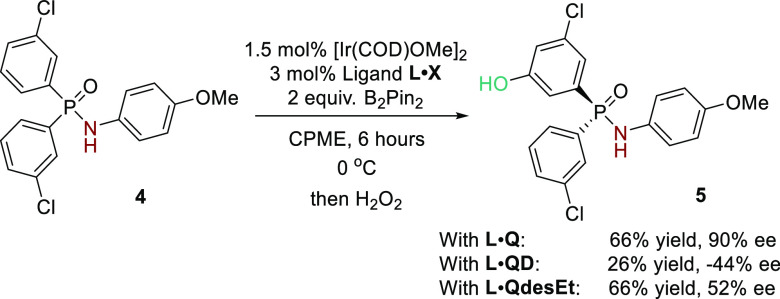
Investigations of Pseudoenantiomeric and De-ethylated Chiral Cations for the Phosphinamides

Having obtained insights into the crucial interactions at play in the amide system as well as to the factors potentially important for the enantioselectivity, we were keen to follow a similar procedure to investigate the other class of substrates from our original report, the phosphinamides. This is an important substrate class as the enantioselective borylation gives rise to enantioenriched chiral-at-phosphorus compounds which are challenging to obtain by other means. While the exact catalyst that was optimal for the amides translated very effectively to the phosphinamides, subsequent investigations have revealed tangible and intriguing differences between the two systems. Specifically, and in contrast to the amide substrates, we have subsequently found that for the phosphinamides the enantiocontrol using the QD-derived pseudoenantiomer is inferior, giving only −44% ee (Scheme 3, **L**·**Q** vs **L**·**QD**). The origins of this difference are not obvious but would broadly suggest that different interactions may be occurring at the transition state. We have now investigated the removal of the ethyl in the dihydroquinine series (**QdesEt**) and found this to be detrimental compared with the parent dihydroquinine-derived cation (Scheme 3, **L·Q** vs **L·QdesEt**). Taken together, these observations are intriguing in that they suggest that the presence of the ethyl in the optimal dihydroquinine-derived cation (**Q**) is actively aiding enantioinduction in the phosphinamide series, while it has little effect in the amide series, suggesting a quite different picture at the enantiodetermining transition state.

**Figure 9:**
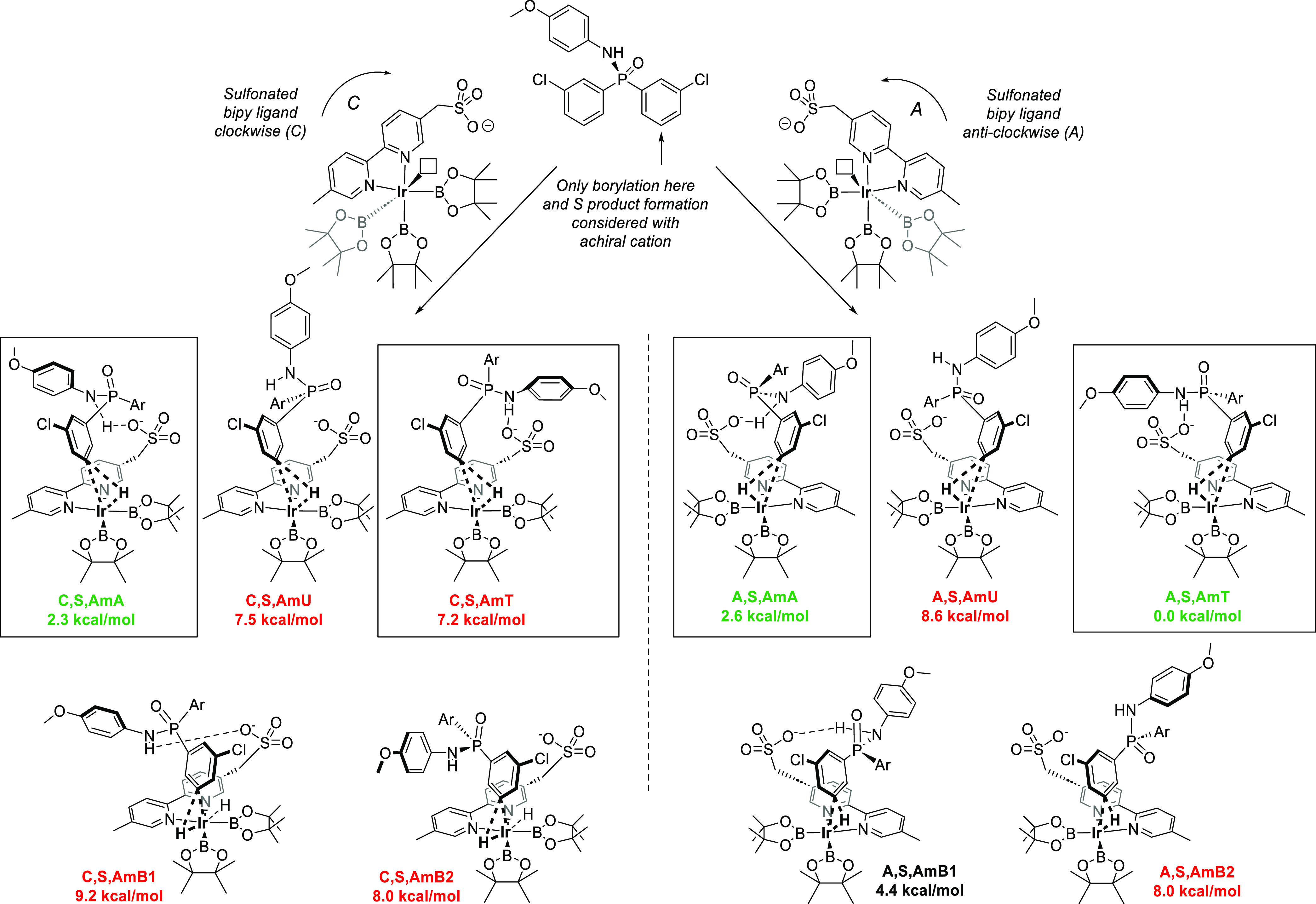
Exploration of phosphinamide oxidative addition TS conformers with a simplified Me_4_N^+^ cation (not shown for clarity). Axial boryl ligands are not shown for clarity.

The phosphinamide substrates were examined following a similar computational approach that had been followed for the amides. An exhaustive model TS set was first optimized, using the achiral Me_4_N^+^ cation instead of the large dihydroquinine-derived cation (Figure 9). Both clockwise (C) and anticlockwise (A) Ir complex isomers were considered. As in amide studies, because of the achiral cation, there was no need to explore both enantiomeric pathways, with the study of *S* pathways being arbitrarily chosen. Conformers arising from the rotation along the C–P single bond and their possibility of forming the key phosphinamide-sulfonate hydrogen bond were explored. Similar to amides, three TSs featuring this hydrogen bond were particularly low in energy—**C,S,AmA**, **A,S,AmA**, and **A,S,AmT. C,S,AmT**, while higher in energy, was also included in further investigations for completeness.

**Figure 10:**
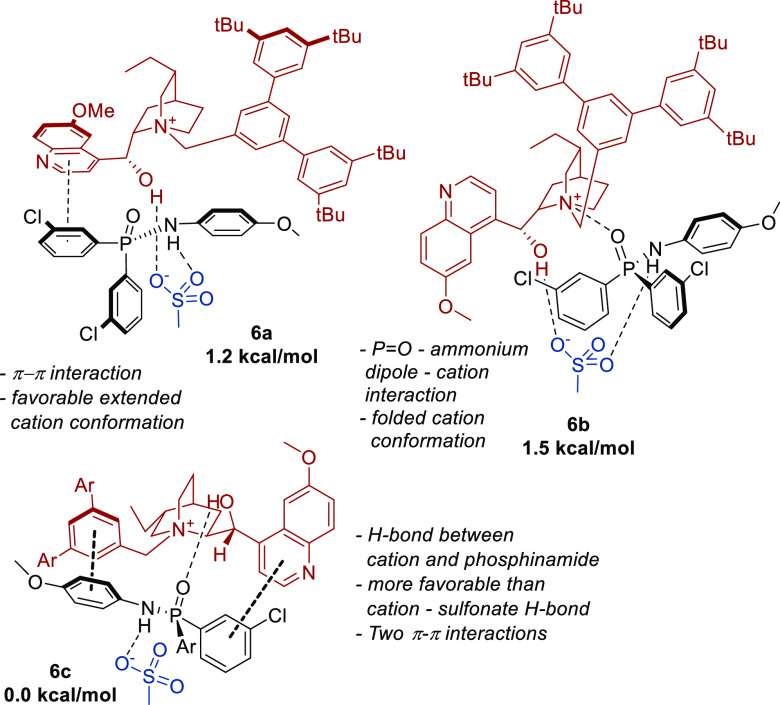
Most favorable noncovalent interactions between the chiral cation and the phosphinamide substrate.

To understand the noncovalent interactions between the phosphinamide substrates and the cations in the absence of the Ir complex, a model study focusing on these two components was next conducted (Figure 10). In contrast with the amide substrates, it became apparent that the phosphinamides had a propensity to engage in π–π interactions with the cation’s quinoline ring (conformer **6a**). Cation–dipole interactions between quaternary ammonium and phosphinamide oxygen were also favorable (conformer **6b**). Furthermore, TSs featuring the cation–dipole interactions favored by the amide substrates were significantly higher in energy in this phosphinamide system (by >7 kcal/mol). These model studies initially assumed that the cation hydroxy group would always form a hydrogen bond to the sulfonate on the ligand, as was always the case in the amide studies. However, full system conformational searches identified several low-energy conformations with the cation hydroxyl group forming a hydrogen bond with the phosphinamide oxygen instead. This prompted us to investigate this interaction in the model system (conformer **6c**) and we found that this interaction was the energetically most favorable. Two π–π interactions between the cation and the substrate were also found to be present. Additionally, while the phosphinamide phosphorus atom is highly polarized with a Mulliken atomic charge of +0.6, no evidence of it outcompeting the ammonium cation in binding with the sulfonate anion was seen with the full cation or in previous investigations with the model Me_4_N^+^ cation.

### Phosphinamide Borylation Enantioselectivity Studies

The full catalyst system with the dihydroquinine-derived cation was then investigated, first by running thorough conformational searches for the four possible diastereomeric systems (**C,S**, **A,S**, **C,R**, and **A,R**). Key conformations were then carefully selected and optimized in the gas phase. The lowest-energy TSs featured a hydrogen bond between the cation’s hydroxyl group and the phosphinamide, in contrast to the amide system, where the hydroxyl prefers to interact with the sulfonate group on the ligand. This discovery required further detailed conformational exploration.

Another surprise was that in some lower-energy TS conformations, the H atom migration toward the boryl ligand distal from the sulfonate was more energetically favorable. This is another key difference from the amide system and again prompted further exploration of all TS diastereomers. In total, the conformational exploration yielded 135 fully DFT-optimized gas-phase TS geometries of this 266-atom system, which was a significant undertaking.

**Figure 11:**
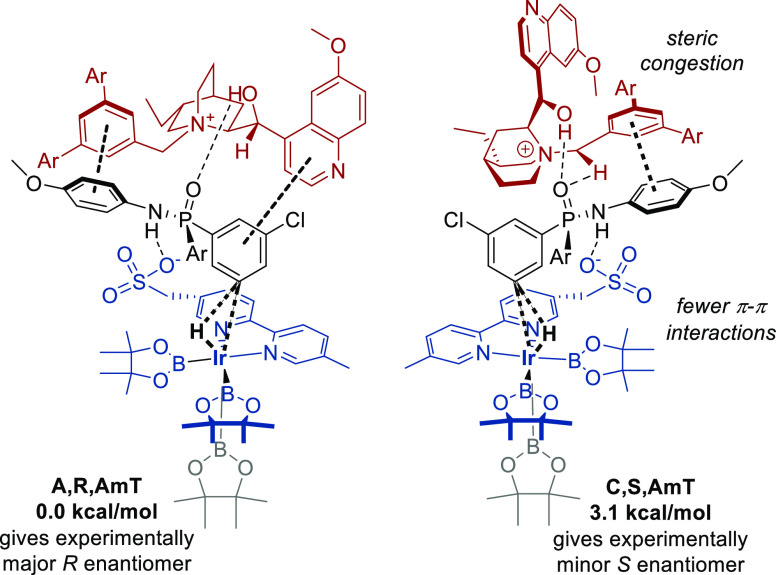
Lowest-energy transition states leading to the experimentally major and minor phosphinamide product enantiomers using the **Q** cation.

A smaller selection of 12 lowest-energy TSs was then reoptimized using the SMD(diethyl ether) solvent model. The lowest-energy TSs for each product enantiomer are shown in Figure 11. The **A,R,AmT** TS is calculated to have an activation free energy of 3.1 kcal/mol lower than that of the enantiomeric **C,S,AmT**, thus again qualitatively agreeing with the experimentally observed enantioselectivity. The limited quantitative agreement is likely due to the previously discussed challenges of size, conformational freedom, and complex mixture of various noncovalent interactions. Both TSs feature H atom migration toward the distal boryl ligand, as well as a hydrogen bond between the catalyst OH and the phosphinamide oxygen atom. Clear π–π interactions are present in both diastereomeric TSs. The key difference between the two TSs is that in the lower-energy **A,R,AmT** TS the cation is in a favorable extended conformation, while in the higher-energy **C,S,AmT**, it has to adopt a less favored folded conformation to preserve the noncovalent interactions with the other components. Also, the extended conformation of **A,R,AmT** allows for two π–π interactions, while only one π–π interaction is possible in the higher-energy **C,S,AmT** transition state. These were also confirmed in the NCI plots (ESI, Figure S5).

These TSs also hint at a possible explanation for the markedly different effect of the catalyst ethyl group removal in the phosphinamide and amide systems. The des-ethylated cations performed well on amide substrates, but in phosphinamide systems, a significant drop from 90% ee to 55% ee was observed (Scheme 3). These experimental data suggest that the ethyl group in the quinine-derived cation plays an active role in increasing the enantioselectivity for this substrate class. While we do not have a full explanation for this effect, it is likely to be linked to cation conformations in each of the systems. With an amide substrate, the extended cation conformations are favored for both major and minor enantiomeric TSs (Figures 7 and 8). However, with the phosphinamide substrate, the minor enantiomeric TS favors a folded conformation, and it is possible that the ethyl group has some increased influence here. Using a similar approach, the system with a pseudoenantiomeric dihydroquinidine-derived cation **QD** was also investigated, which correctly predicted for the product to have the opposite enantioselectivity, matching the experimental observations (see ESI, Figure S2).

**Scheme 4:**
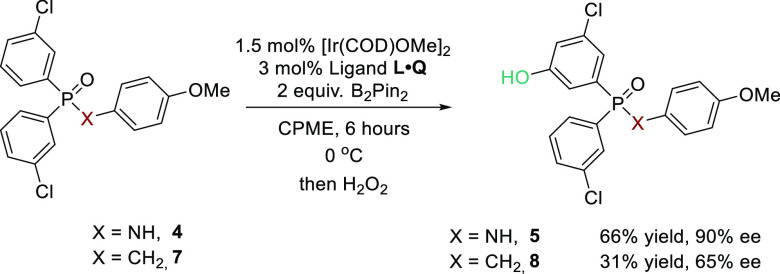
Replacement of NH with CH_2_ in the Phosphinamide Substrate: Borylation of a Phosphine Oxide

A key difference that is evident when comparing the calculated transition states for the amides and phosphinamides is that for the latter our calculations predict significantly more direct interactions between the chiral cation and the substrate: a hydrogen bond between the phosphoryl oxygen and the cation hydroxyl as well as two distinct π–π interactions (Figure 11). This is in stark contrast to the amides, where the cation–dipole interaction was the only direct cation–substrate interaction identified (Figure 7). On this basis, we predicted that replacement of the phosphinamide NH with a methylene unit, although severing the hydrogen bond between substrate and ligand, may still allow appreciable enantioselectivity to be obtained due to the significant number of interactions that can still be occurring directly with the cation, allowing substantial organization to be maintained. To test this, we synthesized the benzyl phosphine oxide substrate **7** and subjected it to the optimal borylation conditions (Scheme 4). As can be seen, although selectivity was reduced, it remained very respectable, giving the product **8** in 65% ee and providing support to our hypothesis. While it is possible that phosphine oxide has a different binding mode, overall this exciting result fits well with our new computational understanding of this system and gives us encouragement that we will be able to develop future systems in which the substrate does not necessarily have to contain a hydrogen bond donor.

## Conclusions

**Figure 12:**
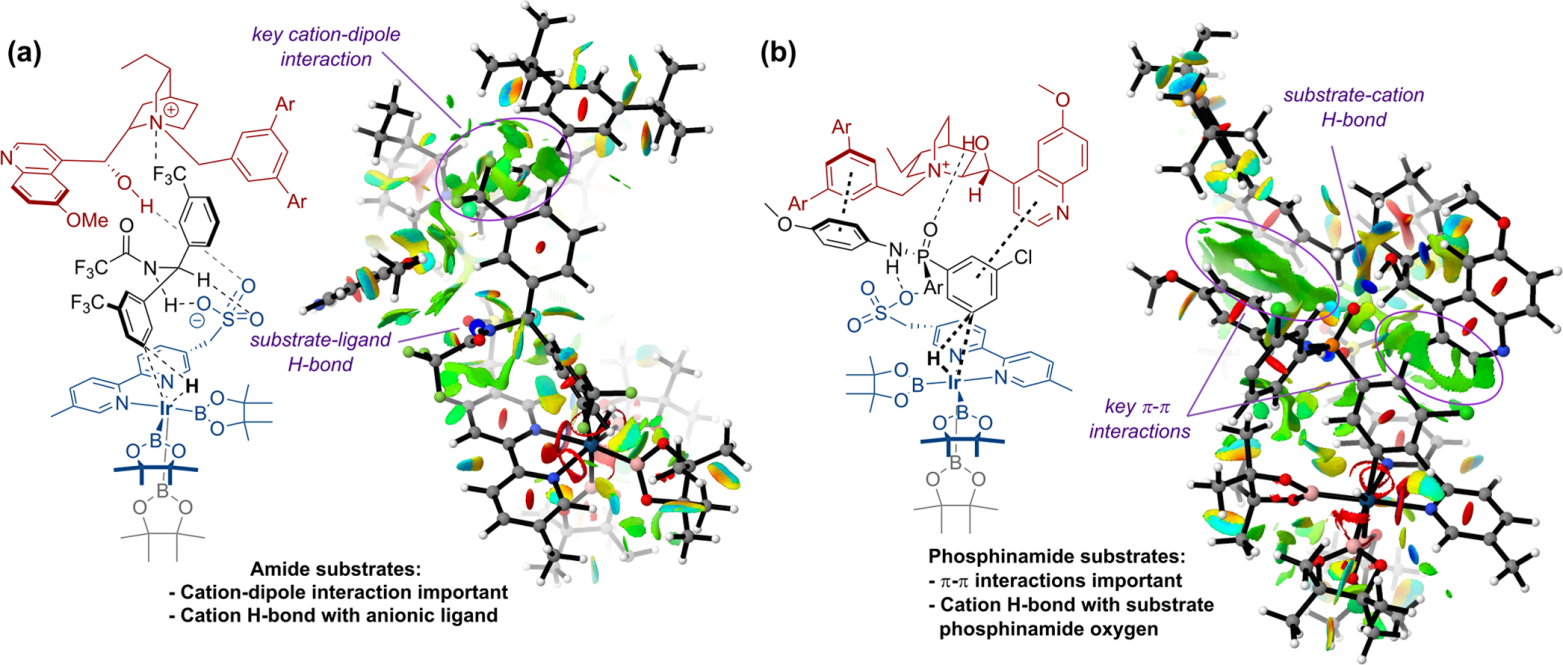
Comparison of substrate–catalyst interactions in amide (a) and phosphinamide (b) substrates using the same quinine-derived cation Q.

The computational analysis of this unusual and novel catalytic system presented several challenges, including very large system sizes, the significant conformational freedom in the catalytic complex, and a diverse array of possible noncovalent interactions. To develop a thorough understanding of these systems, more than 420 gas-phase transition states of the full 260-atom systems were optimized. This combination of system size and depth of investigation has only limited precedent in the literature.^15^ As a result of this detailed study, we were able to qualitatively computationally predict the absolute sense of enantioselectivity in four different challenging systems. More importantly, we were able to decipher the overall organization of the active catalyst and the key noncovalent interactions between the substrates and the chiral cation. Specifically, it was found that amides and phosphinamides are bound in the catalyst pocket in very different ways (Figure 12). Amides form a hydrogen bond with the sulfonate on the anionic bipyridine ligand and a cation–dipole interaction between the electronegative substituent on the substrate aryl ring and the quaternary nitrogen of the chiral cation. Phosphinamides also form a hydrogen bond with the sulfonate ligand, but in stark contrast, they also form a hydrogen bond and two π–π interactions with the chiral cation.

Ultimately, we believe that these differences arise from the distinct nature of each substrate class. The phosphinamide has three aromatic fragments and can adopt a flatter geometry, thus making π–π interactions with the chiral cation more favorable. In contrast, the amide substrates possess one less aromatic substituent and are strictly tetrahedral; therefore, π–π interactions are less favorable, and the cation–dipole interaction with the ArCF_3_ is the dominant interaction with the chiral cation. The differences in hydrogen bonding can be explained analogously. The trifluoroacetamide group of the amide substrates has a flat geometry and can form a hydrogen bond with either the sulfonate or the cation but not both. This is not true for phosphinamides, where both NH and O can be inclined away from the Ir complex and form two hydrogen bonds at the same time. Further support for these differences in substrate binding was gained from various experimental observations and NCI plots. While the enantioselectivity prediction results should be treated with some caution, we are confident in the elucidated sets of most favorable noncovalent interactions for each substrate class as the alternative binding modes were all 1.5–2.5 kcal/mol higher.

The findings of this study are important, because they allow us to gain insight into the nature of the controlling interactions. The cinchona alkaloid-derived cations possess remarkable and versatile structural features but going forward it is important to have some idea of feasible interactions for a particular substrate, even if a *de novo* system design is still some distance away. Furthermore, the insights gained from the calculations guided us to explore a prochiral phosphine oxide substrate that bears no hydrogen bond donor functionality. The encouraging levels of enantioselectivity obtained suggest that it will not always be necessary to have an explicit direct interaction of the substrate with the ligand if sufficient interactions with the cation can be formed. This is highly encouraging, in terms of expanding the future scope of this methodology. We predict that the findings obtained in this study will be of great use to ourselves and others, who may seek to apply chiral cations as controllers in other metal-catalyzed reactions, a strategy that has much potential for addressing unsolved problems in enantioselective transition-metal catalysis.

## Supporting Information Available

Full experimental details and characterization data for compounds, computational methods, additional NCI plots, and a description of the associated computational data set are available in the electronic Supporting Information. Full set of computational 3D structures and DFT output files are available as a data set in University of Nottingham repository at 10.17639/nott.7218 (PDF)
