# Supplementary material for: Interrogating the Crucial Interactions at Play in the Chiral Cation-Directed Enantioselective Borylation of Arenes
Source: ACS Catal. 2023 Sep 22;13(19):13043–55. doi: 10.1021/acscatal.3c03384 (PMC10563137; doi:10.1021/acscatal.3c03384)
Supplement: Supplementary file 1 — cs3c03384_si_001.pdf [file cs3c03384_si_001.pdf]

Interrogating the Crucial Interactions at Play in the Chiral Cation-Directed Enantioselective Borylation of Arenes

Kristaps Ermanis*,[a] David C. Gibson,[b] Georgi R. Genov [b] and Robert J. Phipps*,[b]

[a] School of Chemistry, University of Nottingham, University Park Nottingham, NG7 2RD, United Kingdom; Email: kristaps.ermanis@nottingham.ac.uk

[b] Yusuf Hamied Department of Chemistry, University of Cambridge, Lensfield Road, Cambridge, CB2 1EW, United Kingdom; Email: rjp71@cam.ac.uk

Table of Contents

General Information S3
Experiments which suggest against cation borylation occurring in situ S4
Catalyst Synthesis S4
Synthesis of starting materials S6
Enantioselective borylation procedures S7
SFC and HPLC Traces S11
NMR Spectra S15
Computational Investigations S32
  Computational methods S32
  Additional Computational Figures S33
  Dispersion-corrected geometry optimisation benchmark S35
  3D geometry depictions and non-covalent interaction plots for lowest energy full system transition states S37
  Summary of the associated computational dataset contents S41
References S72

General Information

Reagents: All reagents, unless otherwise stated, were used as supplied from commercial sources without further purification. $[Ir(COD)OMe]_2$ was purchased from Sigma-Aldrich, used as received and stored in a desiccator. Et_2O, CH_2Cl_2, THF, methanol, CPME, *n*-hexane, acetonitrile and toluene were purified by distillation on site under inert atmosphere *via* the following processes: THF and Et_2O were pre-dried over sodium wire then distilled from calcium hydride and lithium aluminium hydride. CH_2Cl_2, methanol, *n*-hexane, acetonitrile and toluene were distilled from calcium hydride. CPME was distilled over potassium metal.

Reaction setup: Borylation reactions were carried out in 5 mL, 20x84mm crimp-top microwave vials, which were purged with Argon. In cases where the reactions were heated, the vials were heated in deep-welled heating blocks (IKA DB 5.2). In cases where the reactions were cooled, the vials were placed into a Polar Bear Cub^{TM} (by Cambridge Reactor Design) featuring a similar deep-welled block.

NMR spectra: ^1H NMR spectra were recorded on a 600 MHz Bruker Avance DRX-600 spectrometer, 500 MHz Bruker DCH Cryoprobe, 400 MHz Bruker QNP Cryoprobe or 400 MHz Bruker Avance NEO Prodigy N2 Cryoprobe. Chemical shifts are reported in parts per million (ppm) and the spectra are calibrated to the resonance resulting from incomplete deuteration of the solvent ($CDCl_3$: 7.26 ppm; MeOD-d_4: 3.31 ppm, qn). ^{13}C NMR spectra were recorded on the same spectrometers with complete proton decoupling. Chemical shifts are reported in ppm with the solvent resonance as the internal standard ($^{13}CDCl_3$: 77.16 ppm, t; MeOD-d_4: 49.00 ppm, sept). Data are reported as follows: chemical shift δ/ppm, integration (^1H only), multiplicity (s = singlet, d = doublet, t = triplet, q = quartet, qn = quintet, sept = septet, br = broad, m = multiplet or combinations thereof; ^{13}C, ^{19}F and ^{31}P signals are singlets unless otherwise stated), coupling constants *J* in Hz. The carbon atom attached to boron was generally not observed by ^{13}C spectroscopy due to quadrupolar relaxation. ^{19}F and ^{31}P NMR spectra were recorded on a 400 MHz Bruker Avance III HD and 400 MHz Bruker Avance NEO Prodigy N2 Cryoprobe Spectrometer with complete proton decoupling.

High Resolution Mass Spectrometry (HRMS): Some were recorded on a Waters Micromass LCT Premier spectrometer using an electrospray ionization (ESI) or on a Waters Xevo G2-S bench top QTOF using an electrospray ionization (ESI) or atmospheric solids analysis probe (ASAP). Measured values are reported to 4 decimal places are within ±5 ppm of the calculated value. The calculated values are based on the most abundant isotope.

Chromatography: Analytical thin layer chromatography was performed using precoated Merck glass backed silica gel plates (Silicagel 60 F254). Visualisation was by ultraviolet fluorescence (λ = 254 or 365 nm) and/or staining with cerium ammonium molybdate (CAM), potassium permanganate ($KMnO_4$), para-anisaldehyde or vanillin. Flash column chromatography was performed using silica gel 60 (0.040-0.063 µm) from Material Harvest Ltd.

Optical rotations: Measured in $CHCl_3$ or MeOH on a Perkin Elmer 343 Polarimeter using a sodium lamp (λ 589 nm, D-line). $\alpha_D$values are reported at a given temperature (°C) in degrees.cm^2.g^{-1} with concentration in cg.mL^{-1}.

Chiral HPLC analysis: Performed on a Shimadzu XR-LC system with DAICEL CHIRALPAK AD-H column (4.6x250 mm, 5 µm) in a mixed solvent system of ^{n}hexane and ^{i}PrOH.

Chiral SFC analysis: Performed on a Waters ACQUITY UPC2 system with YMC CHIRAL ART SC or SJ (4.6x250 mm, 3 µm) in a mixed solvent system of supercritical CO_2 and MeOH. A system backpressure of 138 bar was used in all cases.

Experiments which suggest against cation borylation occurring in situ

The cation was exposed to the borylation catalyst under the standard reaction conditions at both -10 °C (the reaction temperature) and 50 °C (the temperature of the pre-stir). In both cases, no change was modification of the cation was observed by analysis of the 1H-NMR spectrum, including no evidence of borylation of the hydroxyl group. We presume that this is disfavored here due to the sterically encumbered environment.

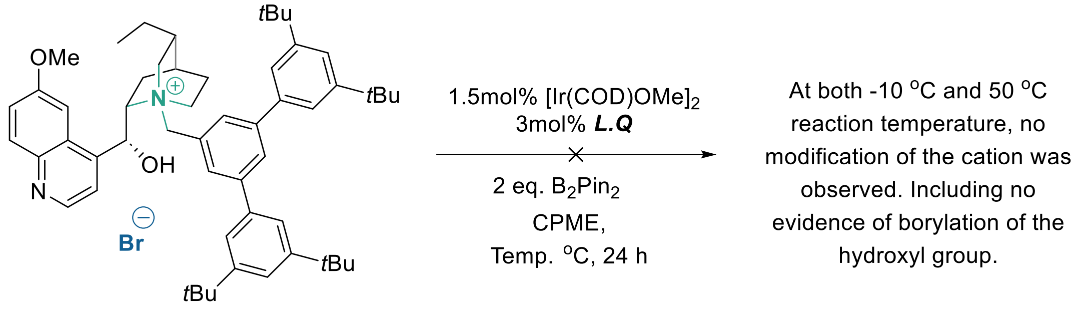

Catalyst Synthesis

L.Q and L.QD were synthesized according to literature procedures.[1] QDesEt.Br was synthesized according to literature procedure.[2]

(1R,2S,4R)-2-((R)-hydroxy(6-methoxyquinolin-4-yl)methyl)-1-((3,3'',5,5''-tetra-tert-butyl-[1,1':3',1''-terphenyl]-5'-yl)methyl)quinuclidin-1-ium (5'-methyl-[2,2'-bipyridin]-5-yl)methanesulfonate (L.QDesEt)

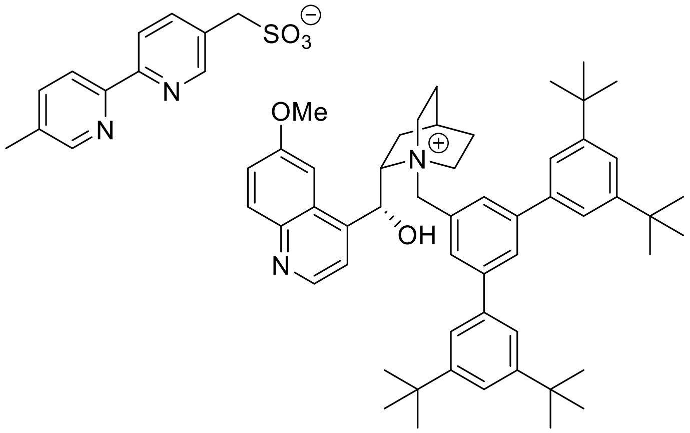

(1*S*,2*S*)-2-((*R*)-hydroxy(quinolin-4-yl)methyl)-1-((3,3'',5,5''-tetra-tert-butyl-[1,1':3',1''-terphenyl]-5'-yl)methyl)quinuclidin-1-ium bromide(**QDesEt.Br**) (38 mg; 0.05 mmol; 1eq) and silver(I) (5'-methyl-[2,2'-bipyridin]-5-yl)methanesulfonate (86% purity; 26 mg; 0.06 mmol; 1.2eq) were suspended in CH_2Cl_2 (4 ml) and water (4 ml) and stirred vigorously for 1 hour. The organic layer was then separated and the aqueous was reextracted with more CH_2Cl_2 (2x 4 ml). The organic layer was then dried over $MgSO_4$, filtered and concentrated. The residue then was dissolved in CH_2Cl_2, and filtered through a PTFE 0.2 µm syringe filter. The solvent was then removed and the title compound obtained as an off-white solid (37 mg, 0.04 mmol, 73 %).

^{1}H NMR (400 MHz; $CDCl_3$) δ 8.70 (d, *J* = 4.4 Hz, 1H), 8.60 (d, *J* = 1.7 Hz, 1H), 8.42 (s, 1H), 8.09 (d, *J* = 8.1 Hz, 1H), 8.00 (d, *J* = 9.2 Hz, 1H), 8.00 (d, *J* = 8.1 Hz, 1H), 7.91 (s, 1H), 7.82 (s, 2H), 7.78 (dd, *J* = 8.1, 2.1 Hz, 1H), 7.68 (d, *J* = 4.5 Hz, 1H), 7.55 (s, 2H), 7.54 (s, 4H), 7.51 (dd, *J* = 8.1, 1.5 Hz, 1H), 7.35 (dd, *J* = 9.2, 2.6 Hz, 1H), 7.25 (d, *J* = 2.3 Hz, 1H), 6.99 (d, *J* = 3.9 Hz, 1H), 6.69 (d, *J* = 2.7 Hz, 1H), 5.99 (d, *J* = 12.5 Hz, 1H), 4.49 (td, *J* =

11.4, 5.0 Hz, 1H), 4.42 (d, *J* = 12.5 Hz, 1H), 4.11 (d, *J* = 16.2 Hz, 1H), 4.09 (d, *J* = 16.2 Hz, 1H), 3.93 (s, 3H), 3.55 (t, *J* = 8.8 Hz, 1H), 3.29 (d, *J* = 11.7, 4.0 Hz, 1H), 3.22 (t, *J* = 8.2 Hz, 2H), 2.35 – 2.41 (m, 1H), 2.36 (s, 3H), 1.99 - 2.07 (m, 3H), 1.67 (sept, *J* = 10.4 Hz, 1H), 1.48 – 1.52 (m, 1H), 1.43 (s, 36H), 1.18 (t, *J* = 10.6 Hz, 1H). **^{13}C NMR (101 MHz; $CDCl_3$) δ** 158.0, 154.3, 153.5, 151.6, 150.7, 149.5, 147.9, 144.3, 144.2, 143.9, 139.6, 138.6, 137.2, 133.0, 132.2, 131.1, 130.1, 129.2, 127.7, 125.7, 122.3, 122.0, 120.8, 120.5, 120.4, 120.0, 101.6, 70.4, 65.4, 64.0, 57.2, 55.9, 54.6, 51.4, 35.1, 31.6, 29.7, 25.3, 23.9, 23.8, 20.4, 18.3. **HRMS:** m/z: calc'd for $[C_{12}H_{11}N_2O_3S]^-$ expected 263.0490, observed 263.0493. calc'd for $[C_{53}H_{69}N_2O_2]^+$ expected 765.5359, observed 765.5364. **$[\alpha]_D^{25.9}$ =** -76.0 (c 0.50 $CHCl_3$).

Synthesis of starting materials

1, 4 and 4 were synthesized according to literature procedures.[1]

Bis(3-chlorophenyl)(4-methoxybenzyl)phosphine oxide (7)

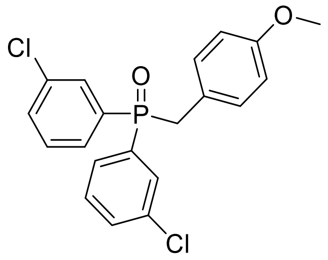

In a flame dried flask under argon atmosphere, magnesium turnings (511 mg; 21 mmol; 3eq) were suspended in dry THF (40 ml). 1-Bromo-3-chlorobenzene (2.47 ml; 21 mmol; 3eq) was added dropwise and the solution was refluxed for 3 hours. The mixture was then cooled to room temperature and diethyl phosphite (0.9 ml; 7 mmol; 1eq) was added dropwise and the reaction mixture was refluxed for 16 hours. Upon completion the reaction was cooled to room temperature and quenched with 3M $HCl_{(aq)}$. The mixture was extracted with ethyl acetate (2x 40 ml). The organics were combined, dried over $MgSO_4$ and concentrated. Under argon atmosphere the crude product was taken into dry THF (24 ml), cooled to 0 °C and sodium hydride (60% dispersion in mineral oil; 420 mg; 10.5 mmol; 1.5eq) was added. The mixture was stirred at this temperature for 10 min and then 4-methoxybenzyl chloride (1.04 ml; 7.7 mmol; 1.1eq) was added. The reaction was then stirred at room temperature for 16 hours. The reaction was quenched with water and the mixture was extracted with ethyl acetate (3x 50 ml). Organics combined, dried with $MgSO_4$ and concentrated. The crude was purified by column chromatography (50% to 60% ethyl acetate in petroleum ether 40-60 °C). The product was further purified by triturating the solid with Et_2O, filtering the solid and washing it with more Et_2O. This gave the product as a white solid (1.14 g; 42% over two steps).

^{1}H NMR (400 MHz, $CDCl_3$) δ 7.67 (dt, *J* = 11.6, 1.9 Hz, 2H), 7.57 (ddt, *J* = 11.0, 7.6, 1.3 Hz, 2H), 7.52 (ddt, *J* = 8.1, 2.1, 1.0 Hz, 2H), 7.42 (td, *J* = 7.8, 3.4 Hz, 2H), 7.04 (dd, *J* = 8.8, 2.3 Hz, 2H), 6.78 (d, *J* = 8.5 Hz, 2H), 3.78 (s, 3H), 3.61 (d, *J* = 13.3 Hz, 2H); **^{13}C NMR (101 MHz, $CDCl_3$)** δ 158.8 (d, *J* = 2.9 Hz), 135.2 (d, *J* = 15.2 Hz), 134.2 (d, *J* = 96.4 Hz), 132.2 (d, *J* = 2.6 Hz), 131.1 (d, *J* = 5.6 Hz), 131.0 (d, *J* = 9.6 Hz), 130.1 (d, *J* = 12.6 Hz), 129.1 (d, *J* = 8.7 Hz), 121.8 (d, *J* = 8.4 Hz), 114.1 (d, *J* = 2.5 Hz), 55.2 (s), 36.8 (d, *J* = 68.2 Hz); **^{31}P NMR (162 MHz, $CDCl_3$)** δ 27.5; **HRMS:** m/z: $[M + H]^+$ calc'd for $[C_{20}H_{18}Cl_2O_2P]^+$ expect 391.0416; found 391.0419.

Enantioselective borylation procedures

Data and procedures for compound 3b under the quoted conditions are given in the supporting information of ref 1 (Genov, G. R.; Douthwaite, J. L.; Lahdenperä, A. S. K.; Gibson, D. C.; Phipps, R. J. *Science*, **2020**, *367*, 1246–1251)**.**

(*S*)-2,2,2-trifluoro-*N*-((3-hydroxy-5-(trifluoromethyl)phenyl)(3-(trifluoromethyl)phenyl)methyl)acetamide (3a)

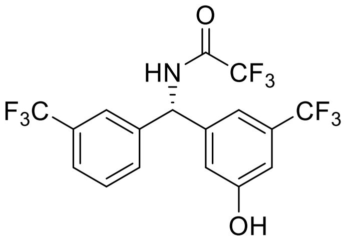

Reaction at +10°C with **L.Q**:

A stock solution of $[Ir(COD)OMe]_2$ (1.5 mg, 0.0022 mmol), B_2pin_2 (75 mg, 0.30 mmol) and ligand **L.Q** (4.8 mg, 0.0045 mmol) was dissolved in cyclopentyl methyl ether (0.75 ml) under an argon atmosphere and stirred at 50°C for 1 hour. This stock solution was then cooled to +10°C and 0.5 ml was transferred to a different vial containing *N*-(bis(3-(trifluoromethyl)phenyl)methyl)-2,2,2-trifluoroacetamide (41.4 mg, 0.1mmol) which had also been sealed and backfilled with argon. The reaction was then stirred at +10°C for 8 hours before the vial was opened and the solvent removed on a stream of air. The crude residue was then dissolved in MeOH (0.25 ml) and THF (0.25 ml), sodium bicarbonate (42 mg, 0.5 mmol, 5 eq) added before dropwise addition of H_2O_2 (100 vol, 0.12 ml, 1 mmol, 10 eq). The reaction was stirred at room temperature for 1 hour before the solvents were removed *in vacuo*. The residue was then dissolved in CH_2Cl_2 (5 ml) and H_2O (5 ml), separated and the aqueous layer extracted with CH_2Cl_2 (2 x 10 ml). The combined organic layers were dried over magnesium sulphate, filtered and concentrated. NMR analysis showed >95% conversion of starting material. Purification *via* silica gel chromatography (10-30 % EtOAc in 40-60 Pet. Ether) gave the title compound as a white powder (23.3 mg, 0.055 mmol, 55 % yield, 90 % *ee.*).

Reaction at -10°C with **L.QDesEt**:

A stock solution of $[Ir(COD)OMe]_2$ (1.5 mg, 0.0022 mmol), B_2pin_2 (75 mg, 0.30 mmol) and ligand **L.QDesEt** (4.6 mg, 0.0045 mmol) was dissolved in cyclopentyl methyl ether (0.75 ml) under an argon atmosphere and stirred at 50 °C for 1 hour. This stock solution was then cooled -10°C and 0.5 ml transferred to a different vial containing *N*-(bis(3-(trifluoromethyl)phenyl)methyl)-2,2,2-trifluoroacetamide (41.4 mg, 0.1mmol) which had also been sealed and backfilled with argon. The reaction was then stirred at -10°C for 24 hours before the vial was opened and the solvent removed on a stream of air. The crude residue was then dissolved in MeOH (0.25 ml) and THF (0.25 ml), sodium bicarbonate (42 mg, 0.5 mmol, 5 eq) added before dropwise addition of H_2O_2 (100 vol, 0.12 ml, 1 mmol, 10 eq). The reaction was stirred at room temperature for 1 hour before the solvents were removed *in vacuo*. The residue was then dissolved in CH_2Cl_2 (5 ml) and H_2O (5 ml), separated and the aqueous layer extracted with CH_2Cl_2 (2 x 10 ml). The combined organic layers were dried over magnesium sulphate, filtered and concentrated. Purification *via* silica gel chromatography (10-30 % EtOAc in 40-60 Pet. Ether) gave the title compound as a white powder (24.0 mg, 0.056 mmol, 56 % yield, 87 % *ee.*).

^{1}H NMR (400 MHz; $CDCl_3$) δ 8.90-9.00 (br s, 1H), 7.59 (d, *J* = 7.6 Hz, 1H), 7.51 (s, 1H), 7.48 (t, *J* = 7.7 Hz, 1H), 7.43 (d, *J* = 7.7 Hz), 7.02 (s, 1H), 6.96 (s, 1H), 6.86 (s, 1H), 6.28 (s, 1H). **^{13}C NMR (101 MHz; $CDCl_3$) δ** 157.8, 157.1 (q, *J* = 37.9 Hz), 141.4, 140.1, 132.5 (q, *J* = 32.5 Hz), 131.3 (q, *J* = 32.4 Hz), 131.0, 129.4, 125.0 (q, *J* = 3.6 Hz), 124.0 (q, *J* = 3.8 Hz), 123.8 (q, *J* = 272.4 Hz), 123.6 (q, *J* = 272.0 Hz), 117.9, 115.9 (q, *J* = 287.8 Hz), 115.3

(q, *J* = 3.8 Hz), 112.3 (q, *J* = 3.7 Hz), 56.4. **^{19}F NMR (376 MHz; CDCl$_3$) δ** -63.9, -64.0, -76.6. **HPLC Analysis:** CHIRALPAK AD-H, 5% i-Propanol in Hexane, 1mlmin^{-1}, 30 °C, t_R = 7.6 (major), 10.2 (minor) minutes. Data matches that previously reported.[1]

The experimental procedure and data for the enantioselective borylation of *meta*-bromo compound **3b** at + 10 °C has previously been reported in the Supporting Information (page S144) of ref 1.

(S)-2,2,2-trifluoro-N-((3-hydroxy-5-methylphenyl)(m-tolyl)methyl)acetamide (3c)

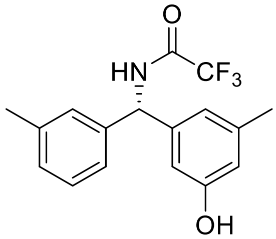

A stock solution of $[Ir(COD)OMe]_2$ (1.5 mg, 0.0022 mmol), B_2pin_2 (75 mg, 0.30 mmol) and ligand **L.Q** (4.8 mg, 0.0045 mmol) was dissolved in cyclopentyl methyl ether (0.75 ml) under an argon atmosphere and stirred at 50°C for 1 hour. This stock solution was then cooled to +10°C and 0.5 ml transferred to a different vial containing *N*-(bis(3-(methyl)phenyl)methyl)-2,2,2-trifluoroacetamide (30.9 mg, 0.1mmol) which had also been sealed and backfilled with argon. The reaction was then stirred at +10°C for 24 hours before the vial was opened and the solvent removed on a stream of air. NMR analysis in $CDCl_3$ showed 68 % mono-borylation, 14 % di-borylation with 18 % remaining starting material. The crude residue was then dissolved in MeOH (0.25 ml) and THF (0.25 ml), sodium bicarbonate (42 mg, 0.5 mmol, 5 eq) added before dropwise addition of H_2O_2 (100 vol, 0.12 ml, 1 mmol, 10 eq). The reaction was stirred at room temperature for 1 hour before the solvents were removed *in vacuo*. The residue was then dissolved in CH_2Cl_2 (5 ml) and H_2O (5 ml), separated and the aqueous layer extracted with CH_2Cl_2 (2 x 10 ml). The combined organic layers were dried over magnesium sulphate, filtered and concentrated. Purification *via* silica gel chromatography (10-30 % EtOAc in 40-60 Pet. Ether) gave the title compound as a white powder (13.2 mg, 0.041 mmol, 41 % yield, 69 % *ee*).

^{1}H NMR (400 MHz; CDCl$_3$) δ 7.28 (t, *J* = 7.9 Hz, 1H), 7.16 (d, *J* = 7.5 Hz, 1H), 7.05 (s, 1H), 6.91 (d, *J* = 8.4 Hz, 1H), 6.82 (d, *J* = 6.0 Hz), 6.63 (s, 2H), 6.50 (s, 1H), 6.11 (d, *J* = 8.0 Hz), 2.36 (s, 3H), 2.31 (s, 3H). **^{13}C NMR (101 MHz; CDCl$_3$) δ** 156.5, 156.4 (q, *J* = 37.3 Hz), 141.0, 140.6, 139.2, 138.8, 129.0, 128.9, 127.9, 124.2, 120.4, 115.9, 115.8 (q, *J* = 288.0 Hz), 111.3, 57.4, 29.7, 21.4. **^{19}F NMR (376 MHz; CDCl$_3$) δ** -75.6. **SFC Analysis:** CHIRAL ART SJ (CO_2/MeOH = 96/4, 2.5 ml min^{-1}, 40 °C) t_R = 9.1 (major), 9.7 (minor) minutes. Data matches that previously reported.[1]

(*R*)-*P*-(3-chloro-5-hydroxyphenyl)-*P*-(3-chlorophenyl)-*N*-(4-methoxyphenyl)phosphinic amide (5)

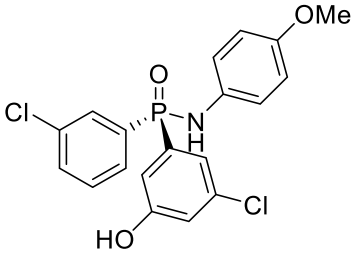

A stock solution of $[Ir(COD)OMe]_2$ (1.5 mg, 0.0022 mmol), B_2pin_2 (75 mg, 0.30 mmol) and ligand (4.6 or 4.8 mg depending on ligand, 0.0045 mmol) was dissolved in cyclopentyl methyl ether (0.75 ml) under an argon atmosphere and stirred at 50°C for 1 hour. This stock solution was then cooled to 0°C and 0.5 ml transferred to a different vial containing *P,P*-bis(3-chlorophenyl)-*N*-(4-methoxyphenyl)phosphinic amide (39 mg, 0.1mmol) which had also been sealed and backfilled with argon. The reaction was then stirred at 0°C for 6 hours before the vial was opened and the solvent removed on a stream of air. The crude residue was then dissolved in MeOH (0.25 ml) and THF (0.25 ml), sodium bicarbonate (42 mg, 0.5 mmol, 5 eq) added before dropwise addition of H_2O_2 (100 vol, 0.12 ml, 1 mmol, 10 eq). The reaction was stirred at room temperature for 1 hour before the solvents were removed *in vacuo*. The residue was then dissolved in CH_2Cl_2 (5 ml) and H_2O (5 ml), separated and the aqueous layer extracted with CH_2Cl_2 (2 x 10 ml). The combined organic layers were dried over magnesium sulphate, filtered and concentrated. Purification *via* silica gel chromatography (10-30 % EtOAc in CH_2Cl_2) gave the title compound as a white powder.

With **L.QD**: 15.9 mg, 0.026 mmol, 26 % yield, -44 % *ee.*

With **L.QDesEt**: 27.0 mg, 0.066 mmol, 66 % yield, 52 % *ee.*

^{1}H NMR (400 MHz; $CDCl_3$:MeOD 1:2) δ 7.83 (d, *J* = 12.3 Hz, 1H), 7.74 (dd, *J* = 12.7, 9.0 Hz, 1H), 7.57 (d, *J* = 8.0 Hz, 1H), 7.48 (td, *J* = 8.0, 2.9 Hz, 1H), 7.28 (d, *J* = 12.3 Hz, 1H), 7.17 (d, *J* = 14.5 Hz, 1H), 7.01 (m, 3H), 6.74 (d, *J* = 8.3 Hz, 2H), 3.72 (s, 3H). **^{13}C NMR (101 MHz; $CDCl_3$:MeOD 1:2) δ** 158.7 (d, *J* = 18.4 Hz), 155.3, 135.4 (d, *J* = 21.0 Hz), 134.9 (d, *J* = 17.1 Hz), 133.9 (d, *J* = 130.0 Hz), 133.6 (d, *J* = 129.9 Hz), 133.2, 132.3 (d, *J* = 2.8 Hz), 131.6 (d, *J* = 11.0 Hz), 130.2 (d, *J* = 14.5 Hz), 130.1 (d, *J* = 9.5 Hz), 122.2 (d, *J* = 10.9 Hz), 120.8 (d, *J* = 6.9 Hz), 119.4, 117.1 (d, *J* = 10.7 Hz), 114.2, 54.7. **^{31}P NMR (162 MHz; $CDCl_3$:MeOD 1:2) δ** 18.0. **SFC Analysis:** CHIRAL ART SC (CO2/MeOH = 85/15, 2.5 mL min^{-1}, 40 °C) t_R = 6.1 (minor), 7.0 (major) minutes. Data matches that previously reported.[1]

(3-chloro-5-hydroxyphenyl)(3-chlorophenyl)(4-methoxybenzyl)phosphine oxide (8)

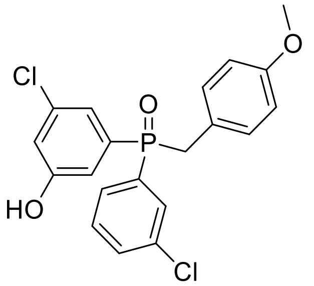

A stock solution of $[Ir(COD)OMe]_2$ (1.5 mg, 0.0022 mmol), B_2pin_2 (75 mg, 0.3 mmol) and ligand **L.Q** (4.8 mg, 0.005 mmol) was dissolved in cyclopentyl methyl ether (0.75 ml) under an argon atmosphere and stirred at 50°C for 1 hour. This stock solution was then cooled to 0°C and 0.5 ml transferred to a different vial containing bis(3-chlorophenyl)(4-methoxybenzyl)phosphine oxide (39.1 mg, 0.1 mmol) which had also been sealed and backfilled with argon. The reaction was then stirred at 0°C for 6 hours before the vial was opened and the solvent removed on a stream of air. The crude residue was then dissolved in MeOH (0.25 ml) and THF (0.25 ml), sodium bicarbonate (42 mg, 0.5 mmol, 5 eq) added before dropwise addition of H_2O_2 (100 vol, 0.12 ml, 1 mmol, 10 eq). The reaction was stirred at room temperature for 1 hour before the solvents were removed *in vacuo*. The residue was then dissolved in CH_2Cl_2 (5 ml) and H_2O (5 ml), separated and the aqueous layer extracted with CH_2Cl_2 (2 x 10 ml). The combined organic layers were dried over magnesium sulphate, filtered and concentrated. Purification by silica gel chromatography (15 % to 35% EtOAc in CH_2Cl_2) gave the title compound as a colourless oil (14 mg). A small amount of pinacol impurity was found to be inseparable from

the final product on silica or by trituration with diethyl ether. Corrected yield - 12.8 mg, 0.031 mmol, 31% yield, 65% *ee*.

^{1}H NMR (400 MHz, MeOD) δ 7.73 (dt, *J* = 11.7, 1.7 Hz, 1H), 7.67 (ddt, *J* = 11.2, 7.6, 1.3 Hz, 1H), 7.59 (ddt, *J* = 8.0, 2.2, 1.1 Hz, 1H), 7.51 (td, *J* = 7.8, 3.4 Hz, 1H), 7.19 (ddd, *J* = 11.6, 1.9, 1.2 Hz, 1H), 7.10 – 7.01 (m, 3H), 6.99 (t, *J* = 1.8 Hz, 2H), 6.76 (d, *J* = 8.4 Hz, 2H), 3.78 (d, *J* = 13.3 Hz, 2H), 3.72 (s, 3H); **^{13}C NMR (101 MHz, MeOD)** δ 159.0, 158.9 (d, *J* = 13.9 Hz), 135.4 (d, *J* = 18.8 Hz), 134.8 (d, *J* = 15.4 Hz), 134.2 (d, *J* = 97.6 Hz), 133.7 (d, *J* = 97.5 Hz), 132.1 (d, *J* = 2.7 Hz), 131.0 (d, *J* = 5.4 Hz), 130.5 (d, *J* = 10.1 Hz), 130.2 (d, *J* = 12.8 Hz), 129.0 (d, *J* = 9.2 Hz), 121.8 (d, *J* = 8.9 Hz), 120.9 (d, *J* = 10.5 Hz), 119.0 (d, *J* = 2.4 Hz), 116.1 (d, *J* = 9.9 Hz), 113.5 (d, *J* = 2.8 Hz), 54.2, 35.0 (d, *J* = 68.3 Hz); **^{31}P NMR (162 MHz, MeOD)** δ 31.8; **HRMS:** m/z: $[M + H]^+$ calc'd for $[C_{20}H_{18}Cl_2O_3P]^+$ expect 407.0365; found 407.0367; **SFC Analysis**: CHIRAL ART SJ (CO_2/MeOH = 93/7, 2.5 mL min^{-1}, 40 °C) t_R = 12.2 (major), 13.1 (minor) minutes; **$[\alpha]_D^{25.0}$** = +5.1 (*c* 0.91, $CHCl_3$).

SFC and HPLC Traces

(*S*)-2,2,2-trifluoro-*N*-((3-hydroxy-5-(trifluoromethyl)phenyl)(3-(trifluoromethyl)phenyl)methyl)acetamide (3a)

HPLC Conditions: : CHIRALPAK AD-H, 5% i-Propanol in Hexane, 1mlmin^{-1}, 30 °C, t_R = 7.6 (major), 10.2 (minor) minutes.

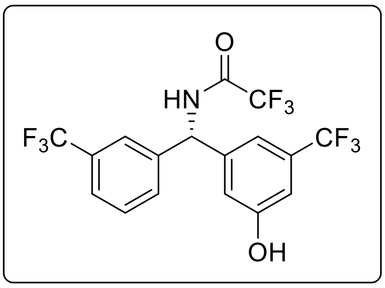

3a, 87 % *ee*

(with **L.QDesEt** at -10°C)

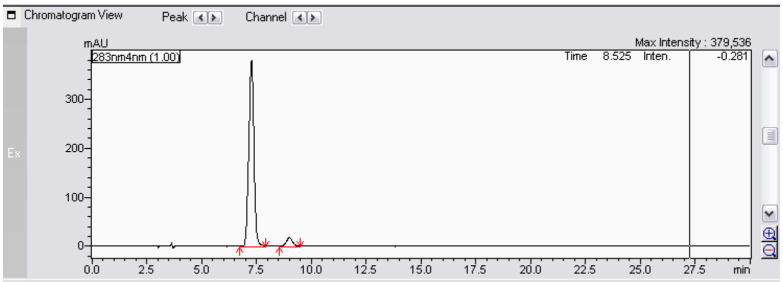

Peak#	Ret. Time	Area	Height	Area%
1	7.262	6263224	381219	93.743
2	8.991	418045	19282	6.257
Total		6681269	400501	100.000

Extracted 283nm / Ch1 254nm

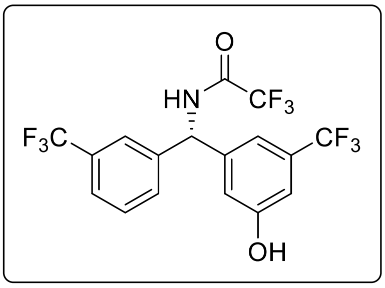

3a, 90 % *ee*

(with **L.Q** at +10°C)

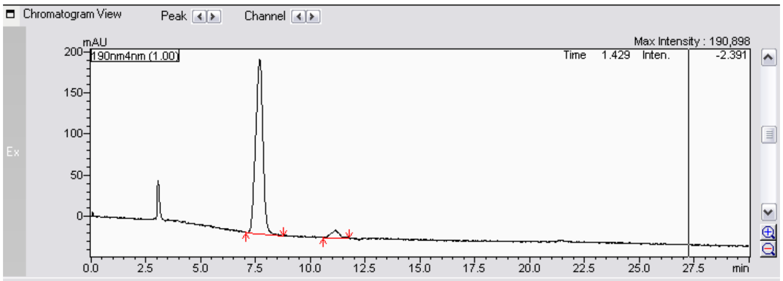

Peak#	Ret. Time	Area	Height	Area%
1	7.691	4944714	212510	94.807
2	11.154	270838	9579	5.193
Total		5215552	222088	100.000

Extracted 190nm / Ch1 254nm

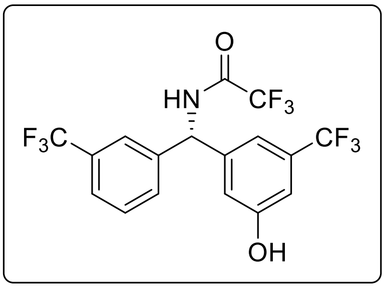

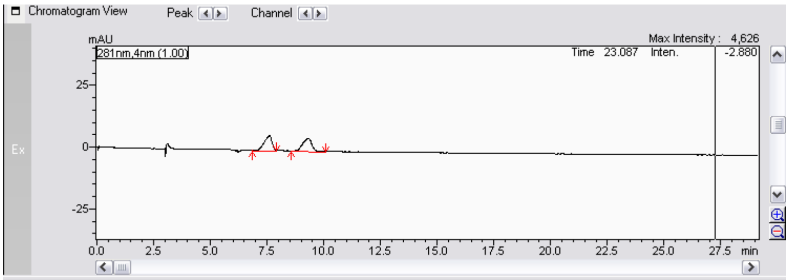

Peak#	Ret. Time	Area	Height	Area%
1	7.596	151988	6131	50.156
2	9.325	151039	5286	49.844
Total		303027	11417	100.000

Extracted 281nm / Ch1 254nm

(S)-2,2,2-trifluoro-N-((3-hydroxy-5-methylphenyl)(m-tolyl)methyl)acetamide (3c)

SFC Analysis: CHIRAL ART SJ (CO_2/MeOH = 96/4, 2.5 ml min^{-1}, 40 °C) t_R = 9.1 (major), 9.7 (minor) minutes.

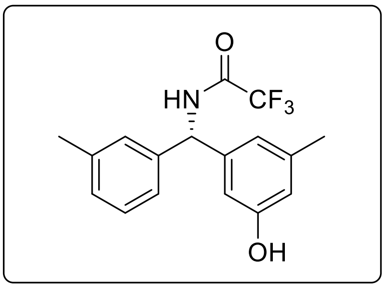

3c, 69 % *ee*

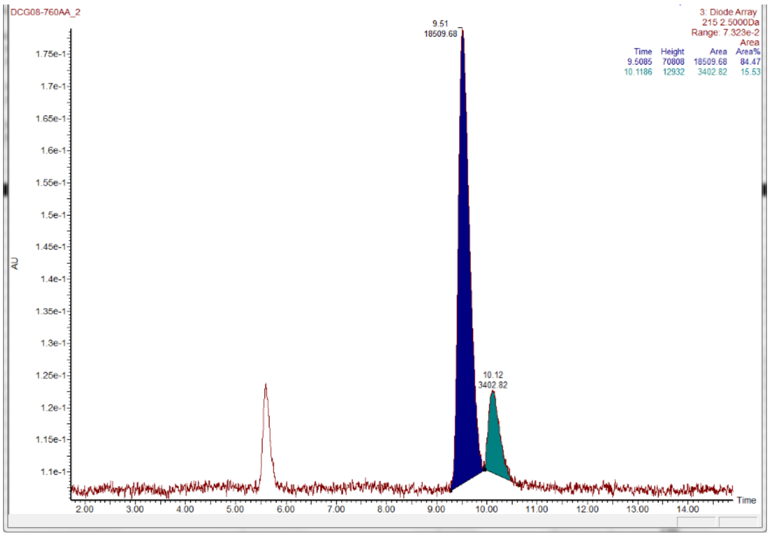

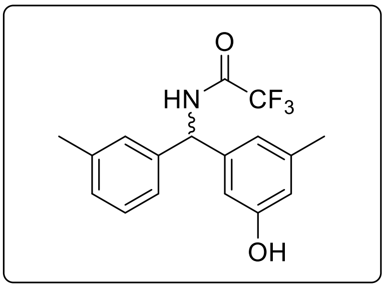

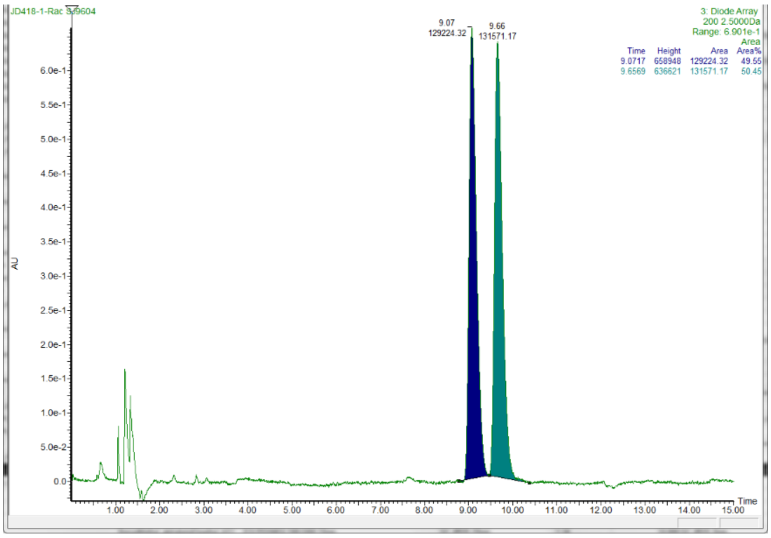

(*R*)-*P*-(3-chloro-5-hydroxyphenyl)-*P*-(3-chlorophenyl)-*N*-(4-methoxyphenyl)phosphinic amide (5)

SFC Analysis: CHIRAL ART SC (CO2/MeOH = 85/15, 2.5 mL min^{-1}, 40 °C) t_R = 6.1 (minor), 7.0 (major) minutes.

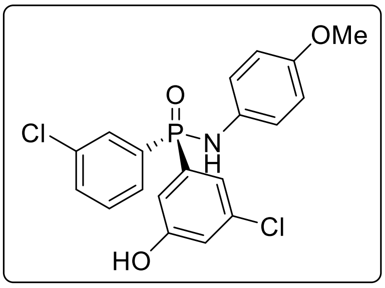

5, 52 % % *ee*

(with **L.QDesEt**)

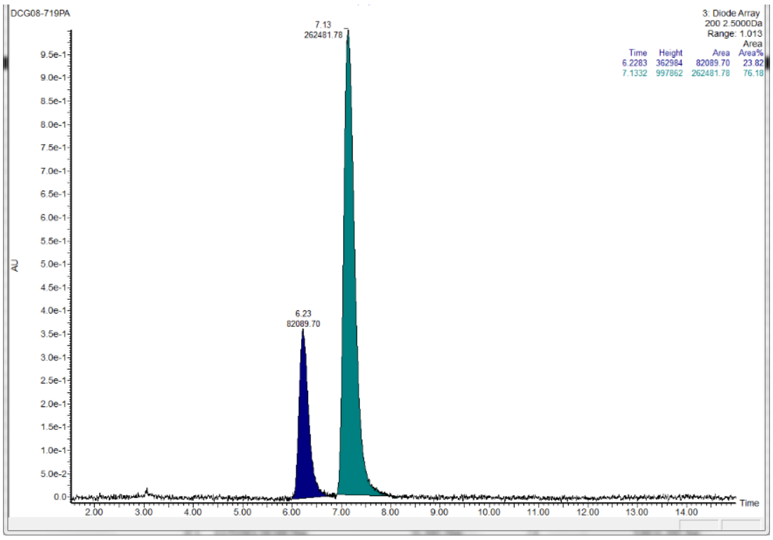

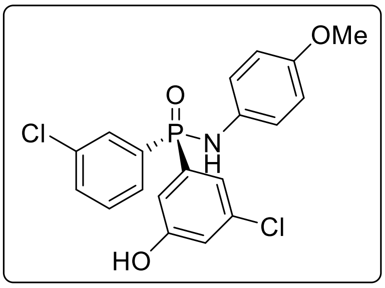

5, -44 % *ee*

*(*With **L.QD)**

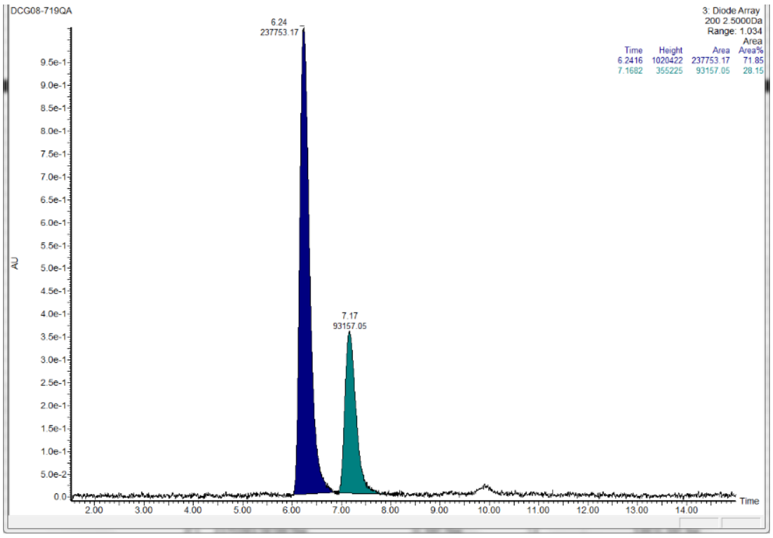

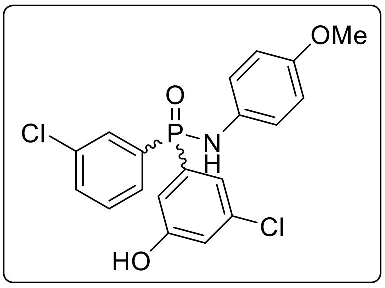

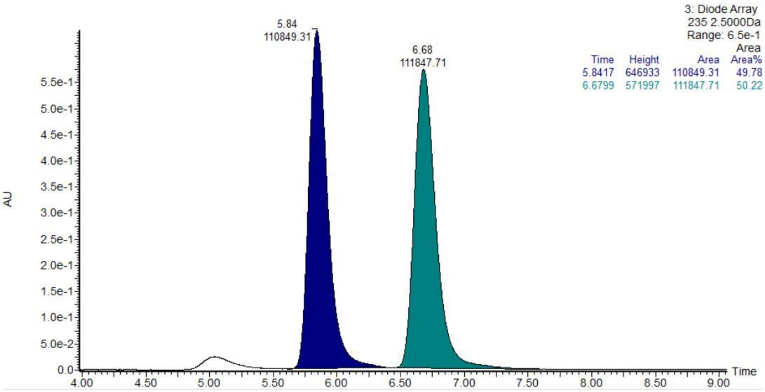

(*R*)-(3-chloro-5-hydroxyphenyl)(3-chlorophenyl)(4-methoxybenzyl)phosphine oxide (8)

SFC Analysis: CHIRAL ART SJ (CO_2/MeOH = 93/7, 2.5 mL min^{-1}, 40 °C) t_R = 12.2 (major), 13.1 (minor) minutes;

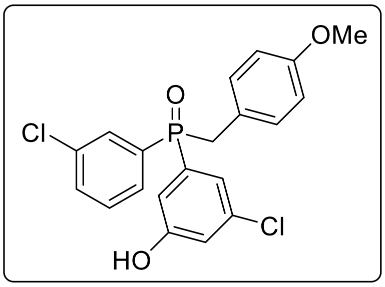

8, 65 % *ee*

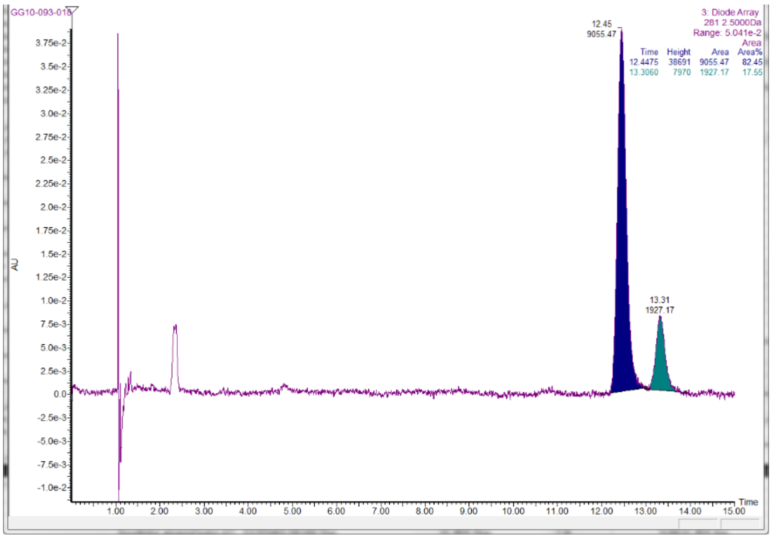

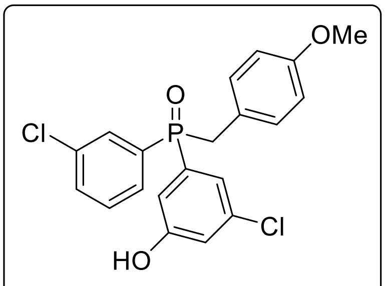

racemic

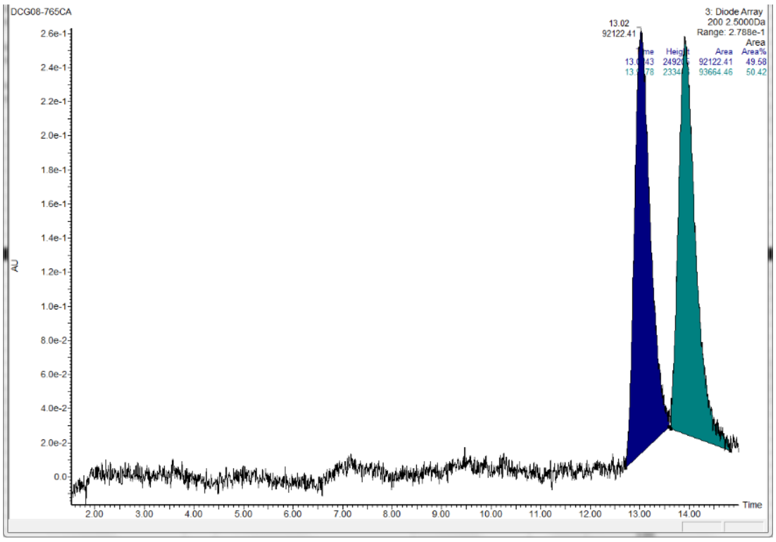

NMR Spectra

(1R,2S,4R)-2-((R)-hydroxy(6-methoxyquinolin-4-yl)methyl)-1-((3,3'',5,5''-tetra-tert-butyl-[1,1':3',1''-terphenyl]-5'-yl)methyl)quinuclidin-1-ium (5'-methyl-[2,2'-bipyridin]-5-yl)methanesulfonate (L.QDesEt) ^{1}H NMR (400 MHz, $CDCl_3$)

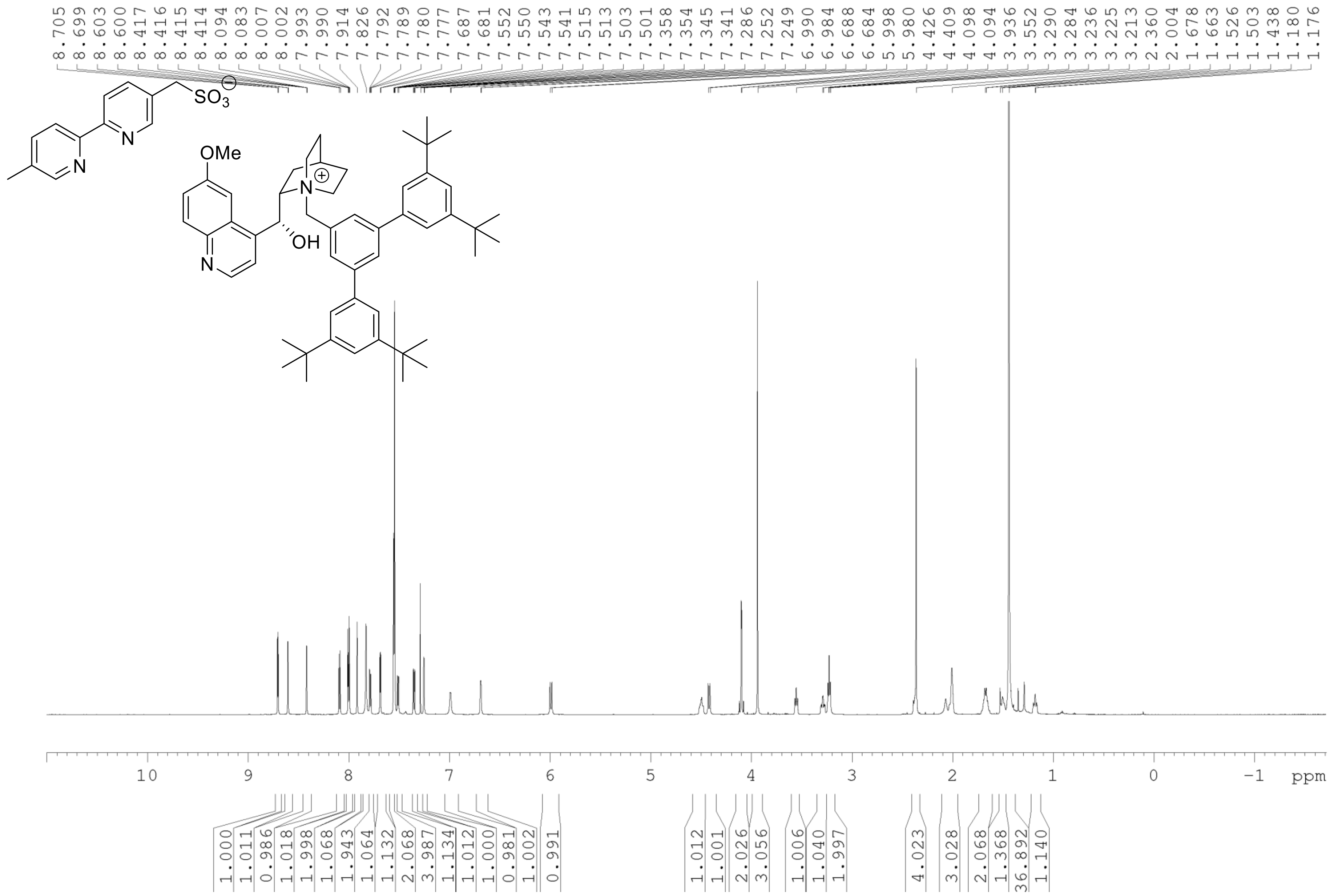

(1R,2S,4R)-2-((R)-hydroxy(6-methoxyquinolin-4-yl)methyl)-1-((3,3'',5,5''-tetra-tert-butyl-[1,1':3',1''-terphenyl]-5'-yl)methyl)quinuclidin-1-ium (5'-methyl-[2,2'-bipyridin]-5-yl)methanesulfonate (L.QDesEt) 13C NMR (101 MHz, CDCl3)

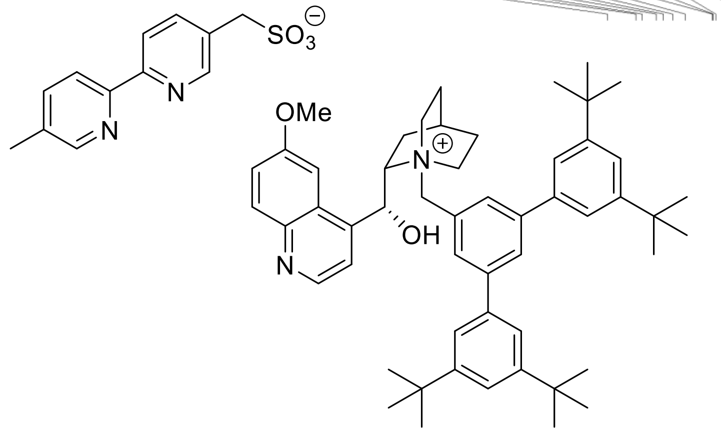

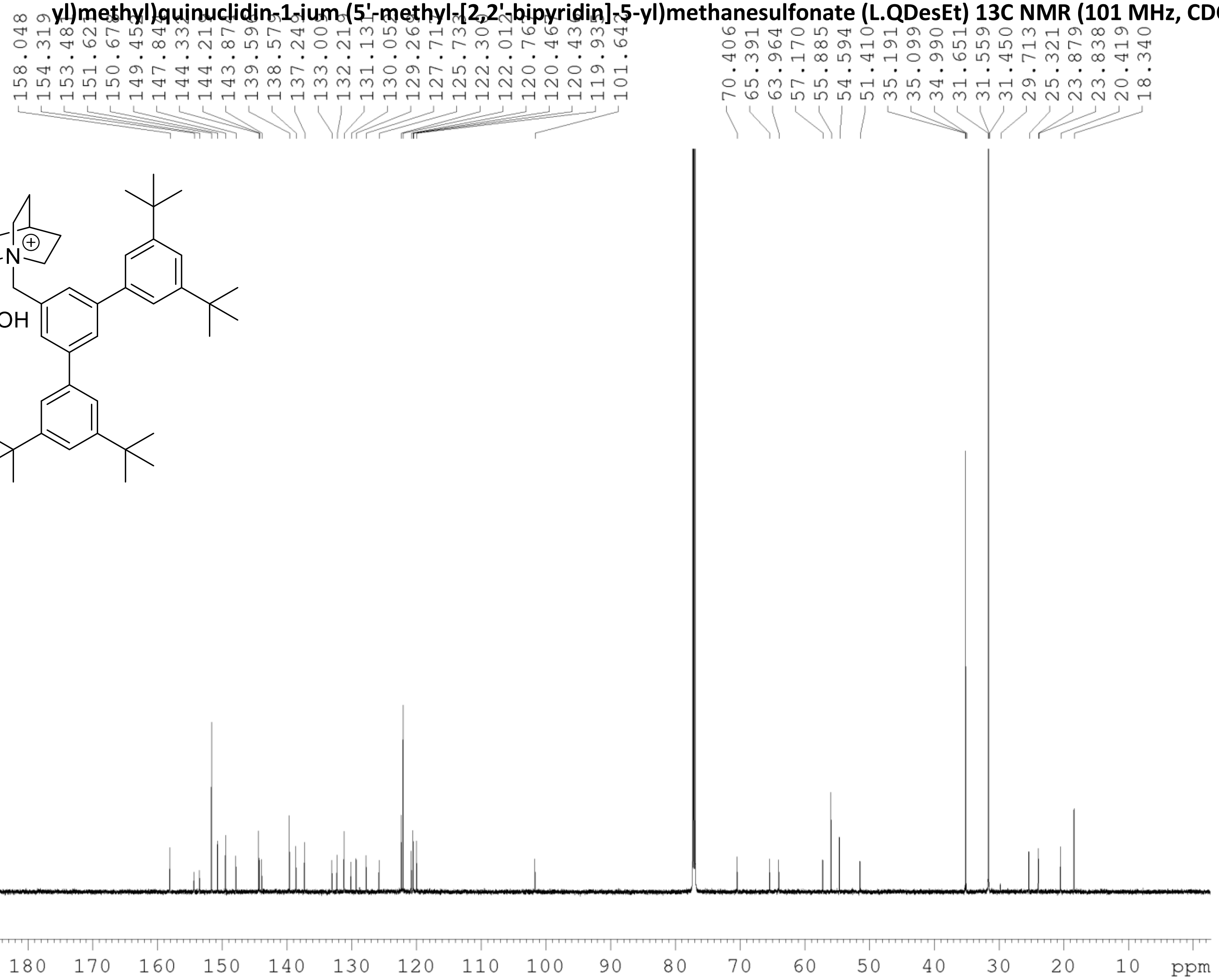

Bis(3-chlorophenyl)(4-methoxybenzyl)phosphine oxide (7) ^{1}H NMR (400 MHz, $CDCl_3$)

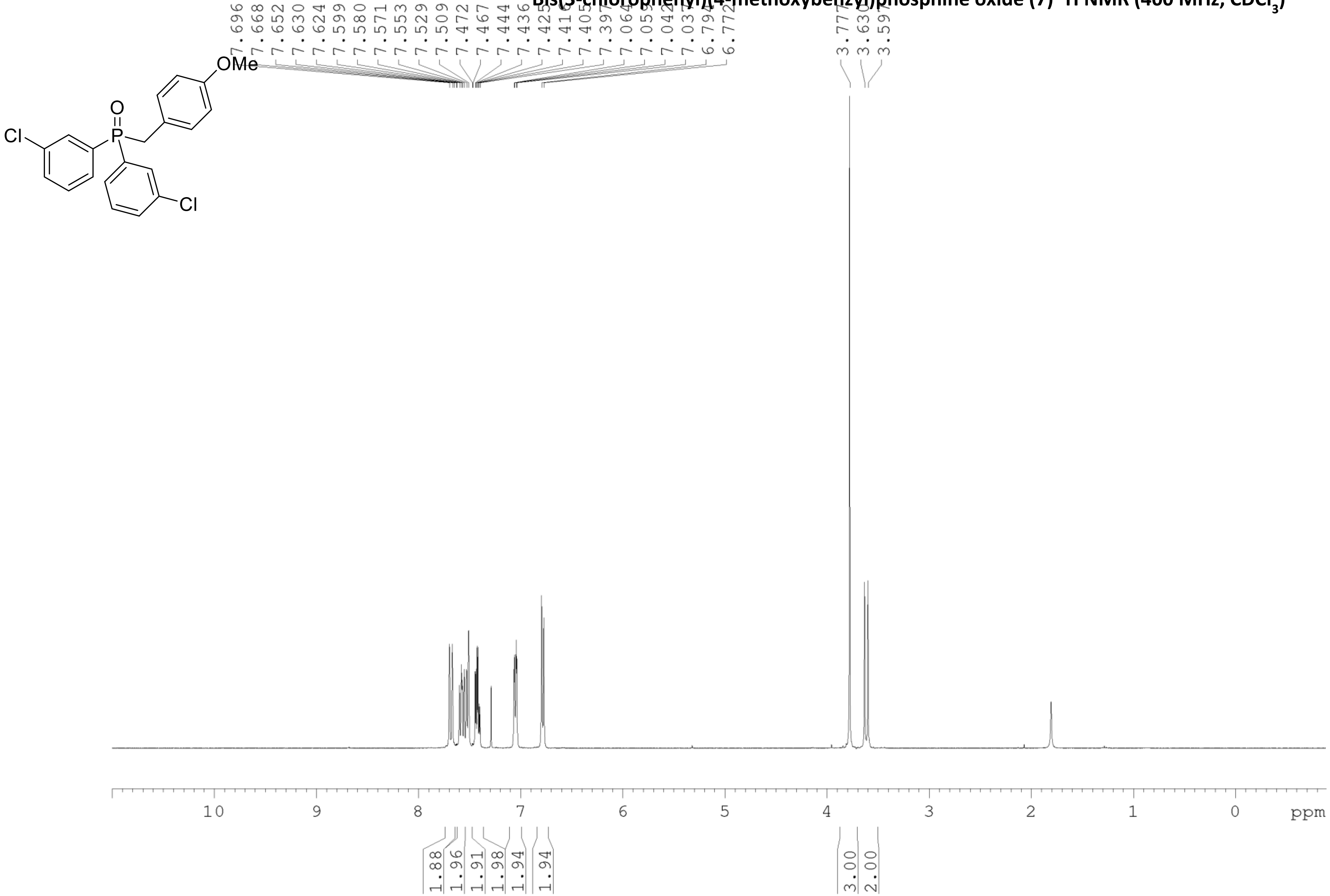

Bis(3-chlorophenyl)(4-methoxybenzyl)phosphine oxide (7) ^{13}C NMR (101 MHz, $CDCl_3$)

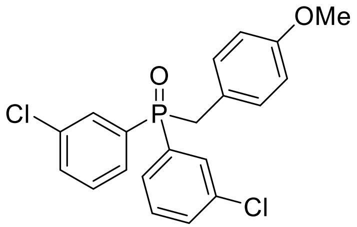

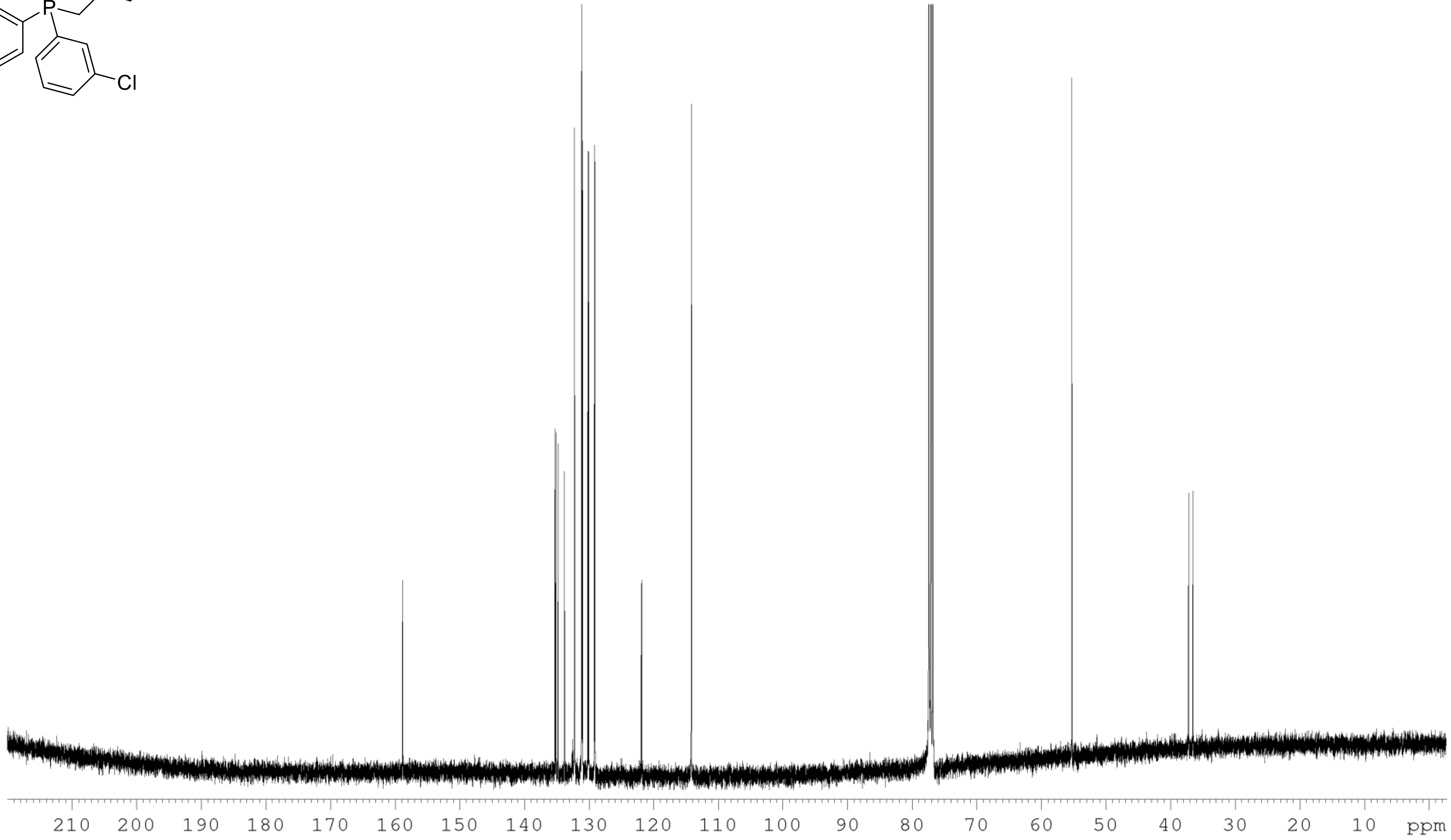

Bis(3-chlorophenyl)(4-methoxybenzyl)phosphine oxide (7) ^{31}P NMR (162 MHz, $CDCl_3$)

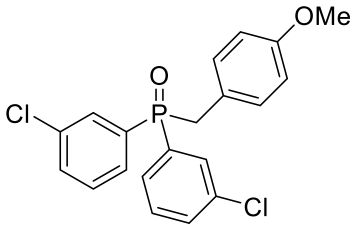

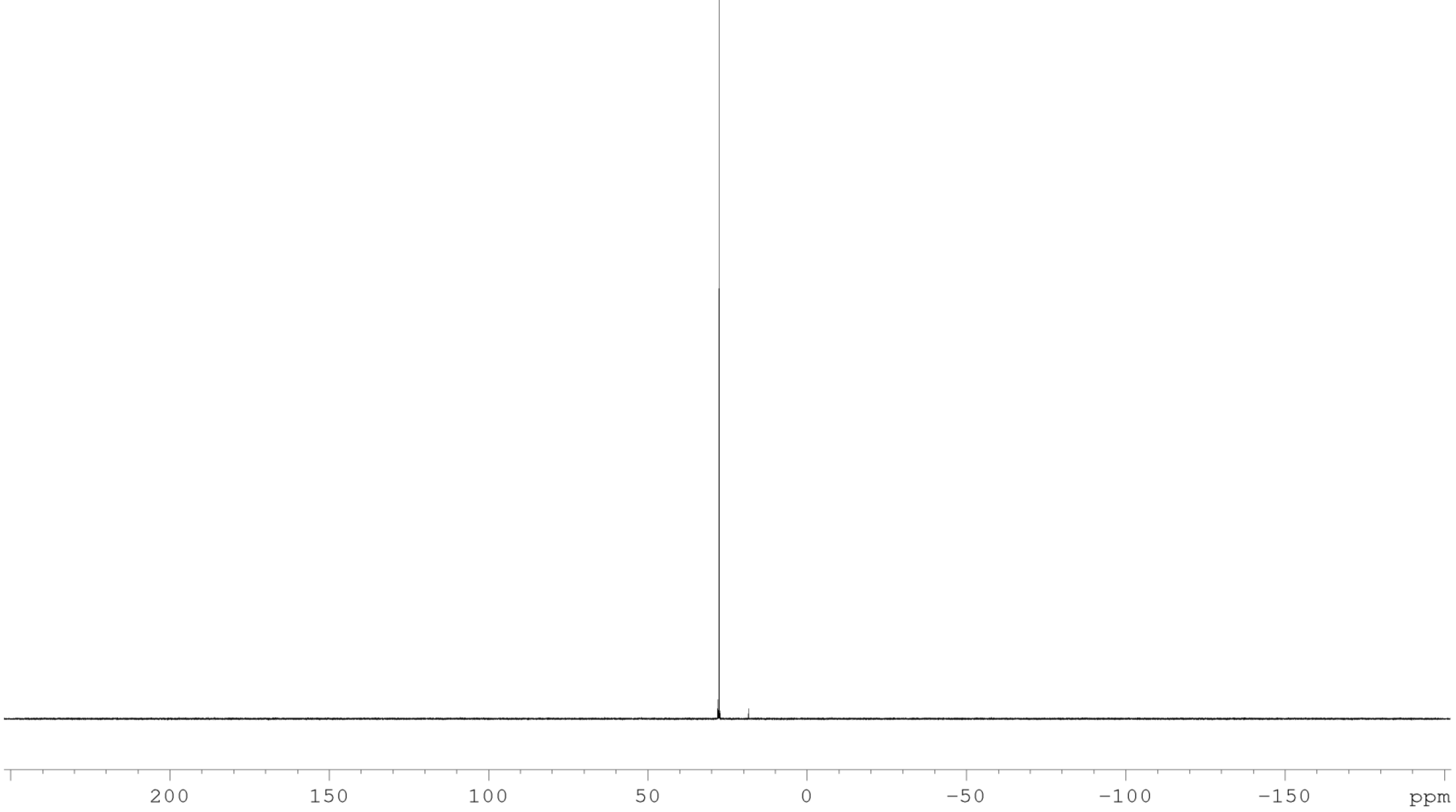

(*S*)-2,2,2-trifluoro-*N*-((3-hydroxy-5-(trifluoromethyl)phenyl)(3-(trifluoromethyl)phenyl)methyl)acetamide (3a)

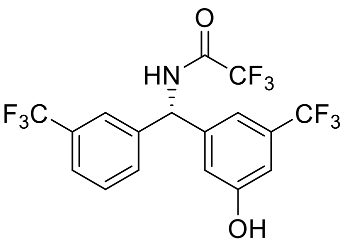

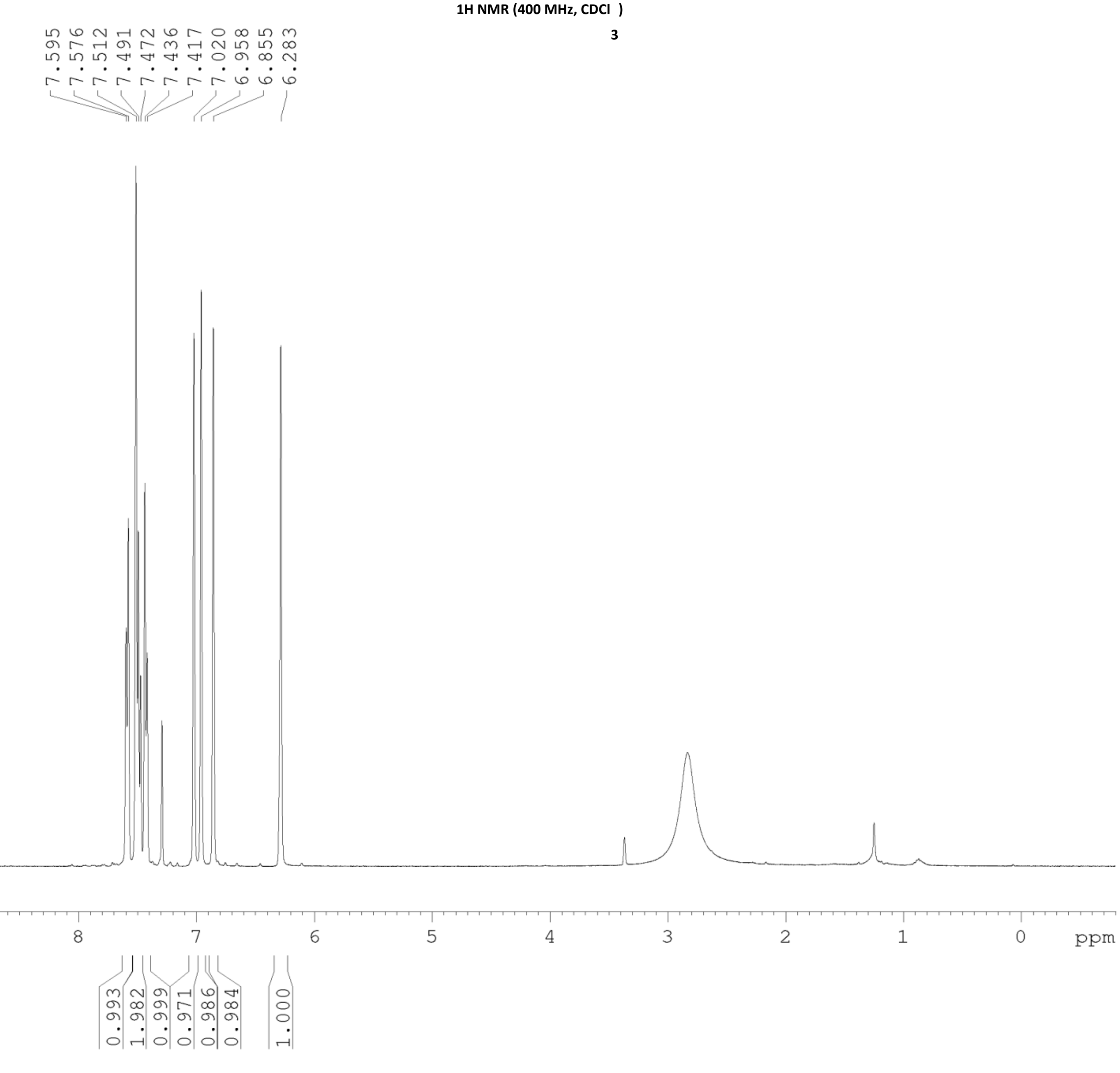

(*S*)-2,2,2-trifluoro-*N*-((3-hydroxy-5-(trifluoromethyl)phenyl)(3-(trifluoromethyl)phenyl)methyl)acetamide (3a) **^{13}C NMR (101 MHz, $CDCl_3$)**

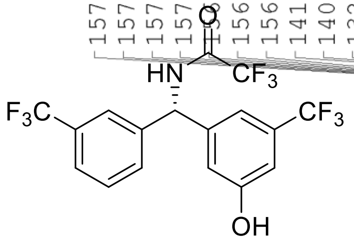

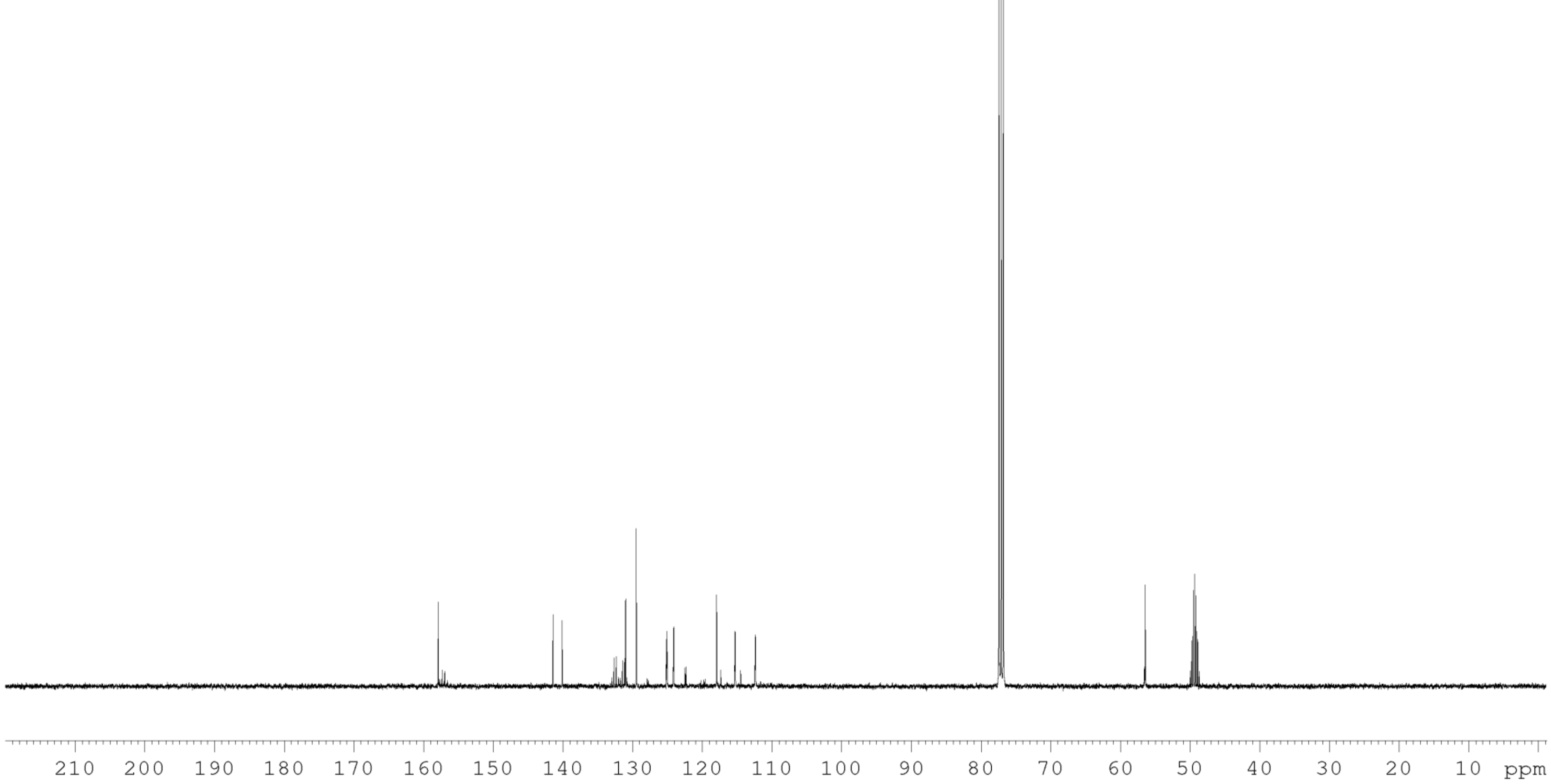

(*S*)-2,2,2-trifluoro-*N*-((3-hydroxy-5-(trifluoromethyl)phenyl)(3-(trifluoromethyl)phenyl)methyl)acetamide (3a) ^{19}F NMR (176 MHz, $CDCl_3$)

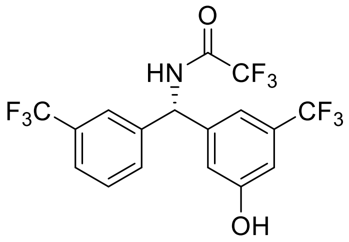

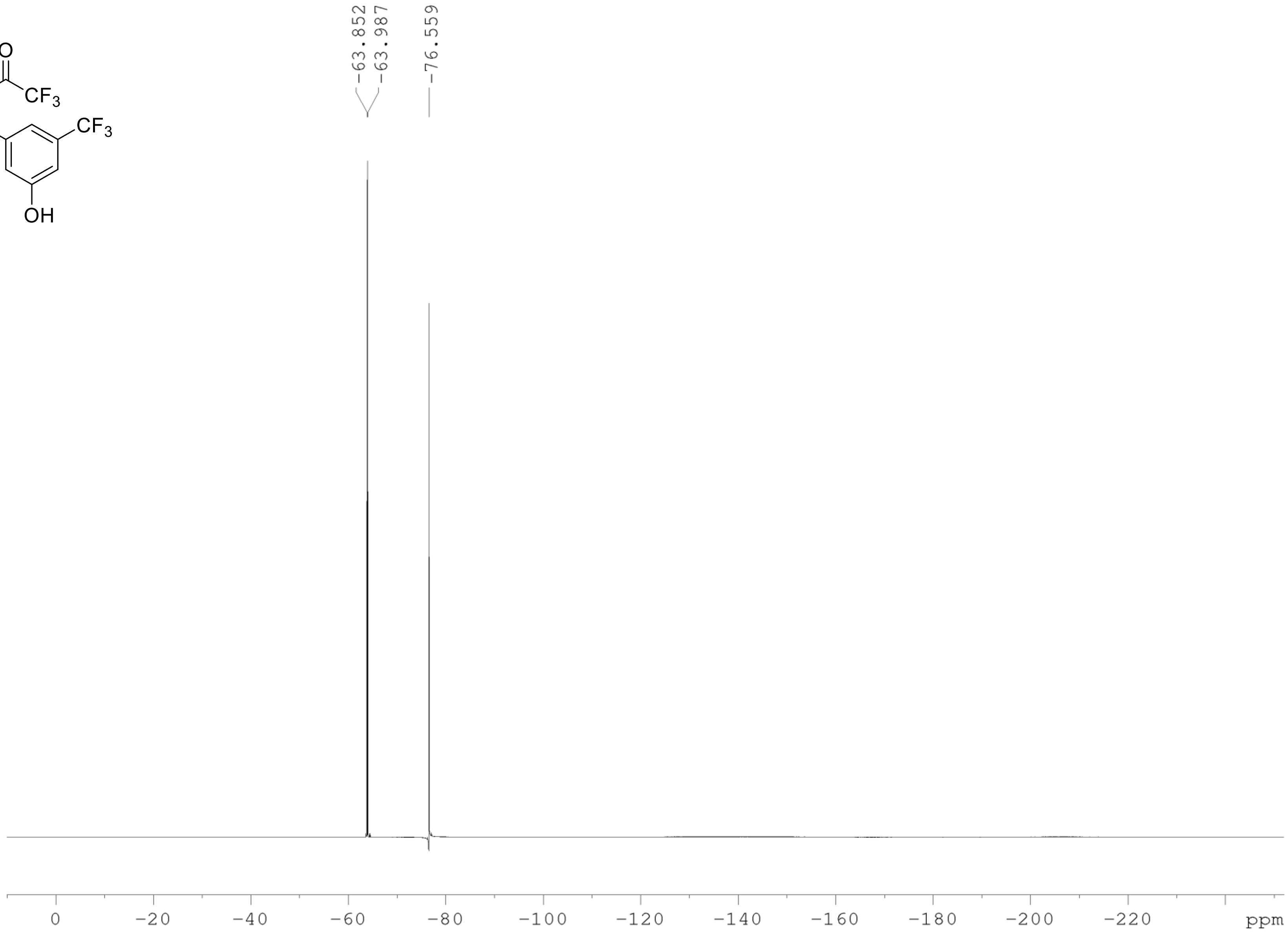

(S)-2,2,2-trifluoro-N-((3-hydroxy-5-methylphenyl)(m-tolyl)methyl)acetamide (3c) ^{1}H NMR (400 MHz, $CDCl_3$)

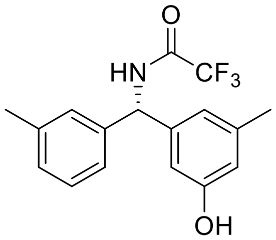

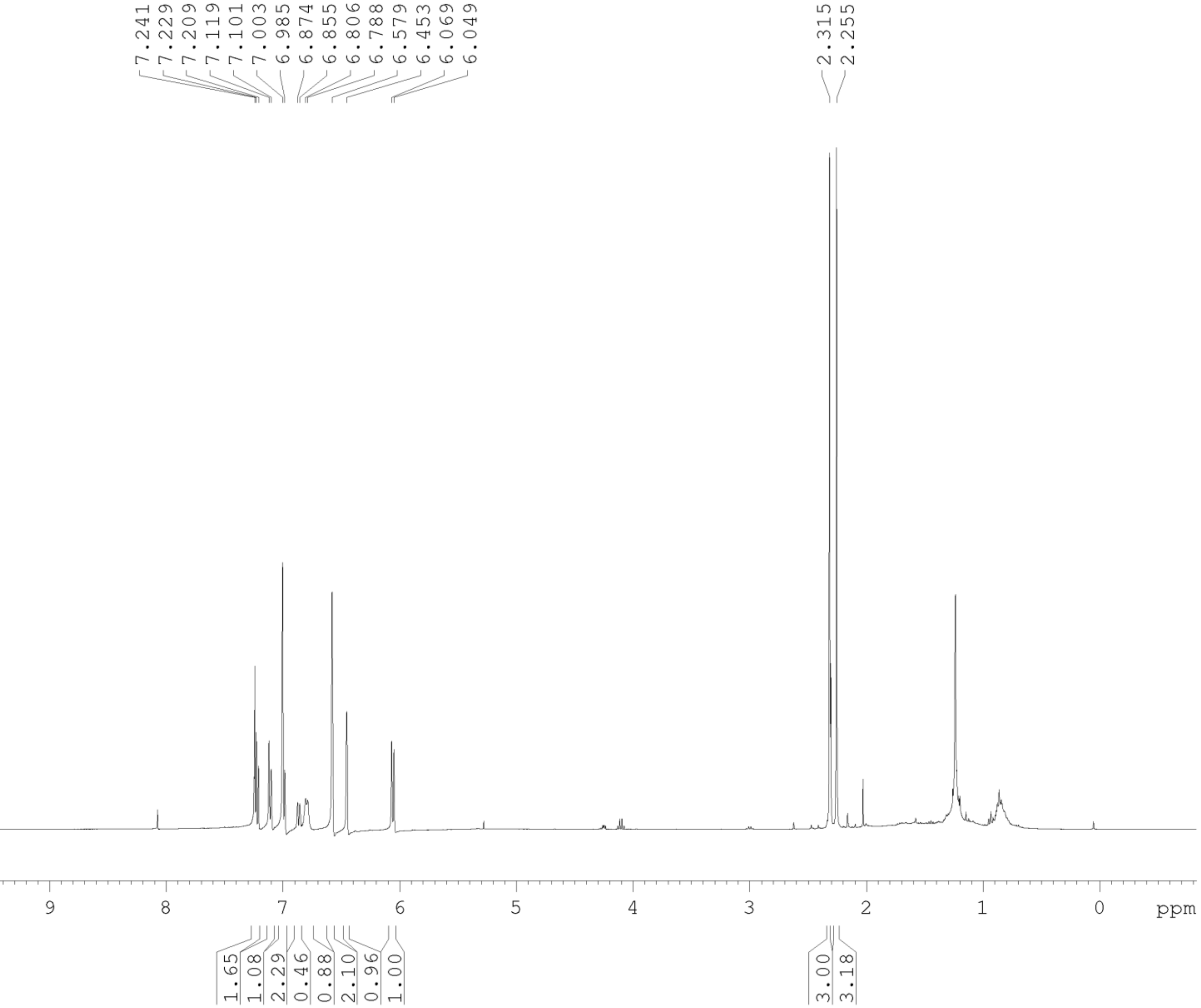

(S)-2,2,2-trifluoro-N-((3-hydroxy-5-methylphenyl)(m-tolyl)methyl)acetamide (3c) ^{13C} NMR (101 MHz, $CDCl_3$)

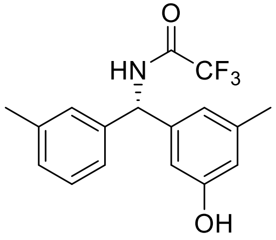

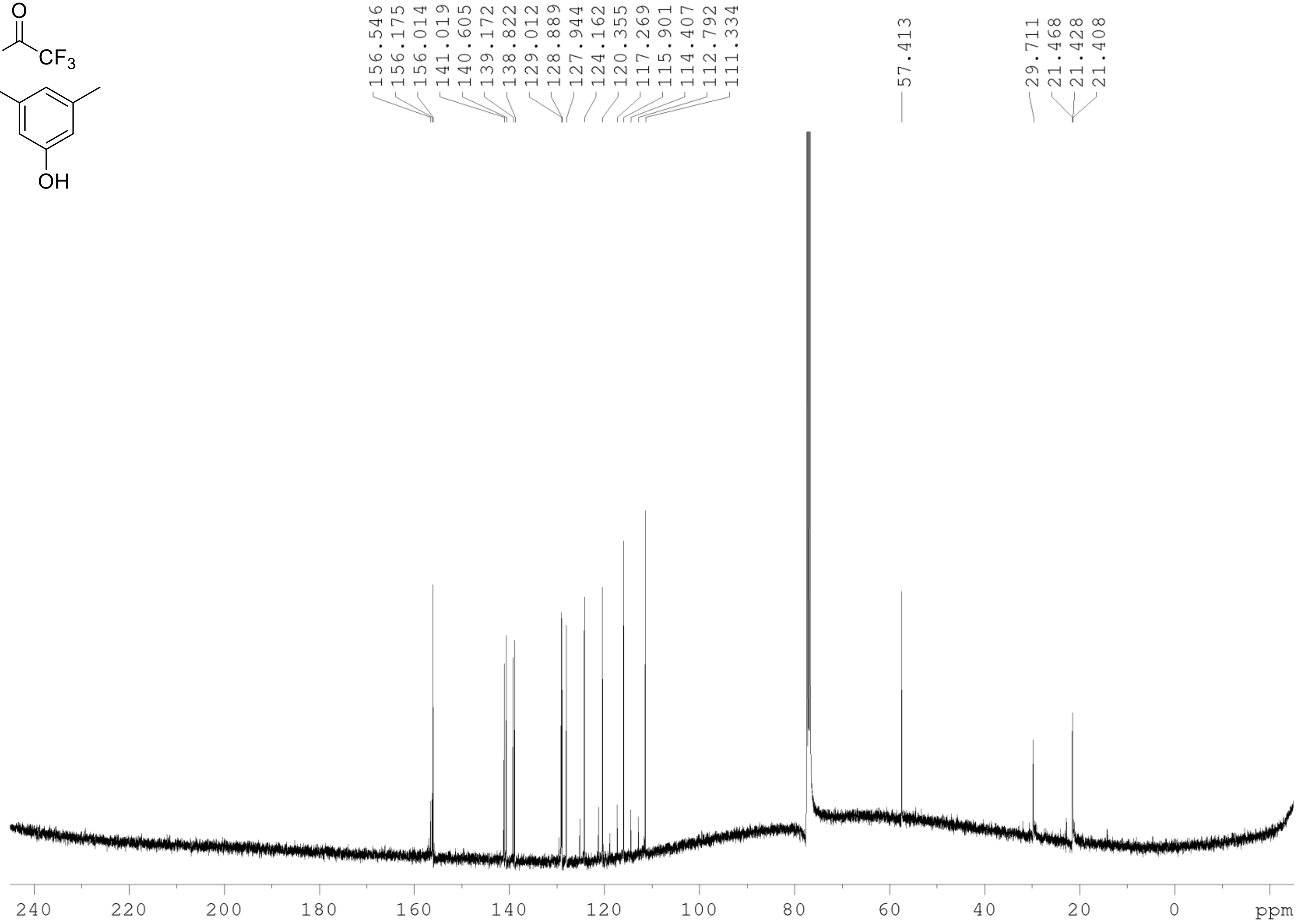

(S)-2,2,2-trifluoro-N-((3-hydroxy-5-methylphenyl)(m-tolyl)methyl)acetamide (3c) ^{19}F NMR (176 MHz, $CDCl_3$)

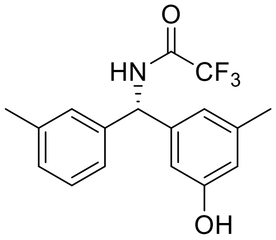

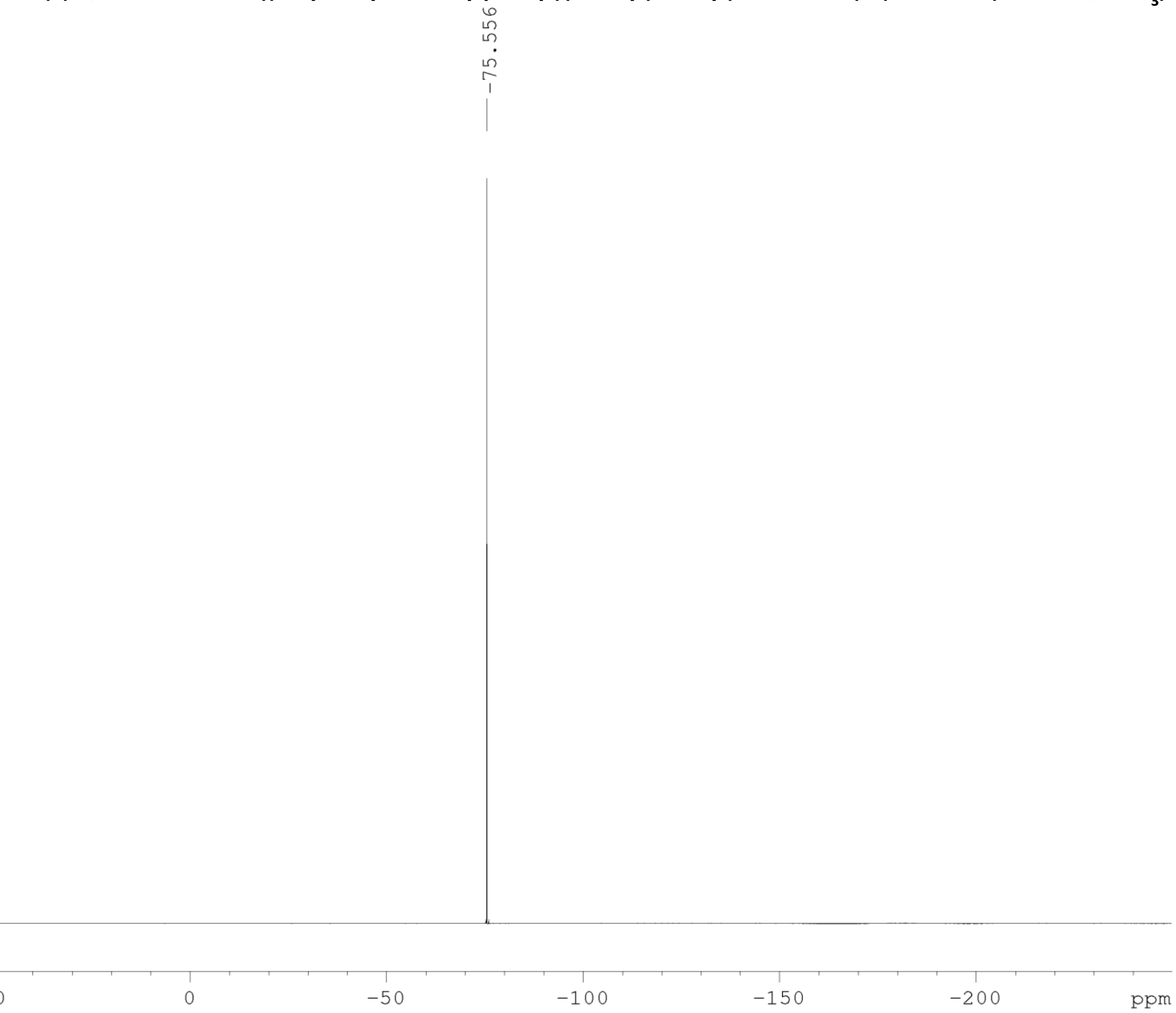

(*R*)-*P*-(3-chloro-5-hydroxyphenyl)-*P*-(3-chlorophenyl)-*N*-(4-methoxyphenyl)phosphinic amide (5) ^{1}H NMR (400 MHz, $CDCl_3$:MeOD)

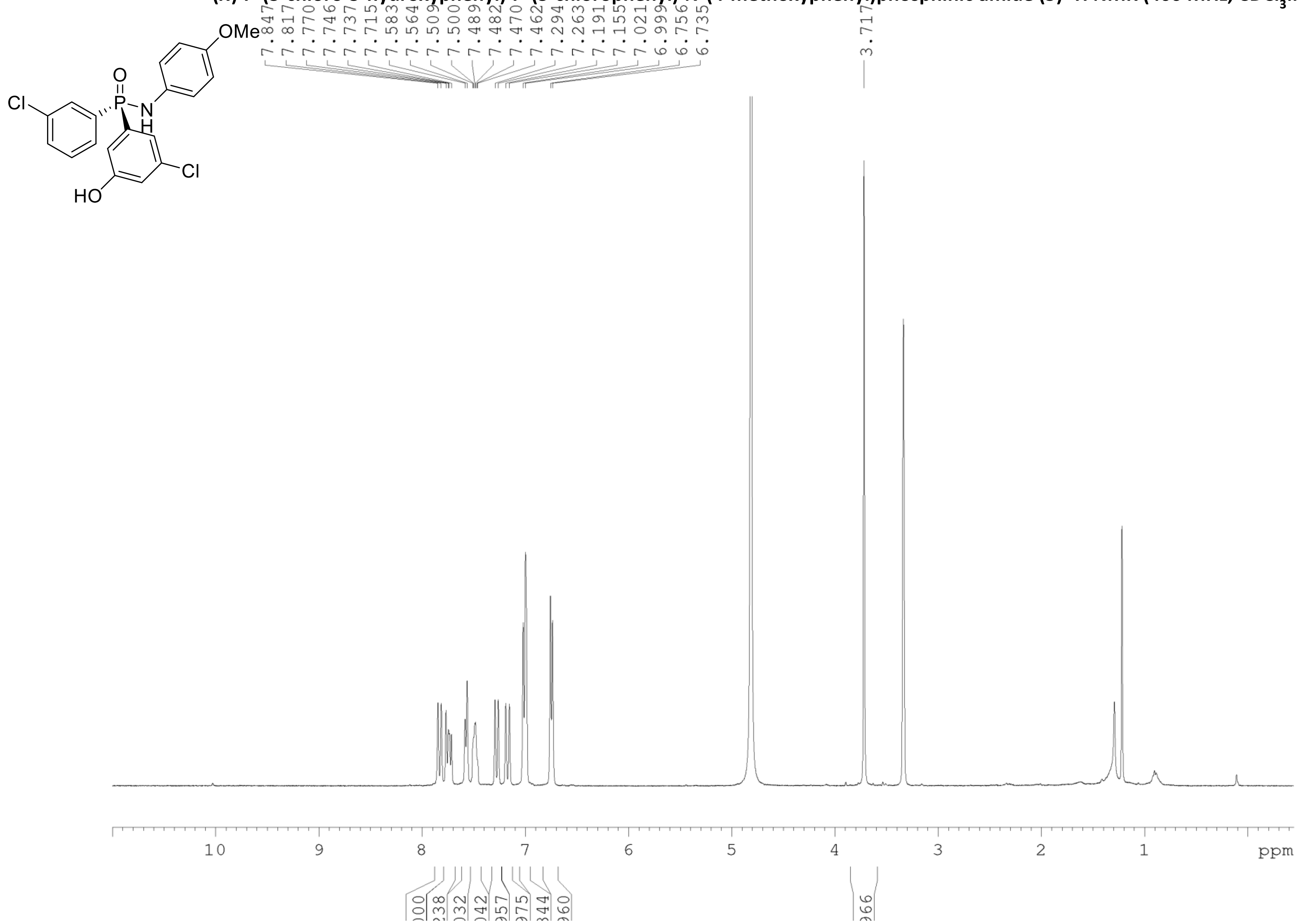

(*R*)-*P*-(3-chloro-5-hydroxyphenyl)-*P*-(3-chlorophenyl)-*N*-(4-methoxyphenyl)phosphinic amide (5) ^{13}C NMR (101 MHz, $CDCl_3$:MeOD)

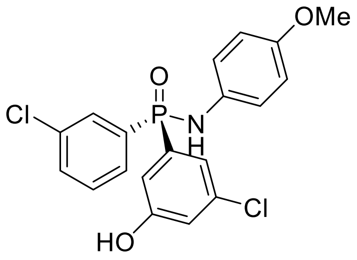

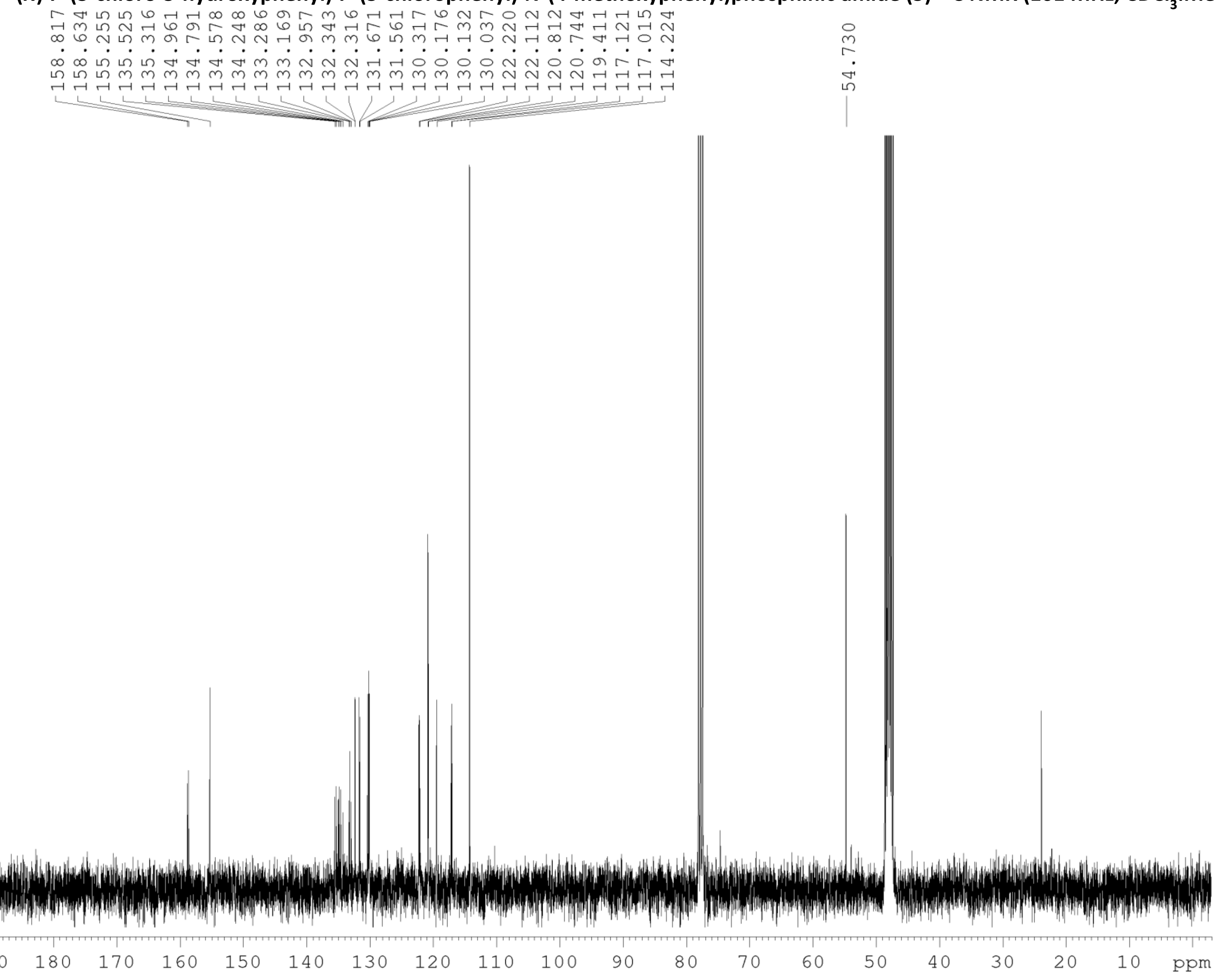

(*R*)-*P*-(3-chloro-5-hydroxyphenyl)-*P*-(3-chlorophenyl)-*N*-(4-methoxyphenyl)phosphinic amide (5) ^{31}P NMR (162 MHz, $CDCl_3$:MeOD)

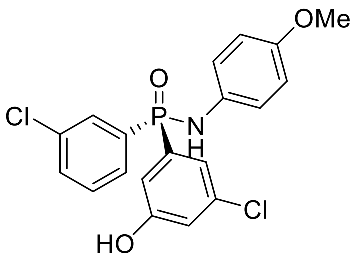

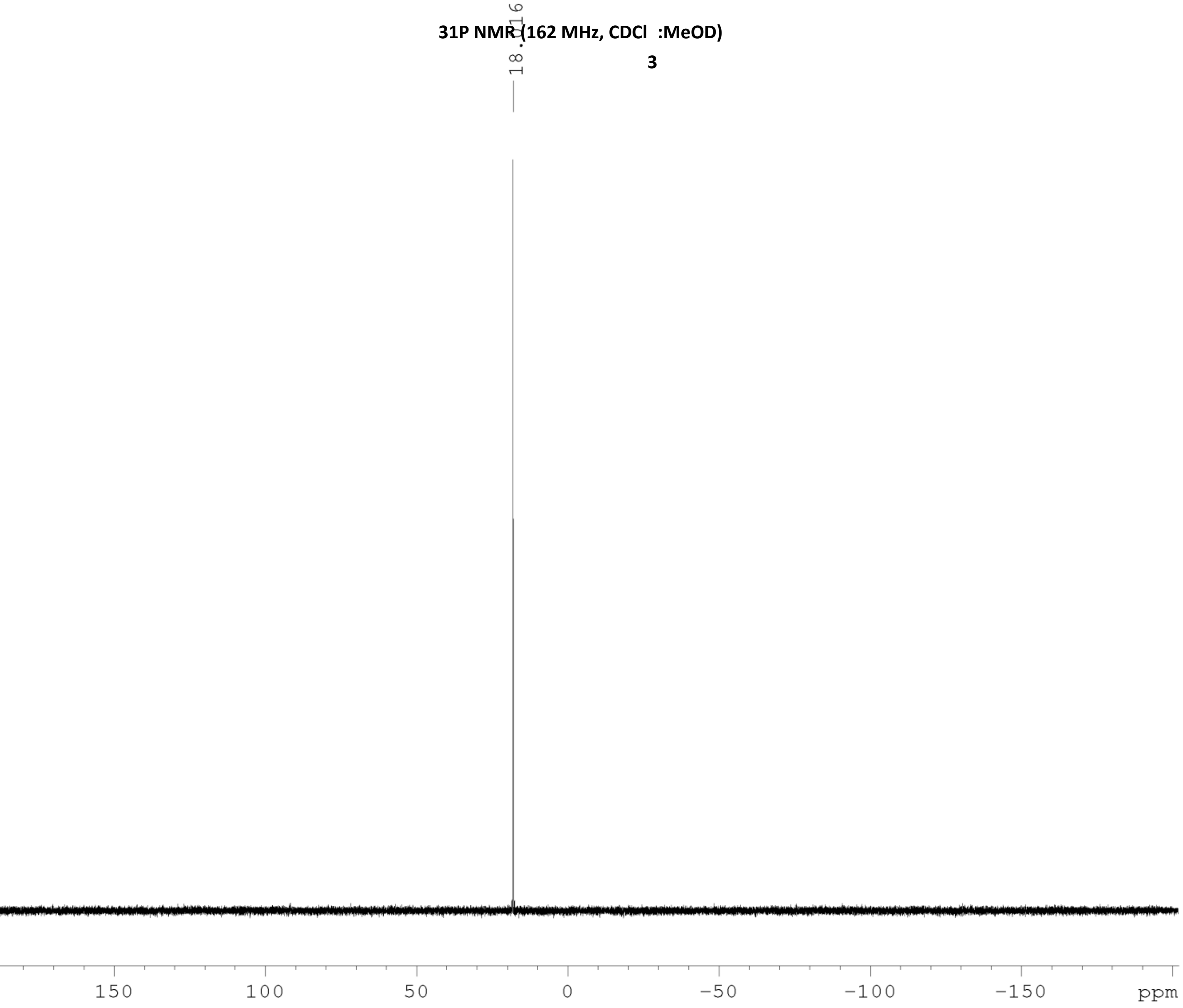

(3-chloro-5-hydroxyphenyl)(3-chlorophenyl)(4-methoxybenzyl)phosphine oxide (8) ^{1}H NMR (400 MHz, $CDCl_3$)

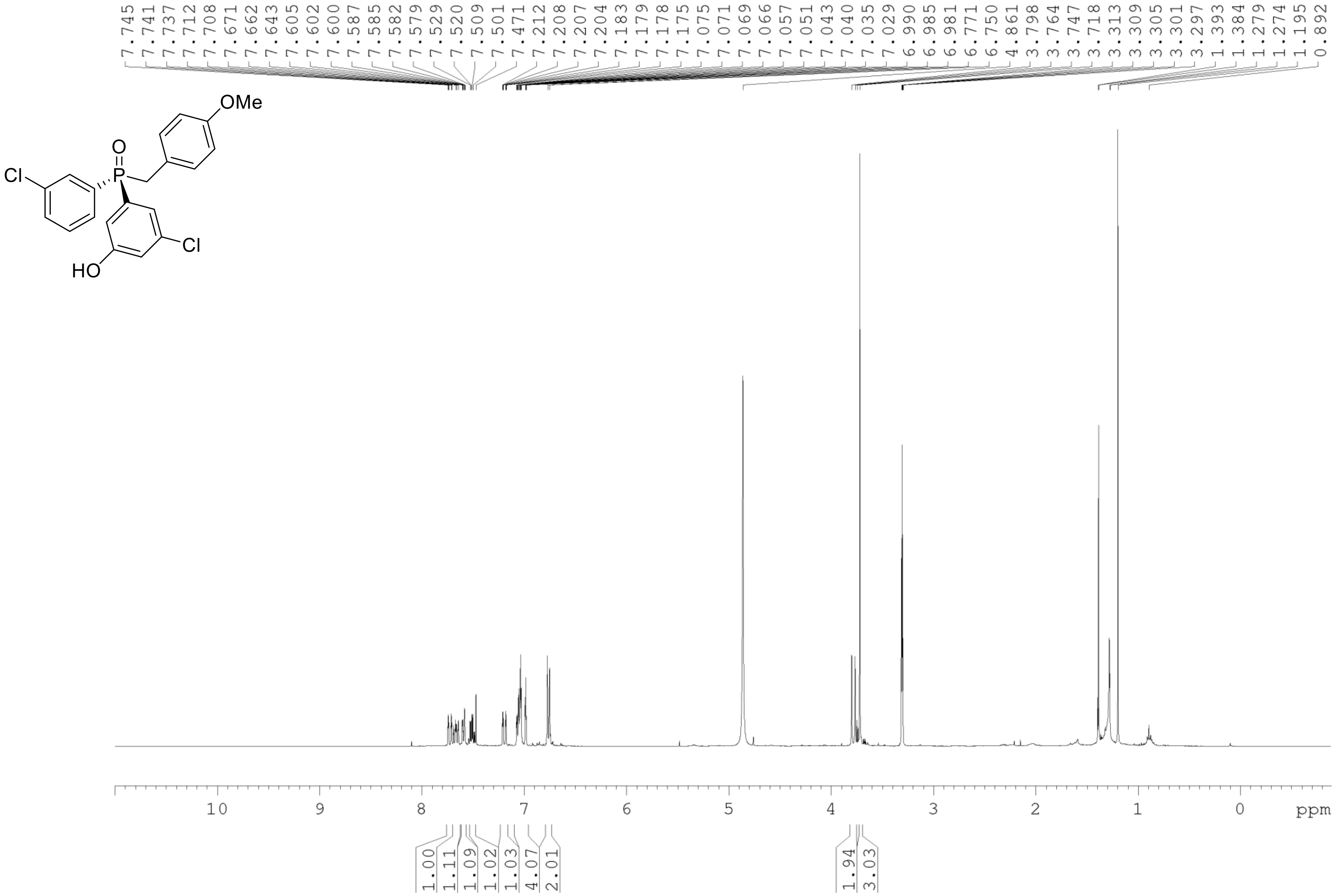

(3-chloro-5-hydroxyphenyl)(3-chlorophenyl)(4-methoxybenzyl)phosphine oxide (8) ^{13}**C NMR (101 MHz, $CDCl_3$)**

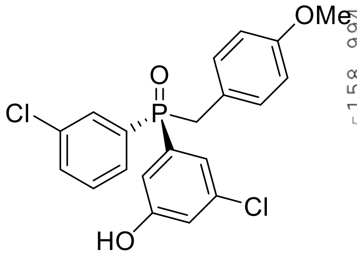

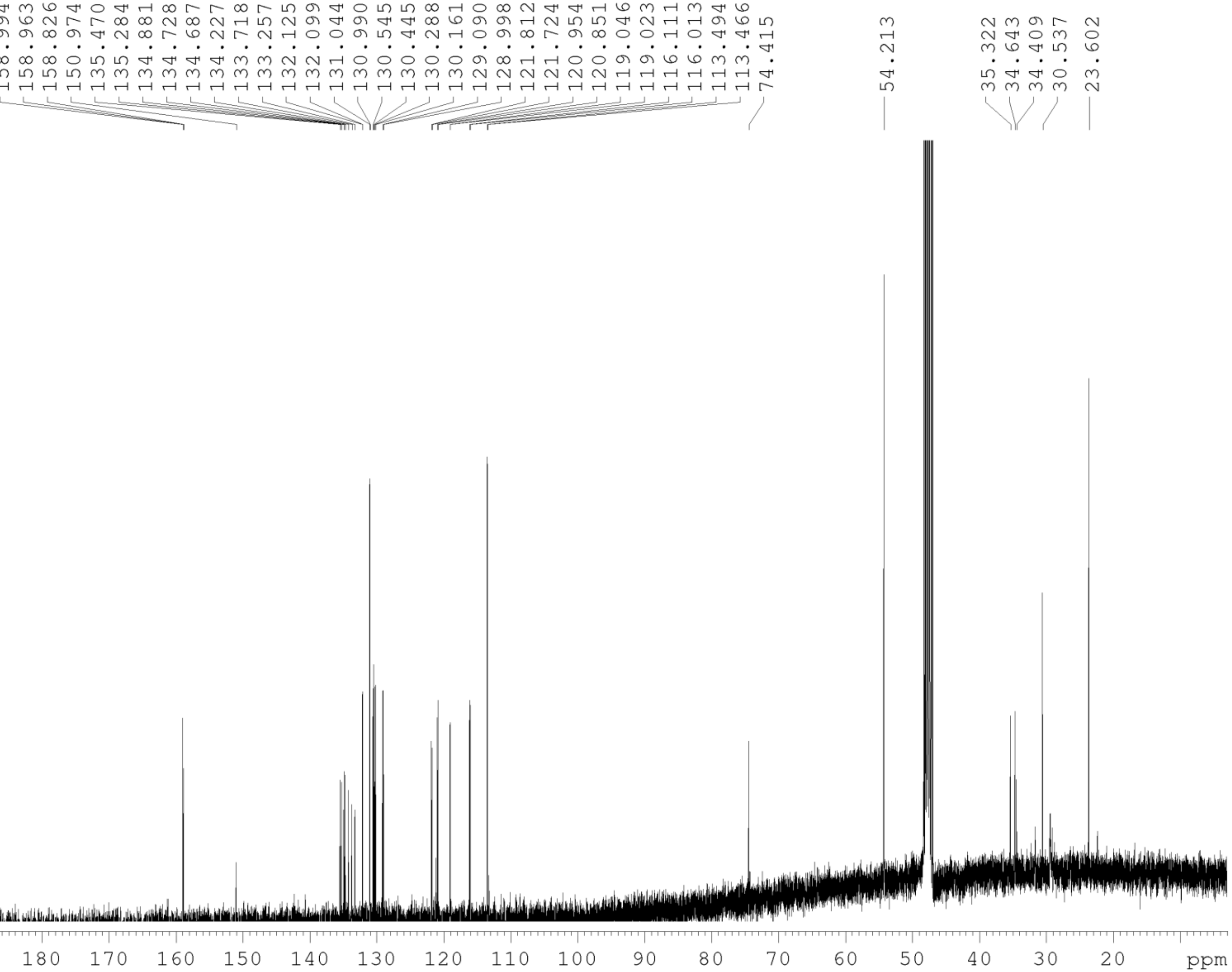

(3-chloro-5-hydroxyphenyl)(3-chlorophenyl)(4-methoxybenzyl)phosphine oxide (8) ^{31}P NMR (162 MHz, $CDCl_3$)

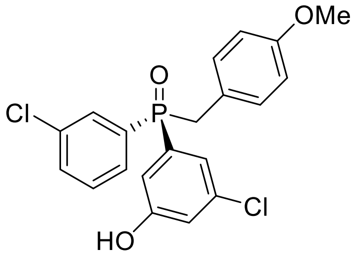

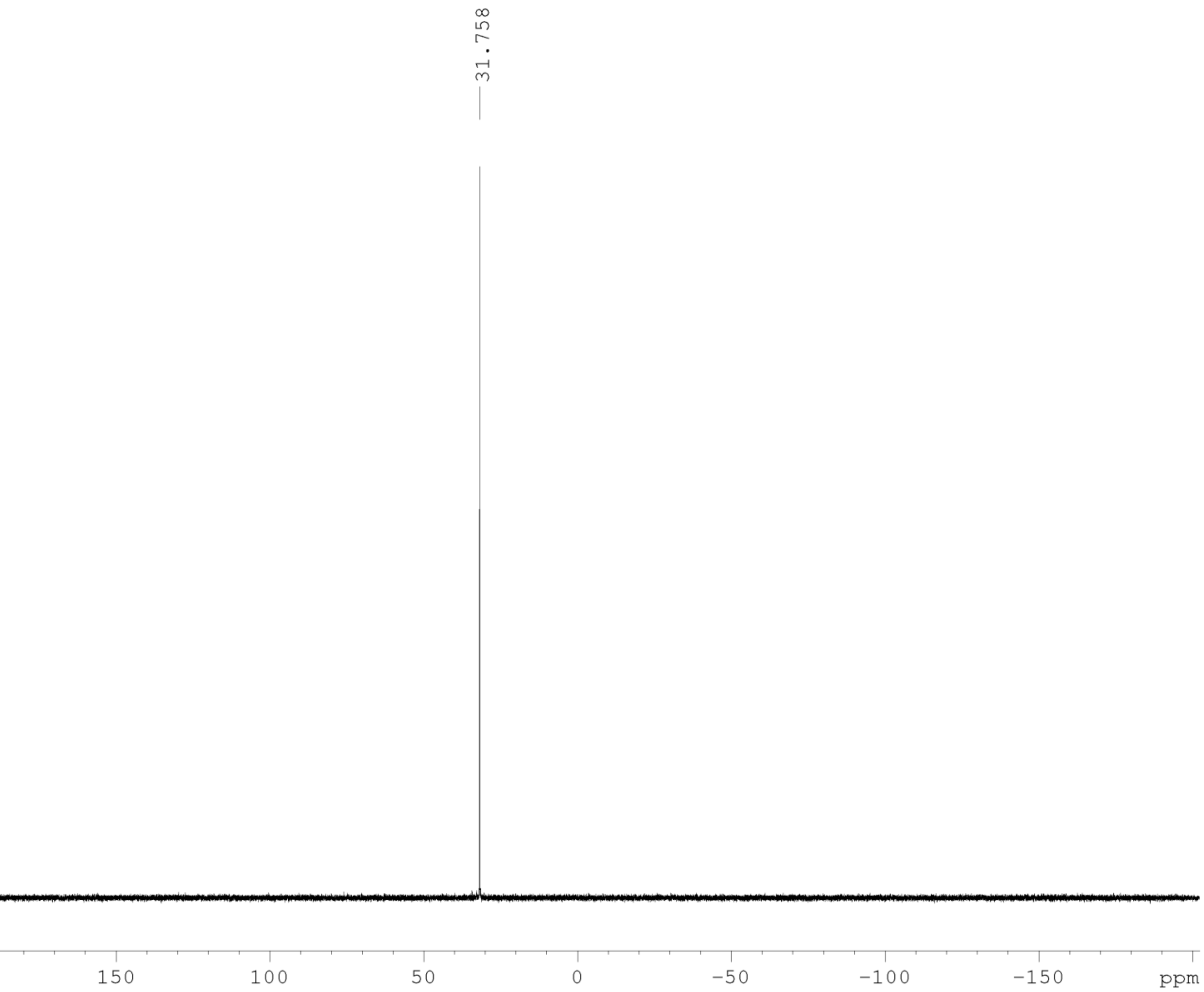

Computational Investigations

Computational methods

All conformational searches were performed using MacroModel (Version 12.3) in the gas phase utilizing the OPLS3 force field[4] and a mixture of Low Mode following and Monte Carlo search algorithms.[5] Quantum mechanical calculations were carried out using Gaussian 16, rev A.03[6] The molecular geometries were optimized using the B3LYP functional[7] with the 6-31G* basis set[8], with SDD basis set[9] on the iridium. The optimisations were done either in gas phase or using the implicit SMD solvent model.[10] All single-point energies were separately calculated using M06 functional[11] and def2-TZVP[12] basis set using the SMD solvent model. Frequency calculations were performed on all structures and confirmed to contain no imaginary frequencies or just one imaginary frequency for ground states and transition states, respectively. The free energies were corrected using quasi-harmonic approximation, corrections were done using GoodVibes script.[13] Full set of DFT output files with optimized structures, frequencies and high-level single-point energies are provided (see below for a full description of the dataset) and can be found at DOI: http://doi.org/10.17639/nott.7218

Additional Computational Figures

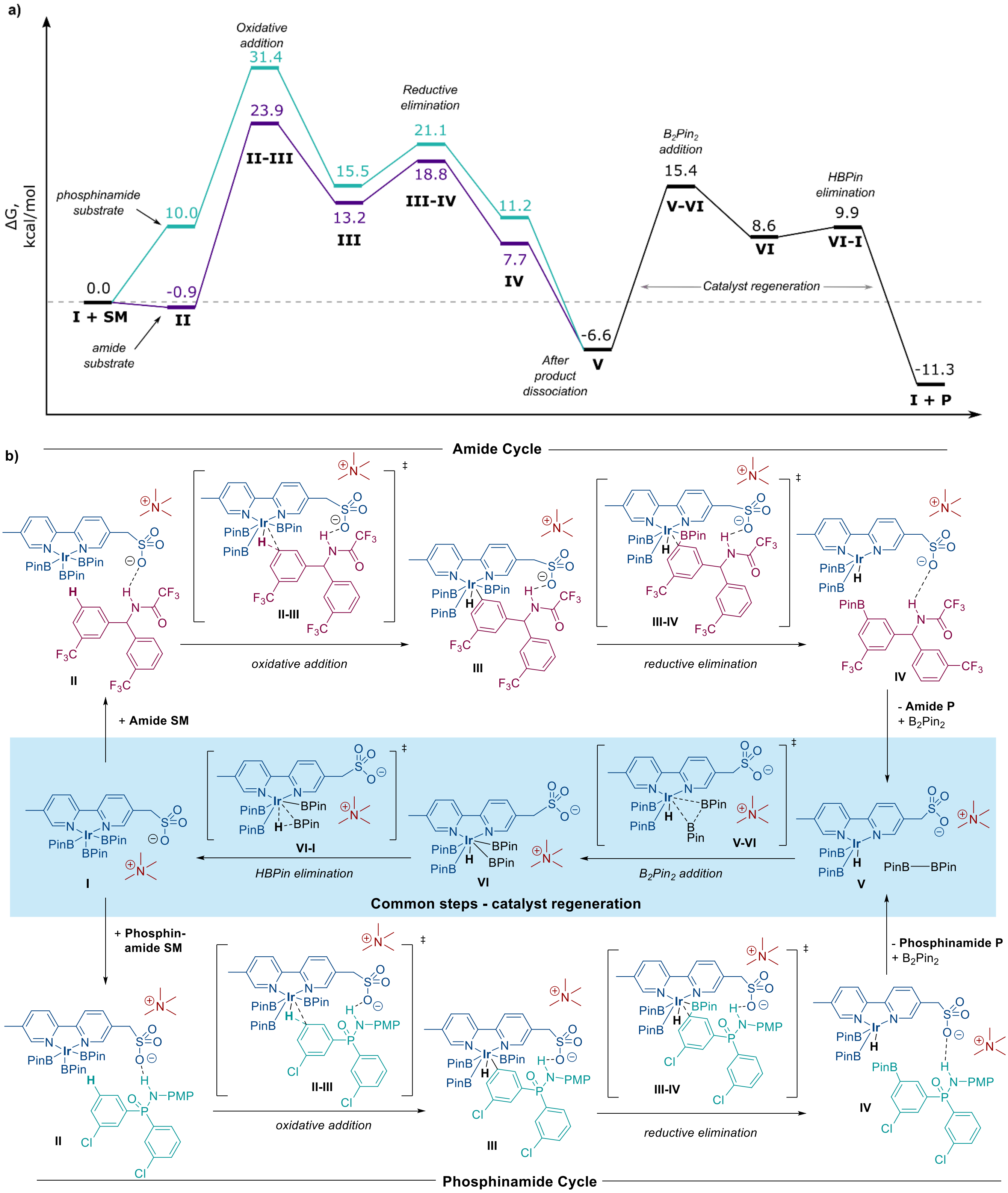

Figure S1. Model study of the Ir-catalyzed borylation pathway with amide substrate (magenta) and with phosphinamide substrate (cyan).

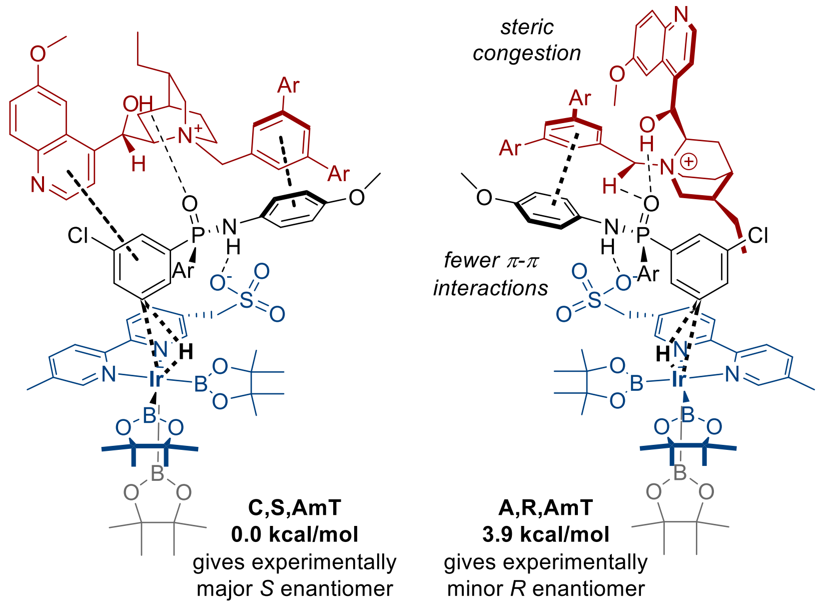

Figure S2. Lowest energy transition states leading to the experimentally major and minor phosphinamide product enantiomers using pseudoenantiomeric QD cation.

Dispersion-corrected geometry optimisation benchmark

A range of previously obtained low-energy gas phase geometries of the full oxidative addition transition states were reoptimized using r2SCAN-3c,[14] and their energies were calculated both with M06 and wB97M-V functionals.[15] r^2SCAN-3c and wB97M-V calculations were both done using ORCA v5.0.3.[16]

Table S1. Benchmark comparing transition state energies and predicted enantioselectivity obtained from B3LYP gas phase, B3LYP solvent phase, r2SCAN-3c gas phase, r2SCAN-3c solvent phase geometries and various single point energy corrections.

		Geometries	B3LYP/6-31G*+SDD/gas phase				B3LYP/6-31G*/SDD/SMD(diethyl ether)				r2SCAN-3c/gas phase			
		Single point corr.	M06/def2-TZVP/SMD(diethyl ether)				M06/def2-TZVP/SMD(diethyl ether)				M06/def2-TZVP/SMD(diethyl ether)			
bipy orient.	product	TS conformer	E, Eh	ΔE, kcal/mol	G, Eh	ΔG, kcal/mol	E, Eh	ΔE, kcal/mol	G, Eh	ΔG, kcal/mol	E, Eh	ΔE, kcal/mol	G, Eh	ΔG, kcal/mol
A	***R***	AR_AmA1	-6616.237684	**0.00**	-6614.194021	**0.09**	-6616.228043	0.97	-6614.184113	2.35	-6616.258127	0.63	-6614.233311	1.96
		AR7	-6616.237624	**0.04**	**-6614.194162**	**0.00**	-6616.229397	**0.12**	-6614.186693	**0.73**	**-6616.259123**	**0.00**	**-6614.236438**	**0.00**
		AR_AmT1	-6616.234477	2.01	-6614.191948	1.39	-6616.229164	**0.27**	-6614.186422	**0.90**	-6616.255118	2.51	-6614.233418	1.89
		AR9	-6616.234477	2.01	-6614.191948	1.39	-6616.229164	**0.27**	-6614.186428	**0.90**	-6616.255117	2.51	-6614.233437	1.88
C	***R***	CR_AmA44	-6616.230821	4.31	-6614.187533	4.16	-6616.22403	3.49	-6614.176717	6.99	-6616.249480	6.05	-6614.227138	5.84
		CR_AmT1	-6616.231484	3.89	-6614.1871	4.43	-6616.219894	6.09	-6614.177386	6.57	-6616.253916	3.27	-6614.229318	4.47
		CR11	-6616.230209	4.69	-6614.186206	4.99					-6616.255544	2.25	-6614.231080	3.36
A	***S***	AS_AmA2	-6616.23282	3.05	-6614.189902	2.67	-6616.229354	**0.15**	-6614.187705	**0.10**	-6616.252043	4.44	-6614.227350	5.70
		AS_AmT1	-6616.232142	3.48	-6614.190392	2.37	-6616.226404	2.00	-6614.184295	2.24	-6616.250449	5.44	-6614.226938	5.96
		AS_AmT11	-6616.232105	3.50	-6614.19017	2.51					-6616.251082	5.05	-6614.228745	4.83
C	***S***	CS_AmA3	-6616.235728	**1.23**	-6614.192324	**1.15**	-6616.229173	0.26	-6614.184515	2.10	-6616.258443	0.43	-6614.233770	1.67
		CS1	-6616.236292	**0.87**	-6614.193302	**0.54**	-6616.229593	**0.00**	-6614.187858	**0.00**	**-6616.258621**	**0.32**	**-6614.236208**	**0.14**
		CS_AmT4	-6616.227427	6.44	-6614.185044	5.72					-6616.251269	4.93	-6614.229377	4.43
**Predicted enantioselectivity (% ee)**			**-60.4**				**50.4**				**-14.9**			

Table S1 (*continued*). Benchmark comparing transition state energies and predicted enantioselectivity obtained from B3LYP gas phase, B3LYP solvent phase, r2SCAN-3c gas phase, r2SCAN-3c solvent phase geometries and various single point energy corrections.

		**Geometries**	**r2SCAN-3c/PCM(diethyl ether)**				**r2SCAN-3c/PCM(diethyl ether)**			
		**Single point corr.**	**M06/def2-TZVP/SMD(diethyl ether)**				**wB97M-V/def2tzvp/SMD(diethyl ether)**			
**bipy orient.**	**product**	**TS conformer**	E, Eh	ΔE, kcal/mol	G, Eh	ΔG, kcal/mol	E, Eh	ΔE, kcal/mol	G, Eh	ΔG, kcal/mol
A	***R***	AR_AmA1	-6616.257187	1.40	-6614.237444	3.29	**-6618.009302**	**0.00**	**-6615.989560**	**0.93**
		AR7	**-6616.259421**	**0.00**	**-6614.242685**	**0.00**	**-6618.007771**	**0.96**	**-6615.991035**	**0.00**
		AR_AmT1	-6616.253031	4.01	-6614.234178	5.34	-6618.002858	4.04	-6615.984005	4.41
		AR9	-6616.252961	4.05	-6614.234146	5.36	-6618.002912	4.01	-6615.984097	4.35
C	***R***	CR_AmA44	-6616.247808	7.29	-6614.22781	9.33	-6617.997790	7.22	-6615.977791	8.31
		CR_AmT1	-6616.252057	4.62	-6614.233134	5.99	-6618.001987	4.59	-6615.983063	5.00
		CR11	-6616.253967	3.42	-6614.234644	5.05	-6618.000936	5.25	-6615.981613	5.91
A	***S***	AS_AmA2	-6616.25127	5.11	-6614.233915	5.50	-6618.002826	4.06	-6615.985471	3.49
		AS_AmT1	-6616.250864	5.37	-6614.233798	5.58	-6618.000515	5.51	-6615.983450	4.76
		AS_AmT11	-6616.249614	6.15	-6614.231395	7.08	-6617.997161	7.62	-6615.978942	7.59
C	***S***	CS_AmA3	**-6616.256569**	**1.79**	**-6614.235891**	**4.26**	**-6618.007942**	**0.85**	**-6615.987265**	**2.37**
		CS1	-6616.255621	2.38	-6614.235895	4.26	**-6618.007446**	**1.16**	**-6615.98772**	**2.08**
		CS_AmT4	-6616.249066	6.50	-6614.230893	7.40	-6617.99842	6.83	-6615.980246	6.77
**Predicted enantioselectivity (% ee)**			**-99.9**				**-94.9**			

3D geometry depictions and non-covalent interaction plots for lowest energy full system transition states

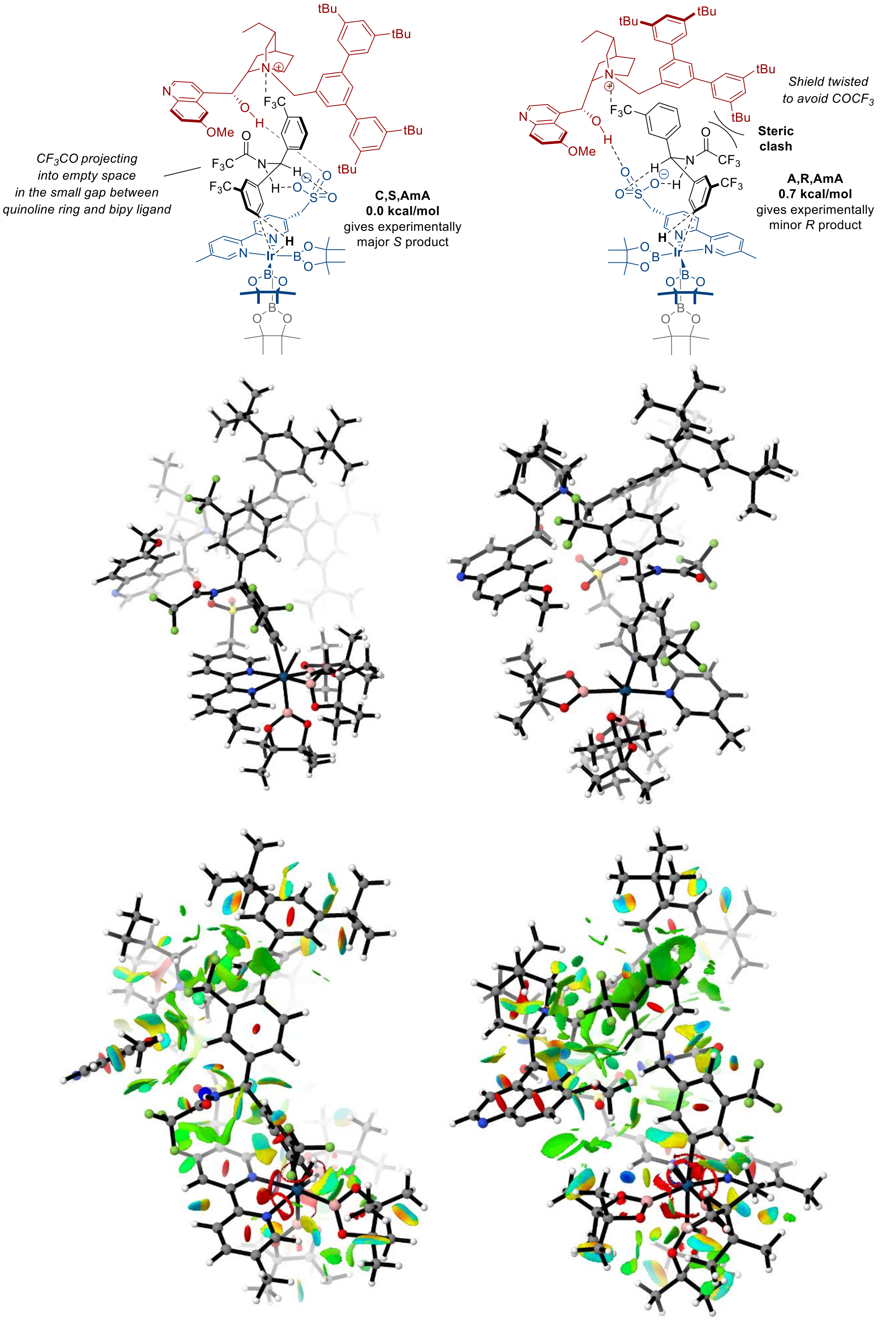

Figure S3: 3D depictions and NCI plots for the B3LYP/6-31G*-SDD/SMD(diethylether) optimized oxidative addition transition state geometries for the full system with amide substrate and dihydroquinine derived chiral cation.

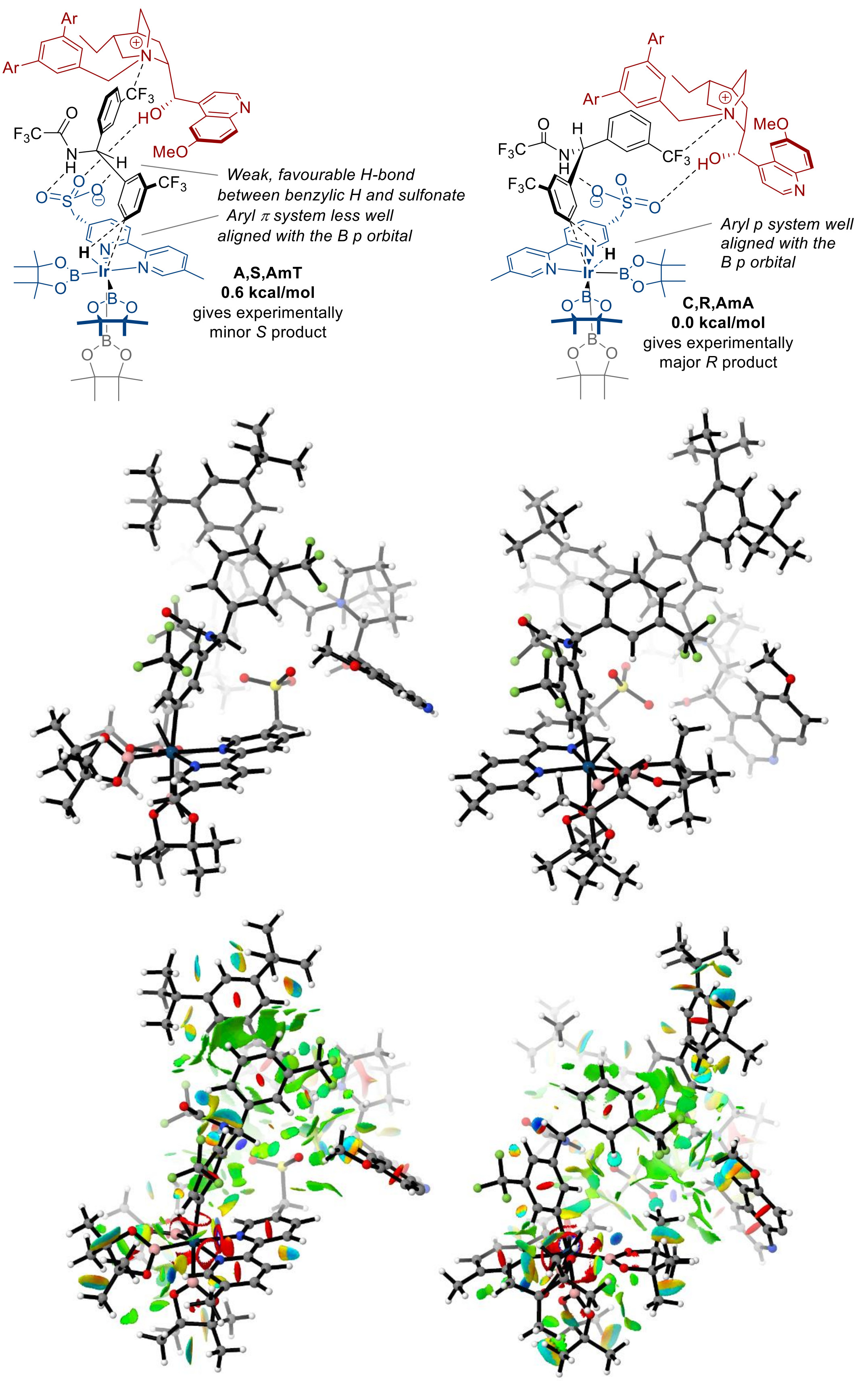

Figure S4: 3D depictions and NCI plots for the B3LYP/6-31G*-SDD/SMD(diethylether) optimized oxidative addition transition state geometries for the full system with amide substrate and the pseudoenantiomeric dihydroquinidine derived chiral cation.

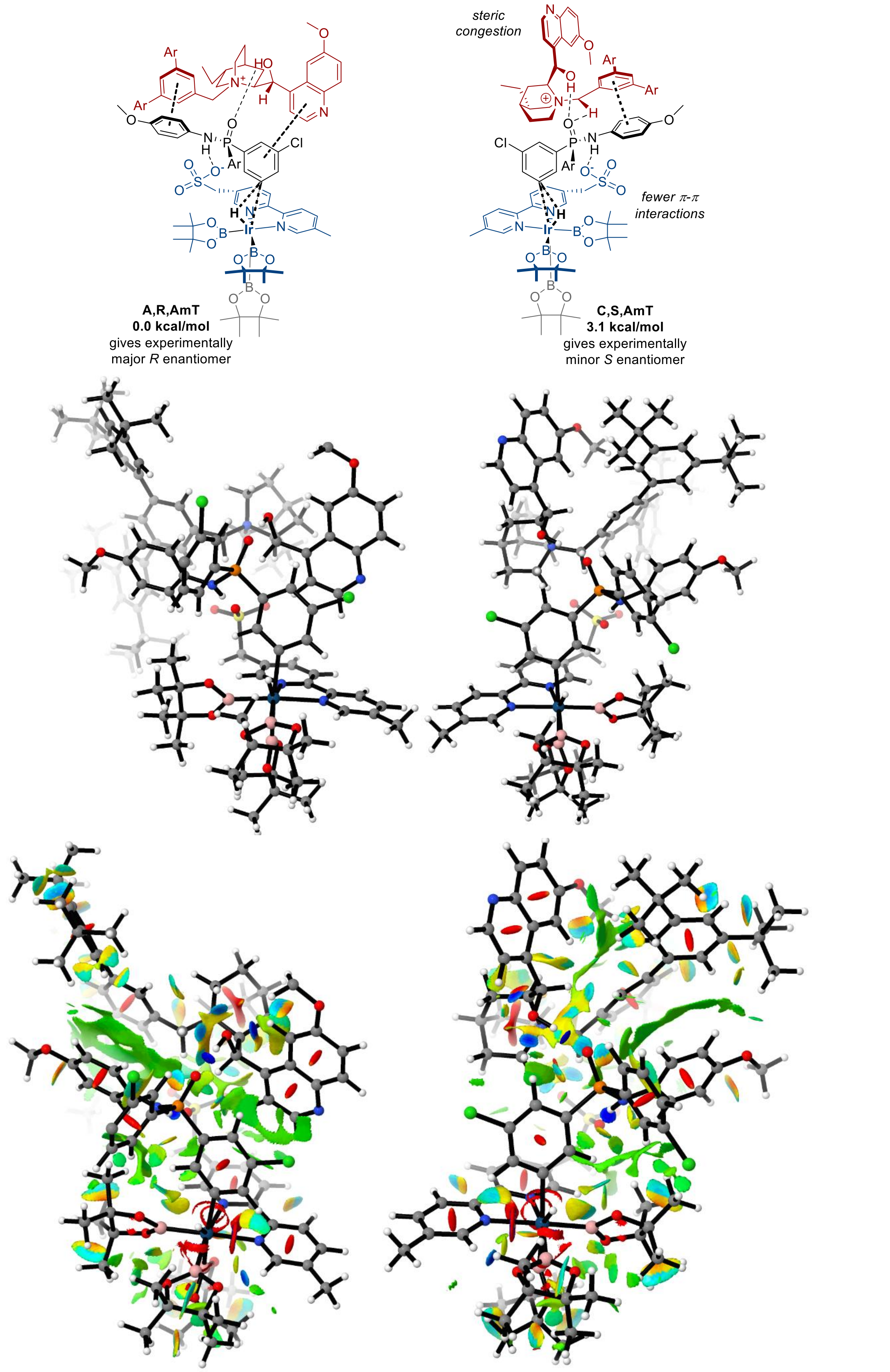

Figure S5: 3D depictions and NCI plots for the B3LYP/6-31G*-SDD/SMD(diethylether) optimized oxidative addition transition state geometries for the full system with phopshinamide substrate and dihydroquinine derived chiral cation.

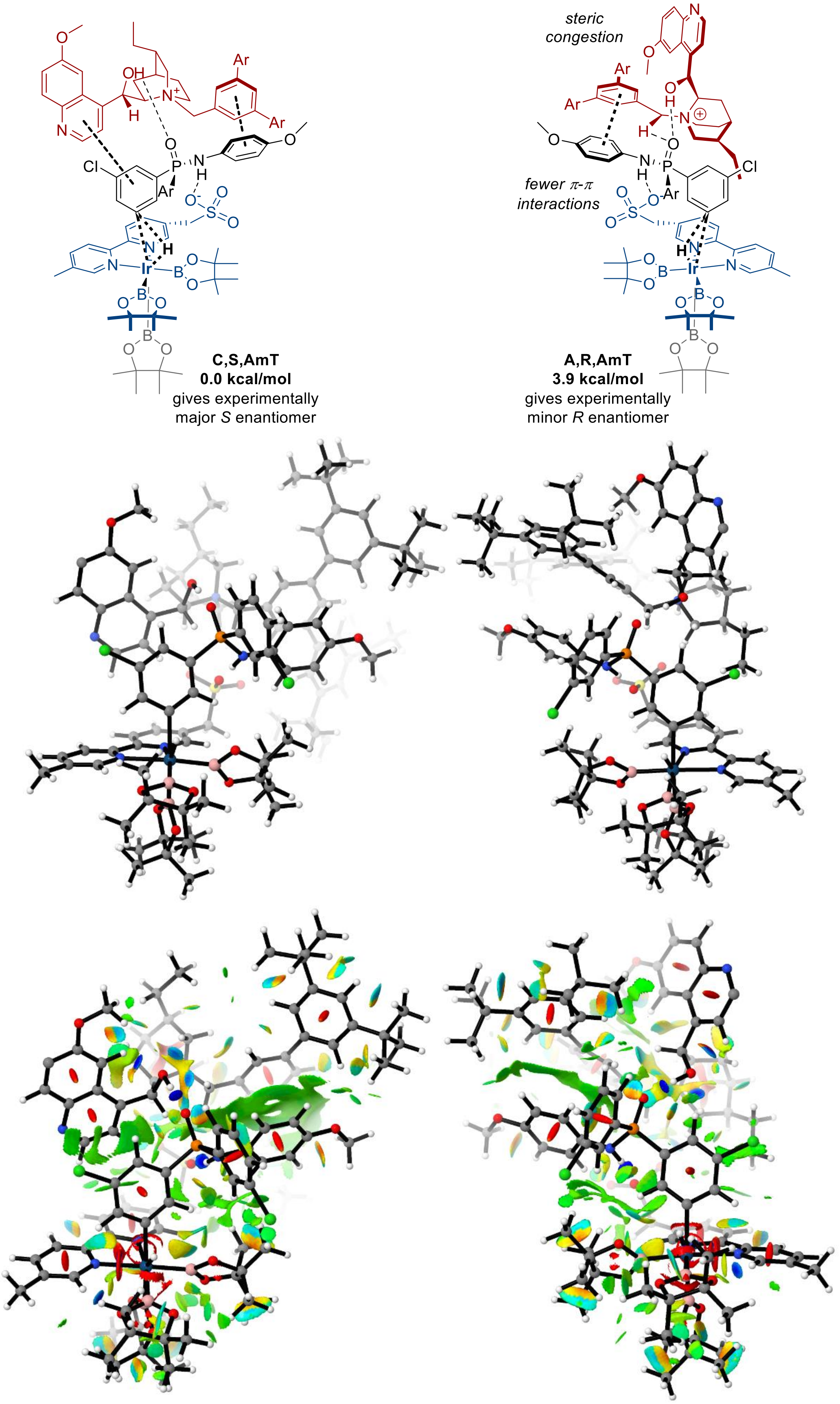

Figure S6: 3D depictions and NCI plots for the B3LYP/6-31G*-SDD/SMD(diethylether) optimized oxidative addition transition state geometries for the full system with phopshinamide substrate and the pseudoenantiomeric dihydroquinidine derived chiral cation.

Summary of the associated computational dataset contents

This dataset contains Gaussian DFT output files of the key ground-states and transition state DFT optimized structures. It is available in University of Nottingham repository at DOI: http://doi.org/10.17639/nott.7218

The dataset contains 1693 files in total and is organised in one archive containing 71 separate folders – the full structure of the archive is shown below.

There are twelve top level folders, corresponding to computational data for studies depicted in Figures 2, 3, 5, 6, 7, 8, 9, 10, 11 and S2 in the main paper and this SI, respectively.

Folders "Fig7_amideQ_full_study", "Fig8_amideQD_full_study", "Fig11_phosphinamideQ_full_study", "FigS2_phosphinamideQD_full_study" contain data for the full 260-atom system studies. Each of these folders contain "Gas_geometries" and "Solvent_geometries" subfolders, containing the oxidative addition transition state geometries optimized in gas phase or using SMD(diethylether) solvent model, respectively. The transition states in these subfolders are organized by the diastereoisomer of the transition state - AR, AS, CR and CS. Please see the main text for fuller explanation of this nomenclature.

Each of the lower level folders contain the output of a frequency calculation at B3LYP/6-31g*/SDD level in gas phase or with SMD(diethylether) solvent model (*freq001.out files), as well as single point calculation at M06/def2-TZVP/SMD(diethylether) level (*m06tzvp001.out files). All optimized geometries are also provided as *.sdf files for even better usability.

All of the files can be opened in any text editor. Gaussian output structures can be viewed and the frequency modes visualised in GausView, Avogadro, jmol and in most other molecular viewers/editors. *.sdf files can be viewed in essentially all 3D molecular editors and viewers.

```
├── DataReadme.txt
├── Fig8_amideQD_full_study
│   ├── Gas_geometries
│   │   ├── AR
│   │   │   ├── AR17Cfreq001.out
│   │   │   ├── AR17Cm06tzvp001.out
│   │   │   ├── AR17Cm06tzvp001.sdf
│   │   │   ├── AR18Cfreq001.out
│   │   │   ├── AR18Cm06tzvp001.out
│   │   │   ├── AR18Cm06tzvp001.sdf
│   │   │   ├── AR19Cfreq001.out
│   │   │   ├── AR19Cm06tzvp001.out
│   │   │   ├── AR19Cm06tzvp001.sdf
│   │   │   ├── AR1Cfreq001.out
│   │   │   ├── AR1Cm06tzvp001.out
│   │   │   ├── AR1Cm06tzvp001.sdf
│   │   │   ├── AR21Cfreq001.out
│   │   │   ├── AR21Cm06tzvp001.out
│   │   │   ├── AR21Cm06tzvp001.sdf
│   │   │   ├── AR22Cfreq001.out
│   │   │   ├── AR22Cm06tzvp001.out
│   │   │   ├── AR22Cm06tzvp001.sdf
│   │   │   ├── AR23Cfreq001.out
│   │   │   ├── AR23Cm06tzvp001.out
│   │   │   ├── AR23Cm06tzvp001.sdf
│   │   │   ├── AR2Cfreq001.out
│   │   │   ├── AR2Cm06tzvp001.out
│   │   │   ├── AR2Cm06tzvp001.sdf
│   │   │   ├── AR33Cfreq001.out
│   │   │   ├── AR33Cm06tzvp001.out
```

```
│   │   │   ├── AR33Cm06tzvp001.sdf
│   │   │   ├── AR81Cfreq001.out
│   │   │   ├── AR81Cm06tzvp001.out
│   │   │   ├── AR81Cm06tzvp001.sdf
│   │   │   ├── AR82Cfreq001.out
│   │   │   ├── AR82Cm06tzvp001.out
│   │   │   ├── AR82Cm06tzvp001.sdf
│   │   │   ├── AR_AmA125Efreq001.out
│   │   │   ├── AR_AmA125Em06tzvp001.out
│   │   │   ├── AR_AmA125Em06tzvp001.sdf
│   │   │   ├── AR_AmA1Efreq001.out
│   │   │   ├── AR_AmA1Em06tzvp001.out
│   │   │   ├── AR_AmA1Em06tzvp001.sdf
│   │   │   ├── AR_AmA210Efreq001.out
│   │   │   ├── AR_AmA210Em06tzvp001.out
│   │   │   ├── AR_AmA210Em06tzvp001.sdf
│   │   │   ├── AR_AmA21Efreq001.out
│   │   │   ├── AR_AmA21Em06tzvp001.out
│   │   │   ├── AR_AmA21Em06tzvp001.sdf
│   │   │   ├── AR_AmA3Efreq001.out
│   │   │   ├── AR_AmA3Em06tzvp001.out
│   │   │   ├── AR_AmA3Em06tzvp001.sdf
│   │   │   ├── AR_AmA865Efreq001.out
│   │   │   ├── AR_AmA865Em06tzvp001.out
│   │   │   ├── AR_AmA865Em06tzvp001.sdf
│   │   │   ├── AR_AmA911Efreq001.out
│   │   │   ├── AR_AmA911Em06tzvp001.out
│   │   │   ├── AR_AmA911Em06tzvp001.sdf
│   │   │   ├── AR_AmT1Cfreq001.out
│   │   │   ├── AR_AmT1Cm06tzvp001.out
│   │   │   ├── AR_AmT1Cm06tzvp001.sdf
│   │   │   ├── AR_AmT27Cfreq001.out
│   │   │   ├── AR_AmT27Cm06tzvp001.out
│   │   │   ├── AR_AmT27Cm06tzvp001.sdf
│   │   │   ├── AR_AmT365Cfreq001.out
│   │   │   ├── AR_AmT365Cm06tzvp001.out
│   │   │   ├── AR_AmT365Cm06tzvp001.sdf
│   │   │   ├── AR_AmT417Cfreq001.out
│   │   │   ├── AR_AmT417Cm06tzvp001.out
│   │   │   ├── AR_AmT417Cm06tzvp001.sdf
│   │   │   ├── AR_AmT463Cfreq001.out
│   │   │   ├── AR_AmT463Cm06tzvp001.out
│   │   │   ├── AR_AmT463Cm06tzvp001.sdf
│   │   │   ├── AR_AmT48Cfreq001.out
│   │   │   ├── AR_AmT48Cm06tzvp001.out
│   │   │   ├── AR_AmT48Cm06tzvp001.sdf
│   │   │   ├── AR_AmT523Cfreq001.out
│   │   │   ├── AR_AmT523Cm06tzvp001.out
│   │   │   └── AR_AmT523Cm06tzvp001.sdf
│   │   ├── AS
│   │   │   ├── AS10Efreq001.out
│   │   │   ├── AS10Em06tzvp001.out
│   │   │   ├── AS10Em06tzvp001.sdf
│   │   │   ├── AS11Bfreq001.out
│   │   │   ├── AS11Bm06tzvp001.out
│   │   │   ├── AS11Bm06tzvp001.sdf
│   │   │   ├── AS14Efreq001.out
│   │   │   ├── AS14Em06tzvp001.out
│   │   │   ├── AS14Em06tzvp001.sdf
```

```
│   │   │   ├── AS164Efreq001.out
│   │   │   ├── AS164Em06tzvp001.out
│   │   │   ├── AS164Em06tzvp001.sdf
│   │   │   ├── AS165Efreq001.out
│   │   │   ├── AS165Em06tzvp001.out
│   │   │   ├── AS165Em06tzvp001.sdf
│   │   │   ├── AS1Efreq001.out
│   │   │   ├── AS1Em06tzvp001.out
│   │   │   ├── AS1Em06tzvp001.sdf
│   │   │   ├── AS2Efreq001.out
│   │   │   ├── AS2Em06tzvp001.out
│   │   │   ├── AS2Em06tzvp001.sdf
│   │   │   ├── AS59Efreq001.out
│   │   │   ├── AS59Em06tzvp001.out
│   │   │   ├── AS59Em06tzvp001.sdf
│   │   │   ├── AS5Efreq001.out
│   │   │   ├── AS5Em06tzvp001.out
│   │   │   ├── AS5Em06tzvp001.sdf
│   │   │   ├── AS60Efreq001.out
│   │   │   ├── AS60Em06tzvp001.out
│   │   │   ├── AS60Em06tzvp001.sdf
│   │   │   ├── AS_AmA11Cfreq001.out
│   │   │   ├── AS_AmA11Cm06tzvp001.out
│   │   │   ├── AS_AmA11Cm06tzvp001.sdf
│   │   │   ├── AS_AmA1Cfreq001.out
│   │   │   ├── AS_AmA1Cm06tzvp001.out
│   │   │   ├── AS_AmA1Cm06tzvp001.sdf
│   │   │   ├── AS_AmA294Cfreq001.out
│   │   │   ├── AS_AmA294Cm06tzvp001.out
│   │   │   ├── AS_AmA294Cm06tzvp001.sdf
│   │   │   ├── AS_AmA2Cfreq001.out
│   │   │   ├── AS_AmA2Cm06tzvp001.out
│   │   │   ├── AS_AmA2Cm06tzvp001.sdf
│   │   │   ├── AS_AmT1Cfreq001.out
│   │   │   ├── AS_AmT1Cm06tzvp001.out
│   │   │   ├── AS_AmT1Cm06tzvp001.sdf
│   │   │   ├── AS_AmT294Cfreq001.out
│   │   │   ├── AS_AmT294Cm06tzvp001.out
│   │   │   ├── AS_AmT294Cm06tzvp001.sdf
│   │   │   ├── AS_AmT310Cfreq001.out
│   │   │   ├── AS_AmT310Cm06tzvp001.out
│   │   │   ├── AS_AmT310Cm06tzvp001.sdf
│   │   │   ├── AS_AmT492Cfreq001.out
│   │   │   ├── AS_AmT492Cm06tzvp001.out
│   │   │   ├── AS_AmT492Cm06tzvp001.sdf
│   │   │   ├── AS_AmT76Cfreq001.out
│   │   │   ├── AS_AmT76Cm06tzvp001.out
│   │   │   ├── AS_AmT76Cm06tzvp001.sdf
│   │   │   ├── AS_AmT79Cfreq001.out
│   │   │   ├── AS_AmT79Cm06tzvp001.out
│   │   │   └── AS_AmT79Cm06tzvp001.sdf
│   │   ├── CR
│   │   │   ├── CR106Bfreq001.out
│   │   │   ├── CR106Bm06tzvp001.out
│   │   │   ├── CR106Bm06tzvp001.sdf
│   │   │   ├── CR107Bfreq001.out
│   │   │   ├── CR107Bm06tzvp001.out
│   │   │   ├── CR107Bm06tzvp001.sdf
│   │   │   ├── CR131Bfreq001.out
```

```
│   │   │   ├── CR131Bm06tzvp001.out
│   │   │   ├── CR131Bm06tzvp001.sdf
│   │   │   ├── CR132Cfreq001.out
│   │   │   ├── CR132Cm06tzvp001.out
│   │   │   ├── CR132Cm06tzvp001.sdf
│   │   │   ├── CR1Bfreq001.out
│   │   │   ├── CR1Bm06tzvp001.out
│   │   │   ├── CR1Bm06tzvp001.sdf
│   │   │   ├── CR2Bfreq001.out
│   │   │   ├── CR2Bm06tzvp001.out
│   │   │   ├── CR2Bm06tzvp001.sdf
│   │   │   ├── CR3Cfreq001.out
│   │   │   ├── CR3Cm06tzvp001.out
│   │   │   ├── CR3Cm06tzvp001.sdf
│   │   │   ├── CR4Bfreq001.out
│   │   │   ├── CR4Bm06tzvp001.out
│   │   │   ├── CR4Bm06tzvp001.sdf
│   │   │   ├── CR_AmA127Cfreq001.out
│   │   │   ├── CR_AmA127Cm06tzvp001.out
│   │   │   ├── CR_AmA127Cm06tzvp001.sdf
│   │   │   ├── CR_AmA1Cfreq001.out
│   │   │   ├── CR_AmA1Cm06tzvp001.out
│   │   │   ├── CR_AmA1Cm06tzvp001.sdf
│   │   │   ├── CR_AmA58Cfreq001.out
│   │   │   ├── CR_AmA58Cm06tzvp001.out
│   │   │   ├── CR_AmA58Cm06tzvp001.sdf
│   │   │   ├── CR_AmA5Cfreq001.out
│   │   │   ├── CR_AmA5Cm06tzvp001.out
│   │   │   ├── CR_AmA5Cm06tzvp001.sdf
│   │   │   ├── CR_AmA7Cfreq001.out
│   │   │   ├── CR_AmA7Cm06tzvp001.out
│   │   │   ├── CR_AmA7Cm06tzvp001.sdf
│   │   │   ├── CR_AmT196Bfreq001.out
│   │   │   ├── CR_AmT196Bm06tzvp001.out
│   │   │   ├── CR_AmT196Bm06tzvp001.sdf
│   │   │   ├── CR_AmT1Bfreq001.out
│   │   │   ├── CR_AmT1Bm06tzvp001.out
│   │   │   └── CR_AmT1Bm06tzvp001.sdf
│   │   ├── CS
│   │   │   ├── CS10Efreq001.out
│   │   │   ├── CS10Em06tzvp001.out
│   │   │   ├── CS10Em06tzvp001.sdf
│   │   │   ├── CS118Efreq001.out
│   │   │   ├── CS118Em06tzvp001.out
│   │   │   ├── CS118Em06tzvp001.sdf
│   │   │   ├── CS119Efreq001.out
│   │   │   ├── CS119Em06tzvp001.out
│   │   │   ├── CS119Em06tzvp001.sdf
│   │   │   ├── CS124Efreq001.out
│   │   │   ├── CS124Em06tzvp001.out
│   │   │   ├── CS124Em06tzvp001.sdf
│   │   │   ├── CS125Efreq001.out
│   │   │   ├── CS125Em06tzvp001.out
│   │   │   ├── CS125Em06tzvp001.sdf
│   │   │   ├── CS131Efreq001.out
│   │   │   ├── CS131Em06tzvp001.out
│   │   │   ├── CS131Em06tzvp001.sdf
│   │   │   ├── CS132Efreq001.out
│   │   │   ├── CS132Em06tzvp001.out
```

```
│   │   │   ├── CS132Em06tzvp001.sdf
│   │   │   ├── CS180Efreq001.out
│   │   │   ├── CS180Em06tzvp001.out
│   │   │   ├── CS180Em06tzvp001.sdf
│   │   │   ├── CS1Efreq001.out
│   │   │   ├── CS1Em06tzvp001.out
│   │   │   ├── CS1Em06tzvp001.sdf
│   │   │   ├── CS312Efreq001.out
│   │   │   ├── CS312Em06tzvp001.out
│   │   │   ├── CS312Em06tzvp001.sdf
│   │   │   ├── CS31Efreq001.out
│   │   │   ├── CS31Em06tzvp001.out
│   │   │   ├── CS31Em06tzvp001.sdf
│   │   │   ├── CS9Efreq001.out
│   │   │   ├── CS9Em06tzvp001.out
│   │   │   ├── CS9Em06tzvp001.sdf
│   │   │   ├── CS_AmA1Bfreq001.out
│   │   │   ├── CS_AmA1Bm06tzvp001.out
│   │   │   ├── CS_AmA1Bm06tzvp001.sdf
│   │   │   ├── CS_AmA214Bfreq001.out
│   │   │   ├── CS_AmA214Bm06tzvp001.out
│   │   │   ├── CS_AmA214Bm06tzvp001.sdf
│   │   │   ├── CS_AmA353Bfreq001.out
│   │   │   ├── CS_AmA353Bm06tzvp001.out
│   │   │   ├── CS_AmA353Bm06tzvp001.sdf
│   │   │   ├── CS_AmA99Bfreq001.out
│   │   │   ├── CS_AmA99Bm06tzvp001.out
│   │   │   ├── CS_AmA99Bm06tzvp001.sdf
│   │   │   ├── CS_AmT155Bfreq001.out
│   │   │   ├── CS_AmT155Bm06tzvp001.out
│   │   │   ├── CS_AmT155Bm06tzvp001.sdf
│   │   │   ├── CS_AmT1Bfreq001.out
│   │   │   ├── CS_AmT1Bm06tzvp001.out
│   │   │   ├── CS_AmT1Bm06tzvp001.sdf
│   │   │   ├── CS_AmT68Bfreq001.out
│   │   │   ├── CS_AmT68Bm06tzvp001.out
│   │   │   ├── CS_AmT68Bm06tzvp001.sdf
│   │   │   ├── CS_AmT882Bfreq001.out
│   │   │   ├── CS_AmT882Bm06tzvp001.out
│   │   │   └── CS_AmT882Bm06tzvp001.sdf
│   │   ├── LowestR_AR_AmA21Efreq001.out
│   │   ├── LowestR_AR_AmA21Em06tzvp001.out
│   │   ├── LowestR_AR_AmA21Em06tzvp001.sdf
│   │   ├── LowestS_CS_AmA1Bfreq001.out
│   │   ├── LowestS_CS_AmA1Bm06tzvp001.out
│   │   └── LowestS_CS_AmA1Bm06tzvp001.sdf
│   └── Solvent_geometries
│       ├── AR
│       │   ├── AR_AmA1Tfreq001.out
│       │   ├── AR_AmA1Tm06tzvp001.out
│       │   ├── AR_AmA1Tm06tzvp001.sdf
│       │   ├── AR_AmA21Tfreq001.out
│       │   ├── AR_AmA21Tm06tzvp001.out
│       │   ├── AR_AmA21Tm06tzvp001.sdf
│       │   ├── AR_AmA22Dfreq001.out
│       │   ├── AR_AmA22Dm06tzvp001.out
│       │   ├── AR_AmA22Dm06tzvp001.sdf
│       │   ├── AR_AmT27Tfreq001.out
│       │   ├── AR_AmT27Tm06tzvp001.out
```

```
│       │   ├── AR_AmT27Tm06tzvp001.sdf
│       │   ├── AR_AmT463Tfreq001.out
│       │   ├── AR_AmT463Tm06tzvp001.out
│       │   └── AR_AmT463Tm06tzvp001.sdf
│       ├── AS
│       │   ├── AS14Efreq001.out
│       │   ├── AS14Em06tzvp001.out
│       │   ├── AS14Em06tzvp001.sdf
│       │   ├── AS_AmA1Efreq001.out
│       │   ├── AS_AmA1Em06tzvp001.out
│       │   ├── AS_AmA1Em06tzvp001.sdf
│       │   ├── AS_AmA2Efreq001.out
│       │   ├── AS_AmA2Em06tzvp001.out
│       │   ├── AS_AmA2Em06tzvp001.sdf
│       │   ├── AS_AmT1Efreq001.out
│       │   ├── AS_AmT1Em06tzvp001.out
│       │   └── AS_AmT1Em06tzvp001.sdf
│       ├── CR
│       │   ├── CR_AmA58ACfreq001.out
│       │   ├── CR_AmA58ACm06tzvp001.out
│       │   ├── CR_AmA58ACm06tzvp001.sdf
│       │   ├── CR_AmA7ACfreq001.out
│       │   ├── CR_AmA7ACm06tzvp001.out
│       │   ├── CR_AmA7ACm06tzvp001.sdf
│       │   ├── CR_AmT1ACfreq001.out
│       │   ├── CR_AmT1ACm06tzvp001.out
│       │   └── CR_AmT1ACm06tzvp001.sdf
│       ├── CS
│       │   ├── CS_AmA1Ffreq001.out
│       │   ├── CS_AmA1Fm06tzvp001.out
│       │   ├── CS_AmA1Fm06tzvp001.sdf
│       │   ├── CS_AmA353Ffreq001.out
│       │   ├── CS_AmA353Fm06tzvp001.out
│       │   ├── CS_AmA353Fm06tzvp001.sdf
│       │   ├── CS_AmT68Ffreq001.out
│       │   ├── CS_AmT68Fm06tzvp001.out
│       │   ├── CS_AmT68Fm06tzvp001.sdf
│       │   ├── CS_AmT882Ffreq001.out
│       │   ├── CS_AmT882Fm06tzvp001.out
│       │   └── CS_AmT882Fm06tzvp001.sdf
│       ├── LowestR_CR_AmA7ACfreq001.out
│       ├── LowestR_CR_AmA7ACm06tzvp001.out
│       ├── LowestR_CR_AmA7ACm06tzvp001.sdf
│       ├── LowestS_AS_AmT1Efreq001.out
│       ├── LowestS_AS_AmT1Em06tzvp001.out
│       └── LowestS_AS_AmT1Em06tzvp001.sdf
├── Fig9
│   ├── AS_AmA1C.sdf
│   ├── AS_AmA1Cfreq001.out
│   ├── AS_AmA1Cm06tzvp001.out
│   ├── AS_AmB1C.sdf
│   ├── AS_AmB1Cfreq001.out
│   ├── AS_AmB1Cm06tzvp001.out
│   ├── AS_AmB2C.sdf
│   ├── AS_AmB2Cfreq001.out
│   ├── AS_AmB2Cm06tzvp001.out
│   ├── AS_AmT1C.sdf
│   ├── AS_AmT1Cfreq001.out
│   ├── AS_AmT1Cm06tzvp001.out
```

```
│   ├── AS_AmU1C.sdf
│   ├── AS_AmU1Cfreq001.out
│   ├── AS_AmU1Cm06tzvp001.out
│   ├── CS_AmA1C.sdf
│   ├── CS_AmA1Cfreq001.out
│   ├── CS_AmA1Cm06tzvp001.out
│   ├── CS_AmB1C.sdf
│   ├── CS_AmB1Cfreq001.out
│   ├── CS_AmB1Cm06tzvp001.out
│   ├── CS_AmB2C.sdf
│   ├── CS_AmB2Cfreq001.out
│   ├── CS_AmB2Cm06tzvp001.out
│   ├── CS_AmT1C.sdf
│   ├── CS_AmT1Cfreq001.out
│   ├── CS_AmT1Cm06tzvp001.out
│   ├── CS_AmU1C.sdf
│   ├── CS_AmU1Cfreq001.out
│   └── CS_AmU1Cm06tzvp001.out
├── Fig10
│   ├── Int1Bfreq001.out
│   ├── Int1Bm06tzvp001.out
│   ├── Int1Bm06tzvp001.sdf
│   ├── Int2Bfreq001.out
│   ├── Int2Bm06tzvp001.out
│   ├── Int2Bm06tzvp001.sdf
│   ├── Int3Bfreq001.out
│   ├── Int3Bm06tzvp001.out
│   ├── Int3Bm06tzvp001.sdf
│   ├── Int4Bfreq001.out
│   ├── Int4Bm06tzvp001.out
│   ├── Int4Bm06tzvp001.sdf
│   ├── Int5Bfreq001.out
│   ├── Int5Bm06tzvp001.out
│   ├── Int5Bm06tzvp001.sdf
│   ├── Int6Bfreq001.out
│   ├── Int6Bm06tzvp001.out
│   ├── Int6Bm06tzvp001.sdf
│   ├── Int7Bfreq001.out
│   ├── Int7Bm06tzvp001.out
│   ├── Int7Bm06tzvp001.sdf
│   ├── Int8Bfreq001.out
│   ├── Int8Bm06tzvp001.out
│   ├── Int8Bm06tzvp001.sdf
│   ├── Int9Bfreq001.out
│   ├── Int9Bm06tzvp001.out
│   ├── Int9Bm06tzvp001.sdf
│   ├── IntP10Bfreq001.out
│   ├── IntP10Bm06tzvp001.out
│   ├── IntP10Bm06tzvp001.sdf
│   ├── IntP11Bfreq001.out
│   ├── IntP11Bm06tzvp001.out
│   ├── IntP11Bm06tzvp001.sdf
│   ├── IntP12Bfreq001.out
│   ├── IntP12Bm06tzvp001.out
│   ├── IntP12Bm06tzvp001.sdf
│   ├── IntP13Bfreq001.out
│   ├── IntP13Bm06tzvp001.out
│   └── IntP13Bm06tzvp001.sdf
├── Fig11_phosphinamideQ_full_study
```

```
|   ├── Gas_geometries
|   |   ├── AR
|   |   |   ├── AR10Bfreq001.out
|   |   |   ├── AR10Bm06tzvp001.out
|   |   |   ├── AR10Bm06tzvp001.sdf
|   |   |   ├── AR2Ffreq001.out
|   |   |   ├── AR2Fm06tzvp001.out
|   |   |   ├── AR2Fm06tzvp001.sdf
|   |   |   ├── AR384Ffreq001.out
|   |   |   ├── AR384Fm06tzvp001.out
|   |   |   ├── AR384Fm06tzvp001.sdf
|   |   |   ├── AR3Bfreq001.out
|   |   |   ├── AR3Bm06tzvp001.out
|   |   |   ├── AR3Bm06tzvp001.sdf
|   |   |   ├── AR98Ffreq001.out
|   |   |   ├── AR98Fm06tzvp001.out
|   |   |   ├── AR98Fm06tzvp001.sdf
|   |   |   ├── AR99Bfreq001.out
|   |   |   ├── AR99Bm06tzvp001.out
|   |   |   ├── AR99Bm06tzvp001.sdf
|   |   |   ├── AR9Ffreq001.out
|   |   |   ├── AR9Fm06tzvp001.out
|   |   |   ├── AR9Fm06tzvp001.sdf
|   |   |   ├── AR_AmA124Bfreq001.out
|   |   |   ├── AR_AmA124Bm06tzvp001.out
|   |   |   ├── AR_AmA124Bm06tzvp001.sdf
|   |   |   ├── AR_AmA126Bfreq001.out
|   |   |   ├── AR_AmA126Bm06tzvp001.out
|   |   |   ├── AR_AmA126Bm06tzvp001.sdf
|   |   |   ├── AR_AmA1Cfreq001.out
|   |   |   ├── AR_AmA1Cm06tzvp001.out
|   |   |   ├── AR_AmA1Cm06tzvp001.sdf
|   |   |   ├── AR_AmA72Bfreq001.out
|   |   |   ├── AR_AmA72Bm06tzvp001.out
|   |   |   ├── AR_AmA72Bm06tzvp001.sdf
|   |   |   ├── AR_AmA86Bfreq001.out
|   |   |   ├── AR_AmA86Bm06tzvp001.out
|   |   |   ├── AR_AmA86Bm06tzvp001.sdf
|   |   |   ├── AR_AmAb14Cfreq001.out
|   |   |   ├── AR_AmAb14Cm06tzvp001.out
|   |   |   ├── AR_AmAb14Cm06tzvp001.sdf
|   |   |   ├── AR_AmAb1Cfreq001.out
|   |   |   ├── AR_AmAb1Cm06tzvp001.out
|   |   |   ├── AR_AmAb1Cm06tzvp001.sdf
|   |   |   ├── AR_AmAb265Cfreq001.out
|   |   |   ├── AR_AmAb265Cm06tzvp001.out
|   |   |   ├── AR_AmAb265Cm06tzvp001.sdf
|   |   |   ├── AR_AmAb492Cfreq001.out
|   |   |   ├── AR_AmAb492Cm06tzvp001.out
|   |   |   ├── AR_AmAb492Cm06tzvp001.sdf
|   |   |   ├── AR_AmAd101Bfreq001.out
|   |   |   ├── AR_AmAd101Bm06tzvp001.out
|   |   |   ├── AR_AmAd101Bm06tzvp001.sdf
|   |   |   ├── AR_AmAd1Bfreq001.out
|   |   |   ├── AR_AmAd1Bm06tzvp001.out
|   |   |   ├── AR_AmAd1Bm06tzvp001.sdf
|   |   |   ├── AR_AmAd5Bfreq001.out
|   |   |   ├── AR_AmAd5Bm06tzvp001.out
|   |   |   ├── AR_AmAd5Bm06tzvp001.sdf
```

```
│   │   │   ├── AR_AmT17Cfreq001.out
│   │   │   ├── AR_AmT17Cm06tzvp001.out
│   │   │   ├── AR_AmT17Cm06tzvp001.sdf
│   │   │   ├── AR_AmT1Cfreq001.out
│   │   │   ├── AR_AmT1Cm06tzvp001.out
│   │   │   ├── AR_AmT1Cm06tzvp001.sdf
│   │   │   ├── AR_AmT2483Cfreq001.out
│   │   │   ├── AR_AmT2483Cm06tzvp001.out
│   │   │   ├── AR_AmT2483Cm06tzvp001.sdf
│   │   │   ├── AR_AmT304Cfreq001.out
│   │   │   ├── AR_AmT304Cm06tzvp001.out
│   │   │   ├── AR_AmT304Cm06tzvp001.sdf
│   │   │   ├── AR_AmT46Cfreq001.out
│   │   │   ├── AR_AmT46Cm06tzvp001.out
│   │   │   ├── AR_AmT46Cm06tzvp001.sdf
│   │   │   ├── AR_AmT4Cfreq001.out
│   │   │   ├── AR_AmT4Cm06tzvp001.out
│   │   │   ├── AR_AmT4Cm06tzvp001.sdf
│   │   │   ├── AR_AmT50Cfreq001.out
│   │   │   ├── AR_AmT50Cm06tzvp001.out
│   │   │   ├── AR_AmT50Cm06tzvp001.sdf
│   │   │   ├── AR_AmTa1Cfreq001.out
│   │   │   ├── AR_AmTa1Cm06tzvp001.out
│   │   │   ├── AR_AmTa1Cm06tzvp001.sdf
│   │   │   ├── AR_AmTd1003Bfreq001.out
│   │   │   ├── AR_AmTd1003Bm06tzvp001.out
│   │   │   ├── AR_AmTd1003Bm06tzvp001.sdf
│   │   │   ├── AR_AmTd1Bfreq001.out
│   │   │   ├── AR_AmTd1Bm06tzvp001.out
│   │   │   ├── AR_AmTd1Bm06tzvp001.sdf
│   │   │   ├── AR_AmTd37Bfreq001.out
│   │   │   ├── AR_AmTd37Bm06tzvp001.out
│   │   │   ├── AR_AmTd37Bm06tzvp001.sdf
│   │   │   ├── AR_AmTd3Bfreq001.out
│   │   │   ├── AR_AmTd3Bm06tzvp001.out
│   │   │   ├── AR_AmTd3Bm06tzvp001.sdf
│   │   │   ├── ARb737Ffreq001.out
│   │   │   ├── ARb737Fm06tzvp001.out
│   │   │   └── ARb737Fm06tzvp001.sdf
│   │   ├── AS
│   │   │   ├── AS1Dfreq001.out
│   │   │   ├── AS1Dm06tzvp001.out
│   │   │   ├── AS1Dm06tzvp001.sdf
│   │   │   ├── AS307Dfreq001.out
│   │   │   ├── AS307Dm06tzvp001.out
│   │   │   ├── AS307Dm06tzvp001.sdf
│   │   │   ├── AS379Dfreq001.out
│   │   │   ├── AS379Dm06tzvp001.out
│   │   │   ├── AS379Dm06tzvp001.sdf
│   │   │   ├── AS406Dfreq001.out
│   │   │   ├── AS406Dm06tzvp001.out
│   │   │   ├── AS406Dm06tzvp001.sdf
│   │   │   ├── AS407Dfreq001.out
│   │   │   ├── AS407Dm06tzvp001.out
│   │   │   ├── AS407Dm06tzvp001.sdf
│   │   │   ├── AS448Dfreq001.out
│   │   │   ├── AS448Dm06tzvp001.out
│   │   │   ├── AS448Dm06tzvp001.sdf
│   │   │   ├── AS449Dfreq001.out
```

```
|   |   |   ├── AS449Dm06tzvp001.out
|   |   |   ├── AS449Dm06tzvp001.sdf
|   |   |   ├── AS5Dfreq001.out
|   |   |   ├── AS5Dm06tzvp001.out
|   |   |   ├── AS5Dm06tzvp001.sdf
|   |   |   ├── AS6Dfreq001.out
|   |   |   ├── AS6Dm06tzvp001.out
|   |   |   ├── AS6Dm06tzvp001.sdf
|   |   |   ├── AS787Dfreq001.out
|   |   |   ├── AS787Dm06tzvp001.out
|   |   |   ├── AS787Dm06tzvp001.sdf
|   |   |   ├── AS788Dfreq001.out
|   |   |   ├── AS788Dm06tzvp001.out
|   |   |   ├── AS788Dm06tzvp001.sdf
|   |   |   ├── AS_AmA117Cfreq001.out
|   |   |   ├── AS_AmA117Cm06tzvp001.out
|   |   |   ├── AS_AmA117Cm06tzvp001.sdf
|   |   |   ├── AS_AmA1Cfreq001.out
|   |   |   ├── AS_AmA1Cm06tzvp001.out
|   |   |   ├── AS_AmA1Cm06tzvp001.sdf
|   |   |   ├── AS_AmA28Cfreq001.out
|   |   |   ├── AS_AmA28Cm06tzvp001.out
|   |   |   ├── AS_AmA28Cm06tzvp001.sdf
|   |   |   ├── AS_AmA987Cfreq001.out
|   |   |   ├── AS_AmA987Cm06tzvp001.out
|   |   |   ├── AS_AmA987Cm06tzvp001.sdf
|   |   |   ├── AS_AmAb19Cfreq001.out
|   |   |   ├── AS_AmAb19Cm06tzvp001.out
|   |   |   ├── AS_AmAb19Cm06tzvp001.sdf
|   |   |   ├── AS_AmAb1Cfreq001.out
|   |   |   ├── AS_AmAb1Cm06tzvp001.out
|   |   |   ├── AS_AmAb1Cm06tzvp001.sdf
|   |   |   ├── AS_AmAb55Cfreq001.out
|   |   |   ├── AS_AmAb55Cm06tzvp001.out
|   |   |   ├── AS_AmAb55Cm06tzvp001.sdf
|   |   |   ├── AS_AmAd1Bfreq001.out
|   |   |   ├── AS_AmAd1Bm06tzvp001.out
|   |   |   ├── AS_AmAd1Bm06tzvp001.sdf
|   |   |   ├── AS_AmAd36Bfreq001.out
|   |   |   ├── AS_AmAd36Bm06tzvp001.out
|   |   |   ├── AS_AmAd36Bm06tzvp001.sdf
|   |   |   ├── AS_AmT125Efreq001.out
|   |   |   ├── AS_AmT125Em06tzvp001.out
|   |   |   ├── AS_AmT125Em06tzvp001.sdf
|   |   |   ├── AS_AmT128Efreq001.out
|   |   |   ├── AS_AmT128Em06tzvp001.out
|   |   |   ├── AS_AmT128Em06tzvp001.sdf
|   |   |   ├── AS_AmT16Efreq001.out
|   |   |   ├── AS_AmT16Em06tzvp001.out
|   |   |   ├── AS_AmT16Em06tzvp001.sdf
|   |   |   ├── AS_AmT1Cfreq001.out
|   |   |   ├── AS_AmT1Cm06tzvp001.out
|   |   |   ├── AS_AmT1Cm06tzvp001.sdf
|   |   |   ├── AS_AmT282Efreq001.out
|   |   |   ├── AS_AmT282Em06tzvp001.out
|   |   |   ├── AS_AmT282Em06tzvp001.sdf
|   |   |   ├── AS_AmTb11Cfreq001.out
|   |   |   ├── AS_AmTb11Cm06tzvp001.out
|   |   |   ├── AS_AmTb11Cm06tzvp001.sdf
```

```
│   │   │   ├── AS_AmTb1Cfreq001.out
│   │   │   ├── AS_AmTb1Cm06tzvp001.out
│   │   │   ├── AS_AmTb1Cm06tzvp001.sdf
│   │   │   ├── AS_AmTb273Cfreq001.out
│   │   │   ├── AS_AmTb273Cm06tzvp001.out
│   │   │   ├── AS_AmTb273Cm06tzvp001.sdf
│   │   │   ├── AS_AmTb2Cfreq001.out
│   │   │   ├── AS_AmTb2Cm06tzvp001.out
│   │   │   ├── AS_AmTb2Cm06tzvp001.sdf
│   │   │   ├── AS_AmTb504Cfreq001.out
│   │   │   ├── AS_AmTb504Cm06tzvp001.out
│   │   │   ├── AS_AmTb504Cm06tzvp001.sdf
│   │   │   ├── AS_AmTd1Bfreq001.out
│   │   │   ├── AS_AmTd1Bm06tzvp001.out
│   │   │   └── AS_AmTd1Bm06tzvp001.sdf
│   │   ├── CR
│   │   │   ├── CR2Ffreq001.out
│   │   │   ├── CR2Fm06tzvp001.out
│   │   │   ├── CR2Fm06tzvp001.sdf
│   │   │   ├── CR507Ffreq001.out
│   │   │   ├── CR507Fm06tzvp001.out
│   │   │   ├── CR507Fm06tzvp001.sdf
│   │   │   ├── CR508Bfreq001.out
│   │   │   ├── CR508Bm06tzvp001.out
│   │   │   ├── CR508Bm06tzvp001.sdf
│   │   │   ├── CR_AmA12Cfreq001.out
│   │   │   ├── CR_AmA12Cm06tzvp001.out
│   │   │   ├── CR_AmA12Cm06tzvp001.sdf
│   │   │   ├── CR_AmA195Cfreq001.out
│   │   │   ├── CR_AmA195Cm06tzvp001.out
│   │   │   ├── CR_AmA195Cm06tzvp001.sdf
│   │   │   ├── CR_AmA1Bfreq001.out
│   │   │   ├── CR_AmA1Bm06tzvp001.out
│   │   │   ├── CR_AmA1Bm06tzvp001.sdf
│   │   │   ├── CR_AmA253Cfreq001.out
│   │   │   ├── CR_AmA253Cm06tzvp001.out
│   │   │   ├── CR_AmA253Cm06tzvp001.sdf
│   │   │   ├── CR_AmA28Cfreq001.out
│   │   │   ├── CR_AmA28Cm06tzvp001.out
│   │   │   ├── CR_AmA28Cm06tzvp001.sdf
│   │   │   ├── CR_AmA7Cfreq001.out
│   │   │   ├── CR_AmA7Cm06tzvp001.out
│   │   │   ├── CR_AmA7Cm06tzvp001.sdf
│   │   │   ├── CR_AmAb1Bfreq001.out
│   │   │   ├── CR_AmAb1Bm06tzvp001.out
│   │   │   ├── CR_AmAb1Bm06tzvp001.sdf
│   │   │   ├── CR_AmAb24Bfreq001.out
│   │   │   ├── CR_AmAb24Bm06tzvp001.out
│   │   │   ├── CR_AmAb24Bm06tzvp001.sdf
│   │   │   ├── CR_AmAd1102Cfreq001.out
│   │   │   ├── CR_AmAd1102Cm06tzvp001.out
│   │   │   ├── CR_AmAd1102Cm06tzvp001.sdf
│   │   │   ├── CR_AmAd1Cfreq001.out
│   │   │   ├── CR_AmAd1Cm06tzvp001.out
│   │   │   ├── CR_AmAd1Cm06tzvp001.sdf
│   │   │   ├── CR_AmAd3Cfreq001.out
│   │   │   ├── CR_AmAd3Cm06tzvp001.out
│   │   │   ├── CR_AmAd3Cm06tzvp001.sdf
│   │   │   ├── CR_AmAd716Cfreq001.out
```

```
│   │   │   ├── CR_AmAd716Cm06tzvp001.out
│   │   │   ├── CR_AmAd716Cm06tzvp001.sdf
│   │   │   ├── CR_AmT152Cfreq001.out
│   │   │   ├── CR_AmT152Cm06tzvp001.out
│   │   │   ├── CR_AmT152Cm06tzvp001.sdf
│   │   │   ├── CR_AmT16Cfreq001.out
│   │   │   ├── CR_AmT16Cm06tzvp001.out
│   │   │   ├── CR_AmT16Cm06tzvp001.sdf
│   │   │   ├── CR_AmT1Bfreq001.out
│   │   │   ├── CR_AmT1Bm06tzvp001.out
│   │   │   ├── CR_AmT1Bm06tzvp001.sdf
│   │   │   ├── CR_AmT28Cfreq001.out
│   │   │   ├── CR_AmT28Cm06tzvp001.out
│   │   │   ├── CR_AmT28Cm06tzvp001.sdf
│   │   │   ├── CR_AmT78Cfreq001.out
│   │   │   ├── CR_AmT78Cm06tzvp001.out
│   │   │   ├── CR_AmT78Cm06tzvp001.sdf
│   │   │   ├── CR_AmTb1Bfreq001.out
│   │   │   ├── CR_AmTb1Bm06tzvp001.out
│   │   │   ├── CR_AmTb1Bm06tzvp001.sdf
│   │   │   ├── CR_AmTc137Bfreq001.out
│   │   │   ├── CR_AmTc137Bm06tzvp001.out
│   │   │   ├── CR_AmTc137Bm06tzvp001.sdf
│   │   │   ├── CR_AmTc1Bfreq001.out
│   │   │   ├── CR_AmTc1Bm06tzvp001.out
│   │   │   ├── CR_AmTc1Bm06tzvp001.sdf
│   │   │   ├── CR_AmTc539Bfreq001.out
│   │   │   ├── CR_AmTc539Bm06tzvp001.out
│   │   │   ├── CR_AmTc539Bm06tzvp001.sdf
│   │   │   ├── CR_AmTd14Cfreq001.out
│   │   │   ├── CR_AmTd14Cm06tzvp001.out
│   │   │   ├── CR_AmTd14Cm06tzvp001.sdf
│   │   │   ├── CR_AmTd1Cfreq001.out
│   │   │   ├── CR_AmTd1Cm06tzvp001.out
│   │   │   ├── CR_AmTd1Cm06tzvp001.sdf
│   │   │   ├── CR_AmTd38Cfreq001.out
│   │   │   ├── CR_AmTd38Cm06tzvp001.out
│   │   │   └── CR_AmTd38Cm06tzvp001.sdf
│   │   ├── CS
│   │   │   ├── CS10Ffreq001.out
│   │   │   ├── CS10Fm06tzvp001.out
│   │   │   ├── CS10Fm06tzvp001.sdf
│   │   │   ├── CS11Ffreq001.out
│   │   │   ├── CS11Fm06tzvp001.out
│   │   │   ├── CS11Fm06tzvp001.sdf
│   │   │   ├── CS1Ffreq001.out
│   │   │   ├── CS1Fm06tzvp001.out
│   │   │   ├── CS1Fm06tzvp001.sdf
│   │   │   ├── CS2Ffreq001.out
│   │   │   ├── CS2Fm06tzvp001.out
│   │   │   ├── CS2Fm06tzvp001.sdf
│   │   │   ├── CS301Ffreq001.out
│   │   │   ├── CS301Fm06tzvp001.out
│   │   │   ├── CS301Fm06tzvp001.sdf
│   │   │   ├── CS302Ffreq001.out
│   │   │   ├── CS302Fm06tzvp001.out
│   │   │   ├── CS302Fm06tzvp001.sdf
│   │   │   ├── CS414Ffreq001.out
│   │   │   ├── CS414Fm06tzvp001.out
```

```
│   │   │   ├── CS414Fm06tzvp001.sdf
│   │   │   ├── CS415Ffreq001.out
│   │   │   ├── CS415Fm06tzvp001.out
│   │   │   ├── CS415Fm06tzvp001.sdf
│   │   │   ├── CS51Ffreq001.out
│   │   │   ├── CS51Fm06tzvp001.out
│   │   │   ├── CS51Fm06tzvp001.sdf
│   │   │   ├── CS52Ffreq001.out
│   │   │   ├── CS52Fm06tzvp001.out
│   │   │   ├── CS52Fm06tzvp001.sdf
│   │   │   ├── CS_AmA1Bfreq001.out
│   │   │   ├── CS_AmA1Bm06tzvp001.out
│   │   │   ├── CS_AmA1Bm06tzvp001.sdf
│   │   │   ├── CS_AmA23Bfreq001.out
│   │   │   ├── CS_AmA23Bm06tzvp001.out
│   │   │   ├── CS_AmA23Bm06tzvp001.sdf
│   │   │   ├── CS_AmA52Bfreq001.out
│   │   │   ├── CS_AmA52Bm06tzvp001.out
│   │   │   ├── CS_AmA52Bm06tzvp001.sdf
│   │   │   ├── CS_AmA91Bfreq001.out
│   │   │   ├── CS_AmA91Bm06tzvp001.out
│   │   │   ├── CS_AmA91Bm06tzvp001.sdf
│   │   │   ├── CS_AmAb169Bfreq001.out
│   │   │   ├── CS_AmAb169Bm06tzvp001.out
│   │   │   ├── CS_AmAb169Bm06tzvp001.sdf
│   │   │   ├── CS_AmAb1Bfreq001.out
│   │   │   ├── CS_AmAb1Bm06tzvp001.out
│   │   │   ├── CS_AmAb1Bm06tzvp001.sdf
│   │   │   ├── CS_AmAd12Bfreq001.out
│   │   │   ├── CS_AmAd12Bm06tzvp001.out
│   │   │   ├── CS_AmAd12Bm06tzvp001.sdf
│   │   │   ├── CS_AmAd142Bfreq001.out
│   │   │   ├── CS_AmAd142Bm06tzvp001.out
│   │   │   ├── CS_AmAd142Bm06tzvp001.sdf
│   │   │   ├── CS_AmAd1Bfreq001.out
│   │   │   ├── CS_AmAd1Bm06tzvp001.out
│   │   │   ├── CS_AmAd1Bm06tzvp001.sdf
│   │   │   ├── CS_AmT138Cfreq001.out
│   │   │   ├── CS_AmT138Cm06tzvp001.out
│   │   │   ├── CS_AmT138Cm06tzvp001.sdf
│   │   │   ├── CS_AmT144Cfreq001.out
│   │   │   ├── CS_AmT144Cm06tzvp001.out
│   │   │   ├── CS_AmT144Cm06tzvp001.sdf
│   │   │   ├── CS_AmT145Bfreq001.out
│   │   │   ├── CS_AmT145Bm06tzvp001.out
│   │   │   ├── CS_AmT145Bm06tzvp001.sdf
│   │   │   ├── CS_AmT146Bfreq001.out
│   │   │   ├── CS_AmT146Bm06tzvp001.out
│   │   │   ├── CS_AmT146Bm06tzvp001.sdf
│   │   │   ├── CS_AmT18Cfreq001.out
│   │   │   ├── CS_AmT18Cm06tzvp001.out
│   │   │   ├── CS_AmT18Cm06tzvp001.sdf
│   │   │   ├── CS_AmT1Bfreq001.out
│   │   │   ├── CS_AmT1Bm06tzvp001.out
│   │   │   ├── CS_AmT1Bm06tzvp001.sdf
│   │   │   ├── CS_AmT240Cfreq001.out
│   │   │   ├── CS_AmT240Cm06tzvp001.out
│   │   │   ├── CS_AmT240Cm06tzvp001.sdf
│   │   │   ├── CS_AmT96Cfreq001.out
```

```
│   │   │   ├── CS_AmT96Cm06tzvp001.out
│   │   │   ├── CS_AmT96Cm06tzvp001.sdf
│   │   │   ├── CS_AmTb1Bfreq001.out
│   │   │   ├── CS_AmTb1Bm06tzvp001.out
│   │   │   ├── CS_AmTb1Bm06tzvp001.sdf
│   │   │   ├── CS_AmTb22Bfreq001.out
│   │   │   ├── CS_AmTb22Bm06tzvp001.out
│   │   │   ├── CS_AmTb22Bm06tzvp001.sdf
│   │   │   ├── CS_AmTb287Bfreq001.out
│   │   │   ├── CS_AmTb287Bm06tzvp001.out
│   │   │   ├── CS_AmTb287Bm06tzvp001.sdf
│   │   │   ├── CS_AmTb37Bfreq001.out
│   │   │   ├── CS_AmTb37Bm06tzvp001.out
│   │   │   ├── CS_AmTb37Bm06tzvp001.sdf
│   │   │   ├── CS_AmTb42Bfreq001.out
│   │   │   ├── CS_AmTb42Bm06tzvp001.out
│   │   │   ├── CS_AmTb42Bm06tzvp001.sdf
│   │   │   ├── CS_AmTd19Bfreq001.out
│   │   │   ├── CS_AmTd19Bm06tzvp001.out
│   │   │   ├── CS_AmTd19Bm06tzvp001.sdf
│   │   │   ├── CS_AmTd1Bfreq001.out
│   │   │   ├── CS_AmTd1Bm06tzvp001.out
│   │   │   ├── CS_AmTd1Bm06tzvp001.sdf
│   │   │   ├── CS_AmTd76Bfreq001.out
│   │   │   ├── CS_AmTd76Bm06tzvp001.out
│   │   │   └── CS_AmTd76Bm06tzvp001.sdf
│   │   ├── LowestR_AR_AmAd1Bfreq001.out
│   │   ├── LowestR_AR_AmAd1Bm06tzvp001.out
│   │   ├── LowestR_AR_AmAd1Bm06tzvp001.sdf
│   │   ├── LowestS_CS_AmT145Bfreq001.out
│   │   ├── LowestS_CS_AmT145Bm06tzvp001.out
│   │   └── LowestS_CS_AmT145Bm06tzvp001.sdf
│   └── Solvent_geometries
│       ├── AR
│       │   ├── AR_AmAd101Jfreq001.out
│       │   ├── AR_AmAd101Jm06tzvp001.out
│       │   ├── AR_AmAd101Jm06tzvp001.sdf
│       │   ├── AR_AmAd1Jfreq001.out
│       │   ├── AR_AmAd1Jm06tzvp001.out
│       │   ├── AR_AmAd1Jm06tzvp001.sdf
│       │   ├── AR_AmAd5Jfreq001.out
│       │   ├── AR_AmAd5Jm06tzvp001.out
│       │   ├── AR_AmAd5Jm06tzvp001.sdf
│       │   ├── AR_AmT1Jfreq001.out
│       │   ├── AR_AmT1Jm06tzvp001.out
│       │   ├── AR_AmT1Jm06tzvp001.sdf
│       │   ├── AR_AmTd3Jfreq001.out
│       │   ├── AR_AmTd3Jm06tzvp001.out
│       │   └── AR_AmTd3Jm06tzvp001.sdf
│       ├── AS
│       │   ├── AS_AmAb55Ffreq001.out
│       │   ├── AS_AmAb55Fm06tzvp001.out
│       │   ├── AS_AmAb55Fm06tzvp001.sdf
│       │   ├── AS_AmTb1Ffreq001.out
│       │   ├── AS_AmTb1Fm06tzvp001.out
│       │   └── AS_AmTb1Fm06tzvp001.sdf
│       ├── CR
│       │   ├── CR_AmTb1Cfreq001.out
│       │   ├── CR_AmTb1Cm06tzvp001.out
```

```
│       │   └── CR_AmTb1Cm06tzvp001.sdf
│       ├── CS
│       │   ├── CS_AmAb1Dfreq001.out
│       │   ├── CS_AmAb1Dm06tzvp001.out
│       │   ├── CS_AmAb1Dm06tzvp001.sdf
│       │   ├── CS_AmAd142Dfreq001.out
│       │   ├── CS_AmAd142Dm06tzvp001.out
│       │   ├── CS_AmAd142Dm06tzvp001.sdf
│       │   ├── CS_AmT145Dfreq001.out
│       │   ├── CS_AmT145Dm06tzvp001.out
│       │   ├── CS_AmT145Dm06tzvp001.sdf
│       │   ├── CS_AmTd76Dfreq001.out
│       │   ├── CS_AmTd76Dm06tzvp001.out
│       │   └── CS_AmTd76Dm06tzvp001.sdf
│       ├── LowestR_AR_AmAd1Jfreq001.out
│       ├── LowestR_AR_AmAd1Jm06tzvp001.out
│       ├── LowestR_AR_AmAd1Jm06tzvp001.sdf
│       ├── LowestS_CS_AmT145Dfreq001.out
│       ├── LowestS_CS_AmT145Dm06tzvp001.out
│       └── LowestS_CS_AmT145Dm06tzvp001.sdf
├── FigS2_phosphinamideQD_full_study
│   ├── Gas_geometries
│   │   ├── AR
│   │   │   ├── AR10Efreq001.out
│   │   │   ├── AR10Em06tzvp001.out
│   │   │   ├── AR10Em06tzvp001.sdf
│   │   │   ├── AR1Efreq001.out
│   │   │   ├── AR1Em06tzvp001.out
│   │   │   ├── AR1Em06tzvp001.sdf
│   │   │   ├── AR2Efreq001.out
│   │   │   ├── AR2Em06tzvp001.out
│   │   │   ├── AR2Em06tzvp001.sdf
│   │   │   ├── AR44Efreq001.out
│   │   │   ├── AR44Em06tzvp001.out
│   │   │   ├── AR44Em06tzvp001.sdf
│   │   │   ├── AR45Efreq001.out
│   │   │   ├── AR45Em06tzvp001.out
│   │   │   ├── AR45Em06tzvp001.sdf
│   │   │   ├── AR58Efreq001.out
│   │   │   ├── AR58Em06tzvp001.out
│   │   │   ├── AR58Em06tzvp001.sdf
│   │   │   ├── AR59Efreq001.out
│   │   │   ├── AR59Em06tzvp001.out
│   │   │   ├── AR59Em06tzvp001.sdf
│   │   │   ├── AR9Efreq001.out
│   │   │   ├── AR9Em06tzvp001.out
│   │   │   ├── AR9Em06tzvp001.sdf
│   │   │   ├── AR_AmA1Bfreq001.out
│   │   │   ├── AR_AmA1Bm06tzvp001.out
│   │   │   ├── AR_AmA1Bm06tzvp001.sdf
│   │   │   ├── AR_AmA3Bfreq001.out
│   │   │   ├── AR_AmA3Bm06tzvp001.out
│   │   │   ├── AR_AmA3Bm06tzvp001.sdf
│   │   │   ├── AR_AmA45Bfreq001.out
│   │   │   ├── AR_AmA45Bm06tzvp001.out
│   │   │   ├── AR_AmA45Bm06tzvp001.sdf
│   │   │   ├── AR_AmA48Bfreq001.out
│   │   │   ├── AR_AmA48Bm06tzvp001.out
│   │   │   ├── AR_AmA48Bm06tzvp001.sdf
```

```
│   │   │   ├── AR_AmA81Bfreq001.out
│   │   │   ├── AR_AmA81Bm06tzvp001.out
│   │   │   ├── AR_AmA81Bm06tzvp001.sdf
│   │   │   ├── AR_AmA8Bfreq001.out
│   │   │   ├── AR_AmA8Bm06tzvp001.out
│   │   │   ├── AR_AmA8Bm06tzvp001.sdf
│   │   │   ├── AR_AmAb139Bfreq001.out
│   │   │   ├── AR_AmAb139Bm06tzvp001.out
│   │   │   ├── AR_AmAb139Bm06tzvp001.sdf
│   │   │   ├── AR_AmAb1Bfreq001.out
│   │   │   ├── AR_AmAb1Bm06tzvp001.out
│   │   │   ├── AR_AmAb1Bm06tzvp001.sdf
│   │   │   ├── AR_AmAd1Dfreq001.out
│   │   │   ├── AR_AmAd1Dm06tzvp001.out
│   │   │   ├── AR_AmAd1Dm06tzvp001.sdf
│   │   │   ├── AR_AmAd3820Dfreq001.out
│   │   │   ├── AR_AmAd3820Dm06tzvp001.out
│   │   │   ├── AR_AmAd3820Dm06tzvp001.sdf
│   │   │   ├── AR_AmAd4259Dfreq001.out
│   │   │   ├── AR_AmAd4259Dm06tzvp001.out
│   │   │   ├── AR_AmAd4259Dm06tzvp001.sdf
│   │   │   ├── AR_AmT117Cfreq001.out
│   │   │   ├── AR_AmT117Cm06tzvp001.out
│   │   │   ├── AR_AmT117Cm06tzvp001.sdf
│   │   │   ├── AR_AmT14Cfreq001.out
│   │   │   ├── AR_AmT14Cm06tzvp001.out
│   │   │   ├── AR_AmT14Cm06tzvp001.sdf
│   │   │   ├── AR_AmT1Dfreq001.out
│   │   │   ├── AR_AmT1Dm06tzvp001.out
│   │   │   ├── AR_AmT1Dm06tzvp001.sdf
│   │   │   ├── AR_AmT33Cfreq001.out
│   │   │   ├── AR_AmT33Cm06tzvp001.out
│   │   │   ├── AR_AmT33Cm06tzvp001.sdf
│   │   │   ├── AR_AmTb16Dfreq001.out
│   │   │   ├── AR_AmTb16Dm06tzvp001.out
│   │   │   ├── AR_AmTb16Dm06tzvp001.sdf
│   │   │   ├── AR_AmTb1Dfreq001.out
│   │   │   ├── AR_AmTb1Dm06tzvp001.out
│   │   │   ├── AR_AmTb1Dm06tzvp001.sdf
│   │   │   ├── AR_AmTb20Dfreq001.out
│   │   │   ├── AR_AmTb20Dm06tzvp001.out
│   │   │   ├── AR_AmTb20Dm06tzvp001.sdf
│   │   │   ├── AR_AmTd19Dfreq001.out
│   │   │   ├── AR_AmTd19Dm06tzvp001.out
│   │   │   ├── AR_AmTd19Dm06tzvp001.sdf
│   │   │   ├── AR_AmTd1Dfreq001.out
│   │   │   ├── AR_AmTd1Dm06tzvp001.out
│   │   │   ├── AR_AmTd1Dm06tzvp001.sdf
│   │   │   ├── AR_AmTd3Dfreq001.out
│   │   │   ├── AR_AmTd3Dm06tzvp001.out
│   │   │   ├── AR_AmTd3Dm06tzvp001.sdf
│   │   │   ├── AR_AmTd45Dfreq001.out
│   │   │   ├── AR_AmTd45Dm06tzvp001.out
│   │   │   ├── AR_AmTd45Dm06tzvp001.sdf
│   │   │   ├── AR_AmTd487Dfreq001.out
│   │   │   ├── AR_AmTd487Dm06tzvp001.out
│   │   │   ├── AR_AmTd487Dm06tzvp001.sdf
│   │   │   ├── AR_AmTe1Bfreq001.out
│   │   │   ├── AR_AmTe1Bm06tzvp001.out
```

```
│   │   │   ├── AR_AmTe1Bm06tzvp001.sdf
│   │   │   ├── AR_AmTe2Bfreq001.out
│   │   │   ├── AR_AmTe2Bm06tzvp001.out
│   │   │   ├── AR_AmTe2Bm06tzvp001.sdf
│   │   │   ├── AR_AmTe3Bfreq001.out
│   │   │   ├── AR_AmTe3Bm06tzvp001.out
│   │   │   └── AR_AmTe3Bm06tzvp001.sdf
│   │   ├── AS
│   │   │   ├── AS109Cfreq001.out
│   │   │   ├── AS109Cm06tzvp001.out
│   │   │   ├── AS109Cm06tzvp001.sdf
│   │   │   ├── AS110Cfreq001.out
│   │   │   ├── AS110Cm06tzvp001.out
│   │   │   ├── AS110Cm06tzvp001.sdf
│   │   │   ├── AS1288Cfreq001.out
│   │   │   ├── AS1288Cm06tzvp001.out
│   │   │   ├── AS1288Cm06tzvp001.sdf
│   │   │   ├── AS1289Cfreq001.out
│   │   │   ├── AS1289Cm06tzvp001.out
│   │   │   ├── AS1289Cm06tzvp001.sdf
│   │   │   ├── AS1Cfreq001.out
│   │   │   ├── AS1Cm06tzvp001.out
│   │   │   ├── AS1Cm06tzvp001.sdf
│   │   │   ├── AS2Cfreq001.out
│   │   │   ├── AS2Cm06tzvp001.out
│   │   │   ├── AS2Cm06tzvp001.sdf
│   │   │   ├── AS306Cfreq001.out
│   │   │   ├── AS306Cm06tzvp001.out
│   │   │   ├── AS306Cm06tzvp001.sdf
│   │   │   ├── AS307Cfreq001.out
│   │   │   ├── AS307Cm06tzvp001.out
│   │   │   ├── AS307Cm06tzvp001.sdf
│   │   │   ├── AS706Cfreq001.out
│   │   │   ├── AS706Cm06tzvp001.out
│   │   │   ├── AS706Cm06tzvp001.sdf
│   │   │   ├── AS707Cfreq001.out
│   │   │   ├── AS707Cm06tzvp001.out
│   │   │   ├── AS707Cm06tzvp001.sdf
│   │   │   ├── AS_AmA161Bfreq001.out
│   │   │   ├── AS_AmA161Bm06tzvp001.out
│   │   │   ├── AS_AmA161Bm06tzvp001.sdf
│   │   │   ├── AS_AmA1Cfreq001.out
│   │   │   ├── AS_AmA1Cm06tzvp001.out
│   │   │   ├── AS_AmA1Cm06tzvp001.sdf
│   │   │   ├── AS_AmA241Bfreq001.out
│   │   │   ├── AS_AmA241Bm06tzvp001.out
│   │   │   ├── AS_AmA241Bm06tzvp001.sdf
│   │   │   ├── AS_AmA31Bfreq001.out
│   │   │   ├── AS_AmA31Bm06tzvp001.out
│   │   │   ├── AS_AmA31Bm06tzvp001.sdf
│   │   │   ├── AS_AmA3Bfreq001.out
│   │   │   ├── AS_AmA3Bm06tzvp001.out
│   │   │   ├── AS_AmA3Bm06tzvp001.sdf
│   │   │   ├── AS_AmA83Bfreq001.out
│   │   │   ├── AS_AmA83Bm06tzvp001.out
│   │   │   ├── AS_AmA83Bm06tzvp001.sdf
│   │   │   ├── AS_AmAb130Cfreq001.out
│   │   │   ├── AS_AmAb130Cm06tzvp001.out
│   │   │   ├── AS_AmAb130Cm06tzvp001.sdf
```

```
│   │   │   ├── AS_AmAb150Cfreq001.out
│   │   │   ├── AS_AmAb150Cm06tzvp001.out
│   │   │   ├── AS_AmAb150Cm06tzvp001.sdf
│   │   │   ├── AS_AmAb1Cfreq001.out
│   │   │   ├── AS_AmAb1Cm06tzvp001.out
│   │   │   ├── AS_AmAb1Cm06tzvp001.sdf
│   │   │   ├── AS_AmAb22Cfreq001.out
│   │   │   ├── AS_AmAb22Cm06tzvp001.out
│   │   │   ├── AS_AmAb22Cm06tzvp001.sdf
│   │   │   ├── AS_AmAd1758Dfreq001.out
│   │   │   ├── AS_AmAd1758Dm06tzvp001.out
│   │   │   ├── AS_AmAd1758Dm06tzvp001.sdf
│   │   │   ├── AS_AmAd1Dfreq001.out
│   │   │   ├── AS_AmAd1Dm06tzvp001.out
│   │   │   ├── AS_AmAd1Dm06tzvp001.sdf
│   │   │   ├── AS_AmAd2Dfreq001.out
│   │   │   ├── AS_AmAd2Dm06tzvp001.out
│   │   │   ├── AS_AmAd2Dm06tzvp001.sdf
│   │   │   ├── AS_AmAd7077Dfreq001.out
│   │   │   ├── AS_AmAd7077Dm06tzvp001.out
│   │   │   ├── AS_AmAd7077Dm06tzvp001.sdf
│   │   │   ├── AS_AmT186Bfreq001.out
│   │   │   ├── AS_AmT186Bm06tzvp001.out
│   │   │   ├── AS_AmT186Bm06tzvp001.sdf
│   │   │   ├── AS_AmT1Bfreq001.out
│   │   │   ├── AS_AmT1Bm06tzvp001.out
│   │   │   ├── AS_AmT1Bm06tzvp001.sdf
│   │   │   ├── AS_AmT522Bfreq001.out
│   │   │   ├── AS_AmT522Bm06tzvp001.out
│   │   │   ├── AS_AmT522Bm06tzvp001.sdf
│   │   │   ├── AS_AmT5Bfreq001.out
│   │   │   ├── AS_AmT5Bm06tzvp001.out
│   │   │   ├── AS_AmT5Bm06tzvp001.sdf
│   │   │   ├── AS_AmT725Bfreq001.out
│   │   │   ├── AS_AmT725Bm06tzvp001.out
│   │   │   ├── AS_AmT725Bm06tzvp001.sdf
│   │   │   ├── AS_AmT86Bfreq001.out
│   │   │   ├── AS_AmT86Bm06tzvp001.out
│   │   │   ├── AS_AmT86Bm06tzvp001.sdf
│   │   │   ├── AS_AmTb136Bfreq001.out
│   │   │   ├── AS_AmTb136Bm06tzvp001.out
│   │   │   ├── AS_AmTb136Bm06tzvp001.sdf
│   │   │   ├── AS_AmTb13Bfreq001.out
│   │   │   ├── AS_AmTb13Bm06tzvp001.out
│   │   │   ├── AS_AmTb13Bm06tzvp001.sdf
│   │   │   ├── AS_AmTb1Bfreq001.out
│   │   │   ├── AS_AmTb1Bm06tzvp001.out
│   │   │   ├── AS_AmTb1Bm06tzvp001.sdf
│   │   │   ├── AS_AmTd1Dfreq001.out
│   │   │   ├── AS_AmTd1Dm06tzvp001.out
│   │   │   ├── AS_AmTd1Dm06tzvp001.sdf
│   │   │   ├── AS_AmTd240Dfreq001.out
│   │   │   ├── AS_AmTd240Dm06tzvp001.out
│   │   │   ├── AS_AmTd240Dm06tzvp001.sdf
│   │   │   ├── AS_AmTd35Dfreq001.out
│   │   │   ├── AS_AmTd35Dm06tzvp001.out
│   │   │   ├── AS_AmTd35Dm06tzvp001.sdf
│   │   │   ├── AS_AmTd49Dfreq001.out
│   │   │   ├── AS_AmTd49Dm06tzvp001.out
```

```
│   │   │   └── AS_AmTd49Dm06tzvp001.sdf
│   │   ├── CR
│   │   │   ├── CR10Dfreq001.out
│   │   │   ├── CR10Dm06tzvp001.out
│   │   │   ├── CR10Dm06tzvp001.sdf
│   │   │   ├── CR11Dfreq001.out
│   │   │   ├── CR11Dm06tzvp001.out
│   │   │   ├── CR11Dm06tzvp001.sdf
│   │   │   ├── CR1344Dfreq001.out
│   │   │   ├── CR1344Dm06tzvp001.out
│   │   │   ├── CR1344Dm06tzvp001.sdf
│   │   │   ├── CR1345Dfreq001.out
│   │   │   ├── CR1345Dm06tzvp001.out
│   │   │   ├── CR1345Dm06tzvp001.sdf
│   │   │   ├── CR155Dfreq001.out
│   │   │   ├── CR155Dm06tzvp001.out
│   │   │   ├── CR155Dm06tzvp001.sdf
│   │   │   ├── CR156Dfreq001.out
│   │   │   ├── CR156Dm06tzvp001.out
│   │   │   ├── CR156Dm06tzvp001.sdf
│   │   │   ├── CR19Dfreq001.out
│   │   │   ├── CR19Dm06tzvp001.out
│   │   │   ├── CR19Dm06tzvp001.sdf
│   │   │   ├── CR1Dfreq001.out
│   │   │   ├── CR1Dm06tzvp001.out
│   │   │   ├── CR1Dm06tzvp001.sdf
│   │   │   ├── CR20Dfreq001.out
│   │   │   ├── CR20Dm06tzvp001.out
│   │   │   ├── CR20Dm06tzvp001.sdf
│   │   │   ├── CR2Dfreq001.out
│   │   │   ├── CR2Dm06tzvp001.out
│   │   │   ├── CR2Dm06tzvp001.sdf
│   │   │   ├── CR692Dfreq001.out
│   │   │   ├── CR692Dm06tzvp001.out
│   │   │   ├── CR692Dm06tzvp001.sdf
│   │   │   ├── CR693Dfreq001.out
│   │   │   ├── CR693Dm06tzvp001.out
│   │   │   ├── CR693Dm06tzvp001.sdf
│   │   │   ├── CR_AmA17Bfreq001.out
│   │   │   ├── CR_AmA17Bm06tzvp001.out
│   │   │   ├── CR_AmA17Bm06tzvp001.sdf
│   │   │   ├── CR_AmA1Cfreq001.out
│   │   │   ├── CR_AmA1Cm06tzvp001.out
│   │   │   ├── CR_AmA1Cm06tzvp001.sdf
│   │   │   ├── CR_AmA70Bfreq001.out
│   │   │   ├── CR_AmA70Bm06tzvp001.out
│   │   │   ├── CR_AmA70Bm06tzvp001.sdf
│   │   │   ├── CR_AmAb1527Cfreq001.out
│   │   │   ├── CR_AmAb1527Cm06tzvp001.out
│   │   │   ├── CR_AmAb1527Cm06tzvp001.sdf
│   │   │   ├── CR_AmAb28Cfreq001.out
│   │   │   ├── CR_AmAb28Cm06tzvp001.out
│   │   │   ├── CR_AmAb28Cm06tzvp001.sdf
│   │   │   ├── CR_AmAb39Cfreq001.out
│   │   │   ├── CR_AmAb39Cm06tzvp001.out
│   │   │   ├── CR_AmAb39Cm06tzvp001.sdf
│   │   │   ├── CR_AmAd159Cfreq001.out
│   │   │   ├── CR_AmAd159Cm06tzvp001.out
│   │   │   ├── CR_AmAd159Cm06tzvp001.sdf
```

```
│   │   │   ├── CR_AmAd161Cfreq001.out
│   │   │   ├── CR_AmAd161Cm06tzvp001.out
│   │   │   ├── CR_AmAd161Cm06tzvp001.sdf
│   │   │   ├── CR_AmAd1Cfreq001.out
│   │   │   ├── CR_AmAd1Cm06tzvp001.out
│   │   │   ├── CR_AmAd1Cm06tzvp001.sdf
│   │   │   ├── CR_AmAd63Cfreq001.out
│   │   │   ├── CR_AmAd63Cm06tzvp001.out
│   │   │   ├── CR_AmAd63Cm06tzvp001.sdf
│   │   │   ├── CR_AmT132Cfreq001.out
│   │   │   ├── CR_AmT132Cm06tzvp001.out
│   │   │   ├── CR_AmT132Cm06tzvp001.sdf
│   │   │   ├── CR_AmT191Cfreq001.out
│   │   │   ├── CR_AmT191Cm06tzvp001.out
│   │   │   ├── CR_AmT191Cm06tzvp001.sdf
│   │   │   ├── CR_AmT1Cfreq001.out
│   │   │   ├── CR_AmT1Cm06tzvp001.out
│   │   │   ├── CR_AmT1Cm06tzvp001.sdf
│   │   │   ├── CR_AmT208Cfreq001.out
│   │   │   ├── CR_AmT208Cm06tzvp001.out
│   │   │   ├── CR_AmT208Cm06tzvp001.sdf
│   │   │   ├── CR_AmT257Cfreq001.out
│   │   │   ├── CR_AmT257Cm06tzvp001.out
│   │   │   ├── CR_AmT257Cm06tzvp001.sdf
│   │   │   ├── CR_AmT98Cfreq001.out
│   │   │   ├── CR_AmT98Cm06tzvp001.out
│   │   │   ├── CR_AmT98Cm06tzvp001.sdf
│   │   │   ├── CR_AmTb1Cfreq001.out
│   │   │   ├── CR_AmTb1Cm06tzvp001.out
│   │   │   ├── CR_AmTb1Cm06tzvp001.sdf
│   │   │   ├── CR_AmTb74Cfreq001.out
│   │   │   ├── CR_AmTb74Cm06tzvp001.out
│   │   │   ├── CR_AmTb74Cm06tzvp001.sdf
│   │   │   ├── CR_AmTd1Cfreq001.out
│   │   │   ├── CR_AmTd1Cm06tzvp001.out
│   │   │   ├── CR_AmTd1Cm06tzvp001.sdf
│   │   │   ├── CR_AmTd24Cfreq001.out
│   │   │   ├── CR_AmTd24Cm06tzvp001.out
│   │   │   └── CR_AmTd24Cm06tzvp001.sdf
│   │   ├── CS
│   │   │   ├── CS1484Gfreq001.out
│   │   │   ├── CS1484Gm06tzvp001.out
│   │   │   ├── CS1484Gm06tzvp001.sdf
│   │   │   ├── CS1485Gfreq001.out
│   │   │   ├── CS1485Gm06tzvp001.out
│   │   │   ├── CS1485Gm06tzvp001.sdf
│   │   │   ├── CS1486Gfreq001.out
│   │   │   ├── CS1486Gm06tzvp001.out
│   │   │   ├── CS1486Gm06tzvp001.sdf
│   │   │   ├── CS1Gfreq001.out
│   │   │   ├── CS1Gm06tzvp001.out
│   │   │   ├── CS1Gm06tzvp001.sdf
│   │   │   ├── CS2Gfreq001.out
│   │   │   ├── CS2Gm06tzvp001.out
│   │   │   ├── CS2Gm06tzvp001.sdf
│   │   │   ├── CS320Gfreq001.out
│   │   │   ├── CS320Gm06tzvp001.out
│   │   │   ├── CS320Gm06tzvp001.sdf
│   │   │   ├── CS321Gfreq001.out
```

```
│   │   │   ├── CS321Gm06tzvp001.out
│   │   │   ├── CS321Gm06tzvp001.sdf
│   │   │   ├── CS424Gfreq001.out
│   │   │   ├── CS424Gm06tzvp001.out
│   │   │   ├── CS424Gm06tzvp001.sdf
│   │   │   ├── CS425Gfreq001.out
│   │   │   ├── CS425Gm06tzvp001.out
│   │   │   ├── CS425Gm06tzvp001.sdf
│   │   │   ├── CS557Gfreq001.out
│   │   │   ├── CS557Gm06tzvp001.out
│   │   │   ├── CS557Gm06tzvp001.sdf
│   │   │   ├── CS558Gfreq001.out
│   │   │   ├── CS558Gm06tzvp001.out
│   │   │   ├── CS558Gm06tzvp001.sdf
│   │   │   ├── CS65Gfreq001.out
│   │   │   ├── CS65Gm06tzvp001.out
│   │   │   ├── CS65Gm06tzvp001.sdf
│   │   │   ├── CS66Gfreq001.out
│   │   │   ├── CS66Gm06tzvp001.out
│   │   │   ├── CS66Gm06tzvp001.sdf
│   │   │   ├── CS_AmA1Cfreq001.out
│   │   │   ├── CS_AmA1Cm06tzvp001.out
│   │   │   ├── CS_AmA1Cm06tzvp001.sdf
│   │   │   ├── CS_AmA26Cfreq001.out
│   │   │   ├── CS_AmA26Cm06tzvp001.out
│   │   │   ├── CS_AmA26Cm06tzvp001.sdf
│   │   │   ├── CS_AmA271Cfreq001.out
│   │   │   ├── CS_AmA271Cm06tzvp001.out
│   │   │   ├── CS_AmA271Cm06tzvp001.sdf
│   │   │   ├── CS_AmA800Cfreq001.out
│   │   │   ├── CS_AmA800Cm06tzvp001.out
│   │   │   ├── CS_AmA800Cm06tzvp001.sdf
│   │   │   ├── CS_AmA88Cfreq001.out
│   │   │   ├── CS_AmA88Cm06tzvp001.out
│   │   │   ├── CS_AmA88Cm06tzvp001.sdf
│   │   │   ├── CS_AmAb14Cfreq001.out
│   │   │   ├── CS_AmAb14Cm06tzvp001.out
│   │   │   ├── CS_AmAb14Cm06tzvp001.sdf
│   │   │   ├── CS_AmAb1Cfreq001.out
│   │   │   ├── CS_AmAb1Cm06tzvp001.out
│   │   │   ├── CS_AmAb1Cm06tzvp001.sdf
│   │   │   ├── CS_AmAb20Cfreq001.out
│   │   │   ├── CS_AmAb20Cm06tzvp001.out
│   │   │   ├── CS_AmAb20Cm06tzvp001.sdf
│   │   │   ├── CS_AmAb40Cfreq001.out
│   │   │   ├── CS_AmAb40Cm06tzvp001.out
│   │   │   ├── CS_AmAb40Cm06tzvp001.sdf
│   │   │   ├── CS_AmAd12Dfreq001.out
│   │   │   ├── CS_AmAd12Dm06tzvp001.out
│   │   │   ├── CS_AmAd12Dm06tzvp001.sdf
│   │   │   ├── CS_AmAd1Dfreq001.out
│   │   │   ├── CS_AmAd1Dm06tzvp001.out
│   │   │   ├── CS_AmAd1Dm06tzvp001.sdf
│   │   │   ├── CS_AmT148Cfreq001.out
│   │   │   ├── CS_AmT148Cm06tzvp001.out
│   │   │   ├── CS_AmT148Cm06tzvp001.sdf
│   │   │   ├── CS_AmT1Cfreq001.out
│   │   │   ├── CS_AmT1Cm06tzvp001.out
│   │   │   ├── CS_AmT1Cm06tzvp001.sdf
```

```
│   │   │   ├── CS_AmT2Bfreq001.out
│   │   │   ├── CS_AmT2Bm06tzvp001.out
│   │   │   ├── CS_AmT2Bm06tzvp001.sdf
│   │   │   ├── CS_AmT4Cfreq001.out
│   │   │   ├── CS_AmT4Cm06tzvp001.out
│   │   │   ├── CS_AmT4Cm06tzvp001.sdf
│   │   │   ├── CS_AmT5Bfreq001.out
│   │   │   ├── CS_AmT5Bm06tzvp001.out
│   │   │   ├── CS_AmT5Bm06tzvp001.sdf
│   │   │   ├── CS_AmTb1374Cfreq001.out
│   │   │   ├── CS_AmTb1374Cm06tzvp001.out
│   │   │   ├── CS_AmTb1374Cm06tzvp001.sdf
│   │   │   ├── CS_AmTb58Cfreq001.out
│   │   │   ├── CS_AmTb58Cm06tzvp001.out
│   │   │   ├── CS_AmTb58Cm06tzvp001.sdf
│   │   │   ├── CS_AmTb77Cfreq001.out
│   │   │   ├── CS_AmTb77Cm06tzvp001.out
│   │   │   ├── CS_AmTb77Cm06tzvp001.sdf
│   │   │   ├── CS_AmTd189Dfreq001.out
│   │   │   ├── CS_AmTd189Dm06tzvp001.out
│   │   │   ├── CS_AmTd189Dm06tzvp001.sdf
│   │   │   ├── CS_AmTd192Dfreq001.out
│   │   │   ├── CS_AmTd192Dm06tzvp001.out
│   │   │   ├── CS_AmTd192Dm06tzvp001.sdf
│   │   │   ├── CS_AmTd1Dfreq001.out
│   │   │   ├── CS_AmTd1Dm06tzvp001.out
│   │   │   ├── CS_AmTd1Dm06tzvp001.sdf
│   │   │   ├── CS_AmTd790Dfreq001.out
│   │   │   ├── CS_AmTd790Dm06tzvp001.out
│   │   │   ├── CS_AmTd790Dm06tzvp001.sdf
│   │   │   ├── CS_AmTd9Dfreq001.out
│   │   │   ├── CS_AmTd9Dm06tzvp001.out
│   │   │   └── CS_AmTd9Dm06tzvp001.sdf
│   │   ├── LowestR_AR_AmAb1Bfreq001.out
│   │   ├── LowestR_AR_AmAb1Bm06tzvp001.out
│   │   ├── LowestR_AR_AmAb1Bm06tzvp001.sdf
│   │   ├── LowestS_CS_AmTd1Dfreq001.out
│   │   ├── LowestS_CS_AmTd1Dm06tzvp001.out
│   │   └── LowestS_CS_AmTd1Dm06tzvp001.sdf
│   └── Solvent_geometries
│       ├── AR
│       │   ├── AR_AmAb1Nfreq001.out
│       │   ├── AR_AmAb1Nm06tzvp001.out
│       │   ├── AR_AmAb1Nm06tzvp001.sdf
│       │   ├── AR_AmTe1Nfreq001.out
│       │   ├── AR_AmTe1Nm06tzvp001.out
│       │   └── AR_AmTe1Nm06tzvp001.sdf
│       ├── AS
│       │   ├── AS_AmAb1Bfreq001.out
│       │   ├── AS_AmAb1Bm06tzvp001.out
│       │   ├── AS_AmAb1Bm06tzvp001.sdf
│       │   ├── AS_AmT1Bfreq001.out
│       │   ├── AS_AmT1Bm06tzvp001.out
│       │   ├── AS_AmT1Bm06tzvp001.sdf
│       │   ├── AS_AmTd240Bfreq001.out
│       │   ├── AS_AmTd240Bm06tzvp001.out
│       │   └── AS_AmTd240Bm06tzvp001.sdf
│       ├── CR
│       │   ├── CR_AmAd161Mfreq001.out
```

```
│   │   ├── CR_AmAd161Mm06tzvp001.out
│   │   ├── CR_AmAd161Mm06tzvp001.sdf
│   │   ├── CR_AmT132Mfreq001.out
│   │   ├── CR_AmT132Mm06tzvp001.out
│   │   ├── CR_AmT132Mm06tzvp001.sdf
│   │   ├── CR_AmTb1Mfreq001.out
│   │   ├── CR_AmTb1Mm06tzvp001.out
│   │   └── CR_AmTb1Mm06tzvp001.sdf
│   ├── CS
│   │   ├── CS_AmA88Lfreq001.out
│   │   ├── CS_AmA88Lm06tzvp001.out
│   │   ├── CS_AmA88Lm06tzvp001.sdf
│   │   ├── CS_AmAb1Lfreq001.out
│   │   ├── CS_AmAb1Lm06tzvp001.out
│   │   ├── CS_AmAb1Lm06tzvp001.sdf
│   │   ├── CS_AmTd1Lfreq001.out
│   │   ├── CS_AmTd1Lm06tzvp001.out
│   │   └── CS_AmTd1Lm06tzvp001.sdf
│   ├── LowestR_AR_AmTe1Nfreq001.out
│   ├── LowestR_AR_AmTe1Nm06tzvp001.out
│   ├── LowestR_AR_AmTe1Nm06tzvp001.sdf
│   ├── LowestS_CS_AmTd1Lfreq001.out
│   ├── LowestS_CS_AmTd1Lm06tzvp001.out
│   └── LowestS_CS_AmTd1Lm06tzvp001.sdf
├── Fig2_amide
│   ├── I
│   │   ├── CatAfreq001.out
│   │   ├── CatAm06tzvp001.out
│   │   └── CatAm06tzvp001.sdf
│   ├── II
│   │   ├── 1Bfreq001.out
│   │   ├── 1Bm06tzvp001.out
│   │   └── 1Bm06tzvp001.sdf
│   ├── II-III
│   │   ├── TSCHCfreq001.out
│   │   ├── TSCHCm06tzvp001.out
│   │   └── TSCHCm06tzvp001.sdf
│   ├── III
│   │   ├── 2Bfreq001.out
│   │   ├── 2Bm06tzvp001.out
│   │   └── 2Bm06tzvp001.sdf
│   ├── III-IV
│   │   ├── TSCBGfreq001.out
│   │   ├── TSCBGm06tzvp001.out
│   │   └── TSCBGm06tzvp001.sdf
│   ├── IV
│   │   ├── 3Cfreq001.out
│   │   ├── 3Cm06tzvp001.out
│   │   └── 3Cm06tzvp001.sdf
│   ├── V
│   │   ├── 4aCfreq001.out
│   │   ├── 4aCm06tzvp001.out
│   │   └── 4aCm06tzvp001.sdf
│   ├── V-VI
│   │   ├── TSBBDfreq001.out
│   │   ├── TSBBDm06tzvp001.out
│   │   └── TSBBDm06tzvp001.sdf
│   ├── VI
│   │   ├── 5Bfreq001.out
```

```
│   │   ├── 5Bm06tzvp001.out
│   │   └── 5Bm06tzvp001.sdf
│   └── VI-I
│       ├── 5-1Afreq001.out
│       ├── 5-1Am06tzvp001.out
│       └── 5-1Am06tzvp001.sdf
├── Fig2_phosphinamide
│   ├── I
│   │   ├── CatAfreq001.out
│   │   ├── CatAm06tzvp001.out
│   │   └── CatAm06tzvp001.sdf
│   ├── II
│   │   ├── 1Bfreq001.out
│   │   ├── 1Bm06tzvp001.out
│   │   └── 1Bm06tzvp001.sdf
│   ├── II-III
│   │   ├── TS1-2Cfreq001.out
│   │   ├── TS1-2Cm06tzvp001.out
│   │   └── TS1-2Cm06tzvp001.sdf
│   ├── III
│   │   ├── 2Bfreq001.out
│   │   ├── 2Bm06tzvp001.out
│   │   └── 2Bm06tzvp001.sdf
│   ├── III-IV
│   │   ├── TS2-3Cfreq001.out
│   │   ├── TS2-3Cm06tzvp001.out
│   │   └── TS2-3Cm06tzvp001.sdf
│   ├── IV
│   │   ├── 3Bfreq001.out
│   │   ├── 3Bm06tzvp001.out
│   │   └── 3Bm06tzvp001.sdf
│   ├── V
│   │   ├── 4aCfreq001.out
│   │   ├── 4aCm06tzvp001.out
│   │   └── 4aCm06tzvp001.sdf
│   ├── V-VI
│   │   ├── TSBBDfreq001.out
│   │   ├── TSBBDm06tzvp001.out
│   │   └── TSBBDm06tzvp001.sdf
│   ├── VI
│   │   ├── 5Bfreq001.out
│   │   ├── 5Bm06tzvp001.out
│   │   └── 5Bm06tzvp001.sdf
│   └── VI-I
│       ├── 5-1Afreq001.out
│       ├── 5-1Am06tzvp001.out
│       └── 5-1Am06tzvp001.sdf
├── Fig3
│   ├── Cation1Bfreq001.out
│   ├── Cation1Bfreq001_tz.sdf
│   ├── Cation1Bm06tzvp001.out
│   ├── Cation2Bfreq001.out
│   ├── Cation2Bfreq001_tz.sdf
│   ├── Cation2Bm06tzvp001.out
│   ├── Cation3Bfreq001.out
│   ├── Cation3Bfreq001_tz.sdf
│   ├── Cation3Bm06tzvp001.out
│   ├── Cation4Bfreq001.out
│   ├── Cation4Bfreq001_tz.sdf
```

```
│   ├── Cation4Bm06tzvp001.out
│   ├── Cation5Bfreq001.out
│   ├── Cation5Bfreq001_tz.sdf
│   └── Cation5Bm06tzvp001.out
├── Fig5
│   ├── AS_AmA1Ffreq001.out
│   ├── AS_AmA1Ffreq001_tz.sdf
│   ├── AS_AmA1Fm06tzvp001.out
│   ├── AS_AmB1Ffreq001.out
│   ├── AS_AmB1Ffreq001_tz.sdf
│   ├── AS_AmB1Fm06tzvp001.out
│   ├── AS_AmB2Ffreq001.out
│   ├── AS_AmB2Ffreq001_tz.sdf
│   ├── AS_AmB2Fm06tzvp001.out
│   ├── AS_AmT1Ffreq001.out
│   ├── AS_AmT1Ffreq001_tz.sdf
│   ├── AS_AmT1Fm06tzvp001.out
│   ├── AS_AmU1Ffreq001.out
│   ├── AS_AmU1Ffreq001_tz.sdf
│   ├── AS_AmU1Fm06tzvp001.out
│   ├── CS_AmA1Ffreq001.out
│   ├── CS_AmA1Ffreq001_tz.sdf
│   ├── CS_AmA1Fm06tzvp001.out
│   ├── CS_AmB1Ffreq001.out
│   ├── CS_AmB1Ffreq001_tz.sdf
│   ├── CS_AmB1Fm06tzvp001.out
│   ├── CS_AmB2Ffreq001.out
│   ├── CS_AmB2Ffreq001_tz.sdf
│   ├── CS_AmB2Fm06tzvp001.out
│   ├── CS_AmT1Ffreq001.out
│   ├── CS_AmT1Ffreq001_tz.sdf
│   ├── CS_AmT1Fm06tzvp001.out
│   ├── CS_AmU1Ffreq001.out
│   ├── CS_AmU1Ffreq001_tz.sdf
│   └── CS_AmU1Fm06tzvp001.out
├── Fig6
│   ├── Int10Bfreq001.out
│   ├── Int10Bm06tzvp001.out
│   ├── Int10Bm06tzvp001.sdf
│   ├── Int11Bfreq001.out
│   ├── Int11Bm06tzvp001.out
│   ├── Int11Bm06tzvp001.sdf
│   ├── Int1Afreq001.out
│   ├── Int1Am06tzvp001.out
│   ├── Int1Am06tzvp001.sdf
│   ├── Int2Afreq001.out
│   ├── Int2Am06tzvp001.out
│   ├── Int2Am06tzvp001.sdf
│   ├── Int3Afreq001.out
│   ├── Int3Am06tzvp001.out
│   ├── Int3Am06tzvp001.sdf
│   ├── Int4Afreq001.out
│   ├── Int4Am06tzvp001.out
│   ├── Int4Am06tzvp001.sdf
│   ├── Int5Afreq001.out
│   ├── Int5Am06tzvp001.out
│   ├── Int5Am06tzvp001.sdf
│   ├── Int6Afreq001.out
│   ├── Int6Am06tzvp001.out
```

```
│   ├── Int6Am06tzvp001.sdf
│   ├── Int7Bfreq001.out
│   ├── Int7Bm06tzvp001.out
│   ├── Int7Bm06tzvp001.sdf
│   ├── Int8Bfreq001.out
│   ├── Int8Bm06tzvp001.out
│   ├── Int8Bm06tzvp001.sdf
│   ├── Int9Bfreq001.out
│   ├── Int9Bm06tzvp001.out
│   └── Int9Bm06tzvp001.sdf
└── Fig7_amideQ_full_study
    ├── Gas_geometries
    │   ├── AR
    │   │   ├── AR1Dfreq001.out
    │   │   ├── AR1Dm06tzvp001.out
    │   │   ├── AR1Dm06tzvp001.sdf
    │   │   ├── AR2Dfreq001.out
    │   │   ├── AR2Dm06tzvp001.out
    │   │   ├── AR2Dm06tzvp001.sdf
    │   │   ├── AR3Dfreq001.out
    │   │   ├── AR3Dm06tzvp001.out
    │   │   ├── AR3Dm06tzvp001.sdf
    │   │   ├── AR4Dfreq001.out
    │   │   ├── AR4Dm06tzvp001.out
    │   │   ├── AR4Dm06tzvp001.sdf
    │   │   ├── AR5Dfreq001.out
    │   │   ├── AR5Dm06tzvp001.out
    │   │   ├── AR5Dm06tzvp001.sdf
    │   │   ├── AR6Dfreq001.out
    │   │   ├── AR6Dm06tzvp001.out
    │   │   ├── AR6Dm06tzvp001.sdf
    │   │   ├── AR7Bfreq001.out
    │   │   ├── AR7Bm06tzvp001.out
    │   │   ├── AR7Bm06tzvp001.sdf
    │   │   ├── AR8Bfreq001.out
    │   │   ├── AR8Bm06tzvp001.out
    │   │   ├── AR8Bm06tzvp001.sdf
    │   │   ├── AR9Bfreq001.out
    │   │   ├── AR9Bm06tzvp001.out
    │   │   ├── AR9Bm06tzvp001.sdf
    │   │   ├── AR_AmA1132Bfreq001.out
    │   │   ├── AR_AmA1132Bm06tzvp001.out
    │   │   ├── AR_AmA1132Bm06tzvp001.sdf
    │   │   ├── AR_AmA1Bfreq001.out
    │   │   ├── AR_AmA1Bm06tzvp001.out
    │   │   ├── AR_AmA1Bm06tzvp001.sdf
    │   │   ├── AR_AmA56Bfreq001.out
    │   │   ├── AR_AmA56Bm06tzvp001.out
    │   │   ├── AR_AmA56Bm06tzvp001.sdf
    │   │   ├── AR_AmA928Bfreq001.out
    │   │   ├── AR_AmA928Bm06tzvp001.out
    │   │   ├── AR_AmA928Bm06tzvp001.sdf
    │   │   ├── AR_AmT111Bfreq001.out
    │   │   ├── AR_AmT111Bm06tzvp001.out
    │   │   ├── AR_AmT111Bm06tzvp001.sdf
    │   │   ├── AR_AmT117Bfreq001.out
    │   │   ├── AR_AmT117Bm06tzvp001.out
    │   │   ├── AR_AmT117Bm06tzvp001.sdf
    │   │   ├── AR_AmT185Bfreq001.out
```

```
│   │   ├── AR_AmT185Bm06tzvp001.out
│   │   ├── AR_AmT185Bm06tzvp001.sdf
│   │   ├── AR_AmT1Bfreq001.out
│   │   ├── AR_AmT1Bm06tzvp001.out
│   │   └── AR_AmT1Bm06tzvp001.sdf
│   ├── AS
│   │   ├── AS1Efreq001.out
│   │   ├── AS1Em06tzvp001.out
│   │   ├── AS1Em06tzvp001.sdf
│   │   ├── AS2Efreq001.out
│   │   ├── AS2Em06tzvp001.out
│   │   ├── AS2Em06tzvp001.sdf
│   │   ├── AS3Efreq001.out
│   │   ├── AS3Em06tzvp001.out
│   │   ├── AS3Em06tzvp001.sdf
│   │   ├── AS4Efreq001.out
│   │   ├── AS4Em06tzvp001.out
│   │   ├── AS4Em06tzvp001.sdf
│   │   ├── AS5Efreq001.out
│   │   ├── AS5Em06tzvp001.out
│   │   ├── AS5Em06tzvp001.sdf
│   │   ├── AS6Efreq001.out
│   │   ├── AS6Em06tzvp001.out
│   │   ├── AS6Em06tzvp001.sdf
│   │   ├── AS_AmA120Bfreq001.out
│   │   ├── AS_AmA120Bm06tzvp001.out
│   │   ├── AS_AmA120Bm06tzvp001.sdf
│   │   ├── AS_AmA14Bfreq001.out
│   │   ├── AS_AmA14Bm06tzvp001.out
│   │   ├── AS_AmA14Bm06tzvp001.sdf
│   │   ├── AS_AmA1Bfreq001.out
│   │   ├── AS_AmA1Bm06tzvp001.out
│   │   ├── AS_AmA1Bm06tzvp001.sdf
│   │   ├── AS_AmA2Bfreq001.out
│   │   ├── AS_AmA2Bm06tzvp001.out
│   │   ├── AS_AmA2Bm06tzvp001.sdf
│   │   ├── AS_AmA78Bfreq001.out
│   │   ├── AS_AmA78Bm06tzvp001.out
│   │   ├── AS_AmA78Bm06tzvp001.sdf
│   │   ├── AS_AmA90Bfreq001.out
│   │   ├── AS_AmA90Bm06tzvp001.out
│   │   ├── AS_AmA90Bm06tzvp001.sdf
│   │   ├── AS_AmT11Bfreq001.out
│   │   ├── AS_AmT11Bm06tzvp001.out
│   │   ├── AS_AmT11Bm06tzvp001.sdf
│   │   ├── AS_AmT186Bfreq001.out
│   │   ├── AS_AmT186Bm06tzvp001.out
│   │   ├── AS_AmT186Bm06tzvp001.sdf
│   │   ├── AS_AmT1Bfreq001.out
│   │   ├── AS_AmT1Bm06tzvp001.out
│   │   ├── AS_AmT1Bm06tzvp001.sdf
│   │   ├── AS_AmT2316Bfreq001.out
│   │   ├── AS_AmT2316Bm06tzvp001.out
│   │   ├── AS_AmT2316Bm06tzvp001.sdf
│   │   ├── AS_AmT332Bfreq001.out
│   │   ├── AS_AmT332Bm06tzvp001.out
│   │   ├── AS_AmT332Bm06tzvp001.sdf
│   │   ├── AS_AmT94Bfreq001.out
│   │   ├── AS_AmT94Bm06tzvp001.out
```

```
│   │   ├── AS_AmT94Bm06tzvp001.sdf
│   │   ├── AS_AmT968Bfreq001.out
│   │   ├── AS_AmT968Bm06tzvp001.out
│   │   └── AS_AmT968Bm06tzvp001.sdf
│   ├── CR
│   │   ├── CR10Bfreq001.out
│   │   ├── CR10Bm06tzvp001.out
│   │   ├── CR10Bm06tzvp001.sdf
│   │   ├── CR11Bfreq001.out
│   │   ├── CR11Bm06tzvp001.out
│   │   ├── CR11Bm06tzvp001.sdf
│   │   ├── CR1Cfreq001.out
│   │   ├── CR1Cm06tzvp001.out
│   │   ├── CR1Cm06tzvp001.sdf
│   │   ├── CR2Cfreq001.out
│   │   ├── CR2Cm06tzvp001.out
│   │   ├── CR2Cm06tzvp001.sdf
│   │   ├── CR3Cfreq001.out
│   │   ├── CR3Cm06tzvp001.out
│   │   ├── CR3Cm06tzvp001.sdf
│   │   ├── CR4Cfreq001.out
│   │   ├── CR4Cm06tzvp001.out
│   │   ├── CR4Cm06tzvp001.sdf
│   │   ├── CR5Cfreq001.out
│   │   ├── CR5Cm06tzvp001.out
│   │   ├── CR5Cm06tzvp001.sdf
│   │   ├── CR6Cfreq001.out
│   │   ├── CR6Cm06tzvp001.out
│   │   ├── CR6Cm06tzvp001.sdf
│   │   ├── CR7Cfreq001.out
│   │   ├── CR7Cm06tzvp001.out
│   │   ├── CR7Cm06tzvp001.sdf
│   │   ├── CR8Cfreq001.out
│   │   ├── CR8Cm06tzvp001.out
│   │   ├── CR8Cm06tzvp001.sdf
│   │   ├── CR9Bfreq001.out
│   │   ├── CR9Bm06tzvp001.out
│   │   ├── CR9Bm06tzvp001.sdf
│   │   ├── CR_AmA1Efreq001.out
│   │   ├── CR_AmA1Em06tzvp001.out
│   │   ├── CR_AmA1Em06tzvp001.sdf
│   │   ├── CR_AmA31Efreq001.out
│   │   ├── CR_AmA31Em06tzvp001.out
│   │   ├── CR_AmA31Em06tzvp001.sdf
│   │   ├── CR_AmA38Efreq001.out
│   │   ├── CR_AmA38Em06tzvp001.out
│   │   ├── CR_AmA38Em06tzvp001.sdf
│   │   ├── CR_AmA44Efreq001.out
│   │   ├── CR_AmA44Em06tzvp001.out
│   │   ├── CR_AmA44Em06tzvp001.sdf
│   │   ├── CR_AmA522Efreq001.out
│   │   ├── CR_AmA522Em06tzvp001.out
│   │   ├── CR_AmA522Em06tzvp001.sdf
│   │   ├── CR_AmA77Efreq001.out
│   │   ├── CR_AmA77Em06tzvp001.out
│   │   ├── CR_AmA77Em06tzvp001.sdf
│   │   ├── CR_AmT118Cfreq001.out
│   │   ├── CR_AmT118Cm06tzvp001.out
│   │   ├── CR_AmT118Cm06tzvp001.sdf
```

```
│   │   ├── CR_AmT128Cfreq001.out
│   │   ├── CR_AmT128Cm06tzvp001.out
│   │   ├── CR_AmT128Cm06tzvp001.sdf
│   │   ├── CR_AmT132Cfreq001.out
│   │   ├── CR_AmT132Cm06tzvp001.out
│   │   ├── CR_AmT132Cm06tzvp001.sdf
│   │   ├── CR_AmT1Cfreq001.out
│   │   ├── CR_AmT1Cm06tzvp001.out
│   │   ├── CR_AmT1Cm06tzvp001.sdf
│   │   ├── CR_AmT313Cfreq001.out
│   │   ├── CR_AmT313Cm06tzvp001.out
│   │   ├── CR_AmT313Cm06tzvp001.sdf
│   │   ├── CR_AmT43Cfreq001.out
│   │   ├── CR_AmT43Cm06tzvp001.out
│   │   ├── CR_AmT43Cm06tzvp001.sdf
│   │   ├── CR_AmT99Cfreq001.out
│   │   ├── CR_AmT99Cm06tzvp001.out
│   │   └── CR_AmT99Cm06tzvp001.sdf
│   ├── CS
│   │   ├── CS10Dfreq001.out
│   │   ├── CS10Dm06tzvp001.out
│   │   ├── CS10Dm06tzvp001.sdf
│   │   ├── CS11Dfreq001.out
│   │   ├── CS11Dm06tzvp001.out
│   │   ├── CS11Dm06tzvp001.sdf
│   │   ├── CS12Dfreq001.out
│   │   ├── CS12Dm06tzvp001.out
│   │   ├── CS12Dm06tzvp001.sdf
│   │   ├── CS13Dfreq001.out
│   │   ├── CS13Dm06tzvp001.out
│   │   ├── CS13Dm06tzvp001.sdf
│   │   ├── CS1Dfreq001.out
│   │   ├── CS1Dm06tzvp001.out
│   │   ├── CS1Dm06tzvp001.sdf
│   │   ├── CS2Dfreq001.out
│   │   ├── CS2Dm06tzvp001.out
│   │   ├── CS2Dm06tzvp001.sdf
│   │   ├── CS3Dfreq001.out
│   │   ├── CS3Dm06tzvp001.out
│   │   ├── CS3Dm06tzvp001.sdf
│   │   ├── CS4Dfreq001.out
│   │   ├── CS4Dm06tzvp001.out
│   │   ├── CS4Dm06tzvp001.sdf
│   │   ├── CS5Dfreq001.out
│   │   ├── CS5Dm06tzvp001.out
│   │   ├── CS5Dm06tzvp001.sdf
│   │   ├── CS6Dfreq001.out
│   │   ├── CS6Dm06tzvp001.out
│   │   ├── CS6Dm06tzvp001.sdf
│   │   ├── CS7Dfreq001.out
│   │   ├── CS7Dm06tzvp001.out
│   │   ├── CS7Dm06tzvp001.sdf
│   │   ├── CS8Dfreq001.out
│   │   ├── CS8Dm06tzvp001.out
│   │   ├── CS8Dm06tzvp001.sdf
│   │   ├── CS9Dfreq001.out
│   │   ├── CS9Dm06tzvp001.out
│   │   ├── CS9Dm06tzvp001.sdf
│   │   ├── CS_AmA145Dfreq001.out
```

```
│   │   ├── CS_AmA145Dm06tzvp001.out
│   │   ├── CS_AmA145Dm06tzvp001.sdf
│   │   ├── CS_AmA185Dfreq001.out
│   │   ├── CS_AmA185Dm06tzvp001.out
│   │   ├── CS_AmA185Dm06tzvp001.sdf
│   │   ├── CS_AmA1Dfreq001.out
│   │   ├── CS_AmA1Dm06tzvp001.out
│   │   ├── CS_AmA1Dm06tzvp001.sdf
│   │   ├── CS_AmA3Dfreq001.out
│   │   ├── CS_AmA3Dm06tzvp001.out
│   │   ├── CS_AmA3Dm06tzvp001.sdf
│   │   ├── CS_AmA98Dfreq001.out
│   │   ├── CS_AmA98Dm06tzvp001.out
│   │   ├── CS_AmA98Dm06tzvp001.sdf
│   │   ├── CS_AmT14Cfreq001.out
│   │   ├── CS_AmT14Cm06tzvp001.out
│   │   ├── CS_AmT14Cm06tzvp001.sdf
│   │   ├── CS_AmT236Cfreq001.out
│   │   ├── CS_AmT236Cm06tzvp001.out
│   │   ├── CS_AmT236Cm06tzvp001.sdf
│   │   ├── CS_AmT4Cfreq001.out
│   │   ├── CS_AmT4Cm06tzvp001.out
│   │   ├── CS_AmT4Cm06tzvp001.sdf
│   │   ├── CS_AmT530Cfreq001.out
│   │   ├── CS_AmT530Cm06tzvp001.out
│   │   ├── CS_AmT530Cm06tzvp001.sdf
│   │   ├── CS_AmT549Cfreq001.out
│   │   ├── CS_AmT549Cm06tzvp001.out
│   │   ├── CS_AmT549Cm06tzvp001.sdf
│   │   ├── CS_AmT914Cfreq001.out
│   │   ├── CS_AmT914Cm06tzvp001.out
│   │   └── CS_AmT914Cm06tzvp001.sdf
│   ├── LowestR_AR7Bfreq001.out
│   ├── LowestR_AR7Bm06tzvp001.out
│   ├── LowestR_AR7Bm06tzvp001.sdf
│   ├── LowestS_CS1Dfreq001.out
│   ├── LowestS_CS1Dm06tzvp001.out
│   └── LowestS_CS1Dm06tzvp001.sdf
└── Solvent_geometries
    ├── AR
    │   ├── AR7Mfreq001.out
    │   ├── AR7Mm06tzvp001.out
    │   ├── AR7Mm06tzvp001.sdf
    │   ├── AR9Mfreq001.out
    │   ├── AR9Mm06tzvp001.out
    │   ├── AR9Mm06tzvp001.sdf
    │   ├── AR_AmA1Mfreq001.out
    │   ├── AR_AmA1Mm06tzvp001.out
    │   ├── AR_AmA1Mm06tzvp001.sdf
    │   ├── AR_AmT1Mfreq001.out
    │   ├── AR_AmT1Mm06tzvp001.out
    │   └── AR_AmT1Mm06tzvp001.sdf
    ├── AS
    │   ├── AS_AmA2Dfreq001.out
    │   ├── AS_AmA2Dm06tzvp001.out
    │   ├── AS_AmA2Dm06tzvp001.sdf
    │   ├── AS_AmT1Dfreq001.out
    │   ├── AS_AmT1Dm06tzvp001.out
    │   └── AS_AmT1Dm06tzvp001.sdf
```

```
├── CR
│   ├── CR_AmA44Efreq001.out
│   ├── CR_AmA44Em06tzvp001.out
│   ├── CR_AmA44Em06tzvp001.sdf
│   ├── CR_AmT1AIfreq001.out
│   ├── CR_AmT1AIm06tzvp001.out
│   └── CR_AmT1AIm06tzvp001.sdf
├── CS
│   ├── CS1Dfreq001.out
│   ├── CS1Dm06tzvp001.out
│   ├── CS1Dm06tzvp001.sdf
│   ├── CS_AmA14Ffreq001.out
│   ├── CS_AmA14Fm06tzvp001.out
│   ├── CS_AmA14Fm06tzvp001.sdf
│   ├── CS_AmA3Ffreq001.out
│   ├── CS_AmA3Fm06tzvp001.out
│   └── CS_AmA3Fm06tzvp001.sdf
├── LowestR_AR7Mfreq001.out
├── LowestR_AR7Mm06tzvp001.out
├── LowestR_AR7Mm06tzvp001.sdf
├── LowestS_CS1Dfreq001.out
├── LowestS_CS1Dm06tzvp001.out
└── LowestS_CS1Dm06tzvp001.sdf
```

References

1 Genov, G. R.; Douthwaite, J. L.; Lahdenperä, A. S. K.; Gibson, D. C.; Phipps, R. J. Enantioselective Remote C–H Activation Directed by a Chiral Cation. *Science*, **2020**, *367*, 1246–1251.

2 Fanourakis, A.; Williams, B. D.; Paterson K. J.; Phipps, R. J., Enantioselective Intermolecular C-H Amination Directed by a Chiral Cation. *J. Am. Chem. Soc.*, **2021**, *143*, 10070–10076.

3 Lakshminarayana, B.; Mahendar, L.; Ghosal, P.; Sreedhar, B.; Satyanarayana G.; Subrahmanyam, Ch., Fabrication of Pd/CuFe2O4 Hybrid Nanowires: a Heterogeneous Catalyst for Heck Couplings. *New J. Chem.*, **2018**, *42*, 1646–1654.

4 Harder, E.; Damm, W.; Maple, J.; Wu, C.; Reboul, M.; Xiang, J. Y.; Wang L.; Lupyan, D.; Dahlgren, M. K.; Knight, J. L.; Kaus, J. W.; Cerutti, D. S.; Krilov, G.; Jorgensen, W. L.; Abel, R.; Friesner, R. A., OPLS3: A Force Field Providing Broad Coverage of Drug-like Small Molecules and Proteins. *J. Chem. Theory Comput.* **2016**, *12*, 281–296

5 (a) Kolossváry, I. and Guida, W. C., Low-Mode Conformational Search Elucidated: Application To $C_{39}H_{80}$ And Flexible Docking Of 9-Deazaguanine Inhibitors Into PNP. *J. Comput. Chem.*, **1999**, *20*, 1671–1684. (b) Kolossváry, I. and Guida, W. C., Low Mode Search. An Efficient, Automated Computational Method for Conformational Analysis: Application to Cyclic and Acyclic Alkanes and Cyclic Peptides.
J. Am. Chem. Soc., **1996**, *118*, 5011–5019.

6 Gaussian 16, Revision A.03, Frisch, M. J.; Trucks, G. W.; Schlegel, H. B.; Scuseria, G. E.; Robb, M. A.; Cheeseman, J. R.; Scalmani, G.; Barone, V.; Petersson, G. A.; Nakatsuji, H.; Li, X.; Caricato, M.; Marenich, A. V.; Bloino, J.; Janesko, B. G.; Gomperts, R.; Mennucci, B.; Hratchian, H. P.; Ortiz, J. V.; Izmaylov, A. F.; Sonnenberg, J. L.; Williams-Young, D.; Ding, F.; Lipparini, F.; Egidi, F.; Goings, J.; Peng, B.; Petrone, A.; Henderson, T.; Ranasinghe, D.; Zakrzewski, V. G.; Gao, J.; Rega, N.; Zheng, G.; Liang, W.; Hada, M.; Ehara, M.; Toyota, K.; Fukuda, R.; Hasegawa, J.; Ishida, M.; Nakajima, T.; Honda, Y.; Kitao, O.; Nakai, H.; Vreven, T.; Throssell, K.; Montgomery, J. A., Jr.; Peralta, J. E.; Ogliaro, F.; Bearpark, M. J.; Heyd, J. J.; Brothers, E. N.; Kudin, K. N.; Staroverov, V. N.; Keith, T. A.; Kobayashi, R.; Normand, J.; Raghavachari, K.; Rendell, A. P.; Burant, J. C.; Iyengar, S. S.; Tomasi, J.; Cossi, M.; Millam, J. M.; Klene, M.; Adamo, C.; Cammi, R.; Ochterski, J. W.; Martin, R. L.; Morokuma, K.; Farkas, O.; Foresman, J. B.; Fox, D. J. Gaussian, Inc., Wallingford CT, 2016.

7 (a) Becke, A. D. Density-Functional Exchange-Energy Approximation With Correct Asymptotic Behavior. *Phys. Rev. A*, **1988**, *38*, 3098-3100; (b) Lee, C.; Yang, W., Parr, R. G. Development of the Colle-Salvetti Correlation-Energy Formula Into a Functional Of the Electron Density. *Phys. Rev. B.*, **1988**, *37*, 785-789 (c) Becke, A. D. Density-Functional Thermochemistry. III. The Role of Exact Exchange. *J. Chem. Phys.*, **1993**, *98*, 5648- 5652.

8 (a) Hehre, W. J.; Ditchfield, R.; Pople, J. A. Self-Consistent Molecular Orbital Methods. XII. Further Extensions of Gaussian-Type Basis Sets for Use in Molecular Orbital Studies of Organic Molecules. *J. Chem. Phys.*, **1972**, *56*, 2257-2261. (b) Hariharan, P. C.; Pople, J. A. The Influence of Polarization Functions on Molecular Orbital Hydrogenation Energies. *Theor. Chem. Acc.*, **1973**, *28*, 213-222.

9 Dolg, M.; Wedig, U.; Stoll,H.; Preuss,H. Energy-Adjusted Ab Initio Pseudopotentials for the First Row Transition Elements. *J. Chem. Phys.*, **1987**, *86*, 866−872

10 Marenich, A. V.; Cramer, C. J.; Truhlar, D. G. Universal Solvation Model Based on Solute Electron Density and on a Continuum Model of the Solvent Defined by the Bulk Dielectric Constant and Atomic Surface Tensions. *J. Phys. Chem. B*, **2009**, *113*, 6378-6396.

11 Zhao, Y.; Truhlar, D. The M06 Suite of Density Functionals for Main Group Thermochemistry, Thermochemical Kinetics, Noncovalent interactions, Excited States, and Transition Elements: Two New Functionals and Systematic Testing of Four M06-Class Functionals and 12 Other Functionals. *Theor. Chem. Acc.*, **2008**, *120*, 215-241.

12 (a) Weigend, F.; Ahlrichs, R. Balanced Basis Sets of Split Valence, Triple Zeta Valence and Quadruple Zeta Valence Quality for H to Rn: Design and Assessment of Accuracy. *Phys. Chem. Chem. Phys.*, **2005**, *7*, 3297-3305. (b) Weigend, F. Accurate Coulomb-Fitting Basis Sets for H to Rn. *Phys. Chem. Chem. Phys.*, **2006**, *8*, 1057-1065.

13 Luchini, G.; Alegre-Requena, J. V.; Funes-Ardoiz, I.; Paton, R. S. GoodVibes: Automated Thermochemistry for Heterogeneous Computational Chemistry Data. *F1000Research*, **2020**, *9*, 291 DOI: 10.12688/f1000research.22758.1

14 Grimme, S.; Hansen, A.; Ehlert, S.; Mewes, J.-M. r^2SCAN-3c: A "Swiss Army Knife" Composite Electronic-Structure Method. *J. Chem. Phys.* **2021**, *154*, 064103
15 Mardirossian, N.; Head-Gordon, M. ωB97M-V: A Combinatorially Optimized, Range-Separated Hybrid, Meta-GGA Density Functional With VV10 Nonlocal Correlation. *J. Chem. Phys.* **2016**, *144*, 214110
16 Neese, F. Software Update: The ORCA Program System—Version 5.0. *WIREs Comput Mol Sci.* **2022,** *12*, e1606
